# Receptor Quaternary Organization Explains G Protein-Coupled Receptor Family Structure

**DOI:** 10.1016/j.celrep.2017.08.072

**Published:** 2017-09-12

**Authors:** James H. Felce, Sarah L. Latty, Rachel G. Knox, Susan R. Mattick, Yuan Lui, Steven F. Lee, David Klenerman, Simon J. Davis

**Affiliations:** 1 Radcliffe Department of Medicine and Medical Research Council Human Immunology Unit, Weatherall Institute of Molecular Medicine, University of Oxford, Oxford OX3 9DS, UK; 2 Department of Chemistry, University of Cambridge, Cambridge CB2 1EW, UK

**Keywords:** G protein-coupled receptors, stoichiometry, BRET, single-molecule imaging, evolution

## Abstract

The organization of *Rhodopsin*-family G protein-coupled receptors (GPCRs) at the cell surface is controversial. Support both for and against the existence of dimers has been obtained in studies of mostly individual receptors. Here, we use a large-scale comparative study to examine the stoichiometric signatures of 60 receptors expressed by a single human cell line. Using bioluminescence resonance energy transfer- and single-molecule microscopy-based assays, we found that a relatively small fraction of *Rhodopsin*-family GPCRs behaved as dimers and that these receptors otherwise appear to be monomeric. Overall, the analysis predicted that fewer than 20% of ∼700 *Rhodopsin*-family receptors form dimers. The clustered distribution of the dimers in our sample and a striking correlation between receptor organization and GPCR family size that we also uncover each suggest that receptor stoichiometry might have profoundly influenced GPCR expansion and diversification.

## Highlights

• Systematic analysis of 60 *Rhodopsin*-family GPCRs reveals that most are monomers
• *Rhodopsin*-family root ancestor GPCRs are also monomers
• Across families, rates of receptor diversification correlate with stoichiometry
• Skewed family structure suggests dimerization increases receptor “fitness density”

## Graphical Abstract

**Figure:**
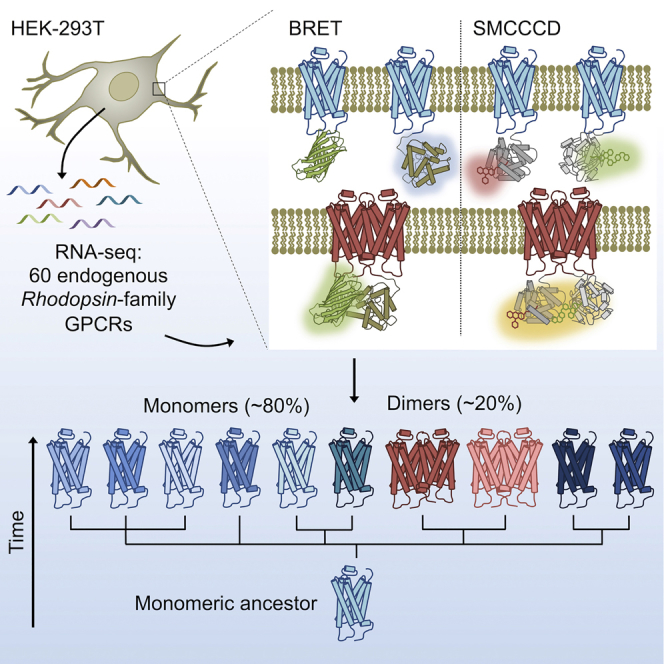

## Teaser

The quaternary organization of *Rhodopsin*-family GPCRs is controversial. Felce et al. show that 60 receptors are mostly monomeric. They propose a simple explanation for the remarkable asymmetry in GPCR family structure, i.e., that it is underpinned by the lineage expansion of monomers rather than dimers.

## Introduction

G protein-coupled receptors (GPCRs) are organized into six main families: the *Glutamate*, *Rhodopsin*, *Adhesion*, *Frizzled*, *Secretin*, and *Taste2* families (Fredriksson et al., 2003). A striking feature of GPCR family structure is the overwhelming dominance of the *Rhodopsin* (class A) family, which comprises >80% of all human GPCRs and a similar fraction of the GPCRs expressed by other vertebrates (Fredriksson and Schioth, 2005). GPCRs all consist of a core of seven transmembrane (TM) α helices joined by six interhelical loops of variable length. The loops combine with the N and C termini forming, respectively, an extracellular region that, together with the TM region, creates the ligand-binding site and a cytoplasmic region that interacts with secondary signaling components, e.g., G proteins. The organization of the TM region is strikingly similar across all GPCRs for which structures have been obtained and is stabilized by a conserved network of interactions between topologically equivalent residues (Venkatakrishnan et al., 2013). The most significant structural variation between GPCRs is restricted to the ligand-binding regions, and the parts of the receptors involved in signal transduction are typically much more highly conserved (Katritch et al., 2012), allowing similar conformational changes to accompany receptor activation (Deupi and Standfuss, 2011). Several studies of isolated GPCRs (Bayburt et al., 2007, Ernst et al., 2007, Kuszak et al., 2009, Leitz et al., 2006, Whorton et al., 2007) convincingly show that signal transduction can occur on the scale of single, autonomous receptors, consistent with GPCRs forming 1:1 complexes with G proteins (Rasmussen et al., 2011).

Without question, the most contentious aspect of GPCR biology concerns their quaternary structures. This is not an insignificant issue, as homo- or hetero-oligomer formation offers, e.g., a simple explanation for a wealth of pharmacological data implying that receptors engage in “cross-talk” (although other explanations are possible; Chabre et al., 2009, Tubio et al., 2010) and new opportunities for pharmacological intervention. Whereas several small families of GPCRs comprise receptors whose large N- and C-terminal domains are known to effect dimerization, e.g., the *Glutamate* (class C) receptors (Gurevich and Gurevich, 2008b), there is no consensus regarding the “typical” quaternary structure of the largest group of GPCRs, i.e., the *Rhodopsin* family. It was initially thought that *Rhodopsin*-family GPCRs are generally monomeric, but the more prevalent view now (Pétrin and Hebert, 2012, Pfleger and Eidne, 2005) is that these receptors form transiently associating or stable dimeric and oligomeric complexes, with implications for their signaling behavior (the cases for and against oligomerization have been summarized by Bouvier and Hebert [2014] and by Lambert and Javitch [2014]). The first applications of resonance energy transfer (RET)-based assays seemed to precipitate this shift in thinking, but these assays are prone to difficulties in distinguishing genuine interactions from chance co-localizations, and the interpretation of some early studies is disputed (Chabre et al., 2009, Chabre and le Maire, 2005, Felce and Davis, 2012, James et al., 2006). More recently, single-molecule measurements have failed to demonstrate constitutive oligomerization in transfected and native cells (Cai et al., 2017, Hern et al., 2010, Jonas et al., 2015, Kasai et al., 2011, Latty et al., 2015, Nenasheva et al., 2013), with one exception (Calebiro et al., 2013). Equally, lattice contacts in GPCR crystals tend to argue against dimeric interactions, and, where putative dimers have been observed, the proposed interfaces were not conserved (Huang et al., 2013, Manglik et al., 2012, Salom et al., 2006, Wu et al., 2010). It remains a possibility, however, that dimeric *Rhodopsin*-family GPCRs exist, but only rarely (e.g., Gurevich and Gurevich, 2008a). With few exceptions (e.g., Calebiro et al., 2013), differences in receptor behavior have not been reported in individual studies, although systematic, comparative analyses of GPCR organization have not been undertaken.

Of the RET-based approaches for studying GPCR stoichiometry, which still represent the highest resolution (<10 nm) in situ assays, bioluminescence RET (BRET) is the most widely used, because it is relatively straightforward and uncomplicated by photobleaching and photoconversion effects confounding Förster RET-based measurements. We have established three BRET-based assays (types-1 to -3; Felce et al., 2014, James et al., 2006), each indicating that human β_2_-adrenergic receptor (β_2_AR) and mouse cannabinoid receptor 2 (mCannR2) are monomers. Here, we report a systematic analysis of the stoichiometry of 60 *Rhodopsin-*family GPCRs using two of these assays and a single-molecule fluorescence-based assay (Latty et al., 2015). We found (1) that a small fraction of *Rhodopsin*-family GPCRs formed authentic dimers and that these receptors were otherwise monomeric, (2) that dimers comprised closely related phylogenetic clusters, (3) that these receptor clusters did not share ligand or G protein selectivity outside the clusters, and (4) that even closely related receptors could have different stoichiometries. These findings suggest a simple explanation for the remarkable asymmetry in GPCR family structure, i.e., that it is underpinned by the lineage expansion of monomers rather than dimers.

## Results

### BRET Assay Sensitivity

The BRET assays used in this study are described in detail elsewhere (Felce et al., 2014, James et al., 2006). Briefly, in type-1 BRET experiments (James et al., 2006), the ratio of acceptor- to donor-tagged proteins is varied at constant expression, resulting in a hyperbolic relationship between energy transfer efficiency (BRET_eff_) and acceptor:donor ratio for dimers (the principles of the assays are illustrated in Figure S1A). For monomers, as confirmed for type-1 receptors of known stoichiometry (James et al., 2006), BRET_eff_ is effectively independent of this ratio above a certain threshold. Stoichiometry is indicated by R^2^ values for the data fitted to monomer versus dimer models (Figures S1B and S1C). In type-3 BRET assays, untagged “competitor” receptors reduce BRET_eff_ for dimers, but not monomers, for a range of expression levels (Figure S1A), with stoichiometry confirmed by the likelihood (p^diff^) that BRET_eff_ is affected by the competitor (Figure S1D; Felce et al., 2014). These assays are complementary insofar as type-1 assays are not prone to false-dimer artifacts but could, in principle, give false-monomer results in cases of higher order oligomerization or very weak dimerization, whereas type-3 assays avoid false-monomer results but could produce false-dimer signals, e.g., when the addition of competitor proteins causes the clustering of tagged receptors to be relaxed, reducing effective density (Figure S1A). Concordant data obtained with these assays, therefore, afford confident assignment of receptor stoichiometry.

We undertook a systematic exploration of *Rhodopsin-*family GPCR stoichiometry using both type-1 and -3 BRET assays. By focusing on the set of GPCRs expressed by HEK293T cells, the host cell typically used for BRET assays (Pfleger and Eidne, 2005), we could characterize receptor behavior in its native cellular milieu. The choice of cell line precluded the use of type-2 assays reliant on observations made at very low expression levels (James et al., 2006), because this type of analysis would have been complicated by the presence of untagged, native receptors. As reported previously (e.g., Barak et al., 1997), C-terminal tagging of two example GPCRs (i.e., human β_1_-adrenergic receptor [β_1_AR] and β_2_AR) with GFP and Rluc did not alter their responses to agonists (Figure S2A). The sensitivity of the type-1 and -3 BRET assays was first tested using an inducible system for generating dimers. The monomeric receptor CD86 was fused to the FK506-binding protein (FKBP), allowing the bivalent FKBP ligand AP20187 to induce various levels of receptor dimerization (Figures S2B and S2C). Type-1 and -3 BRET assays detected dimers comprising as few as 20% of the total receptor population (Figures S2D–S2F and S2I–S2Q); similar data were obtained for induced β_2_AR dimers (Figures S2D, S2G, S2H, and S2R–S2Z). For simplicity, we hereinafter refer to two classes of receptors: “dimers” (≥20% dimerization) and “monomers” (<20% dimerization).

### Two Types of *Rhodopsin*-Family GPCR Behavior

HEK293T-cell-expressed GPCRs were identified by mining the Universal Protein Resource (UniProt) database (www.uniprot.org) and comparing the results to gene expression data generated by deep sequencing (RNA sequencing; RNA-seq) of the HEK293T cell transcriptome. mRNA encoding 65 *Rhodopsin*-family GPCRs was detected in HEK293T cells (Data S1, “Receptors”), with assignment of receptors to the *Rhodopsin* family based mostly on published phylogenetic analyses (Fredriksson et al., 2003). These receptors comprised a cross-section of *Rhodopsin*-family GPCRs, with a diverse range of physiological functions and ligands, although some important receptor families were not represented, e.g., the dopamine receptors, and some receptors may only exist at the RNA level in HEK293T cells. Transient transfection of GPCRs in the form of GFP fusion proteins in HEK293T cells gave expression levels of ∼100,000 per cell (Data S1, “BRET Experiments”), as determined by flow-cytometric analysis. This included expression on intracellular membranes, consistent with many GPCRs residing mostly in internal membranes until stimulated to traffic to the cell surface (e.g., Brismar et al., 1998, Hein et al., 1994). These expression levels were 1–2 orders of magnitude higher than that of native receptors (e.g., Hegener et al., 2004, Nenasheva et al., 2013), avoiding interference of the assays by homo- and heteromeric interactions with native receptors.

**Figure 1:**
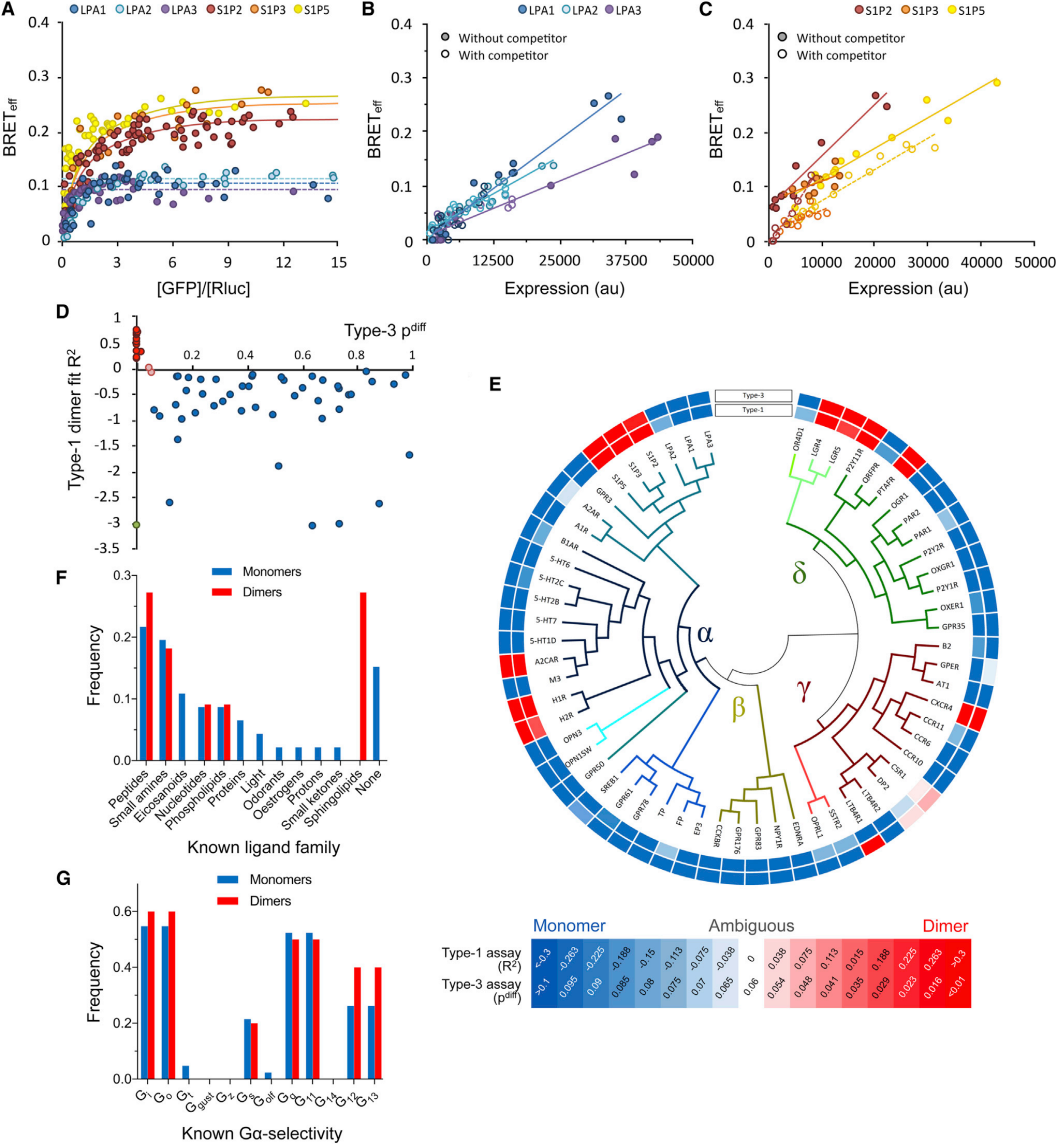
Two Stoichiometric Classes of *Rhodopsin*-Family GPCRs Revealed by Type-1 and -3 BRET Assays (A) Type-1 BRET data for HEK293T-cell-expressed S1P and LPA receptors as representatives of two stoichiometric classes of *Rhodopsin*-family GPCRs. Optimal fits of the data are shown as a solid line for a dimer model, and as a broken line for a monomer model. (B) Type-3 BRET data for the LPA receptors, revealing monomeric behavior consistent with the type-1 analysis. Data collected in both the absence and presence of competitor proteins are shown as filled and empty circles, respectively. A single fit of all data is shown as a solid line. (C) Type-3 BRET data for the S1P receptors, revealing dimeric behavior consistent with the type-1 analysis. Data fits are shown as solid lines (no competitor) and broken lines (with competitor). (D) R^2^ (type-1 assay) and p^diff^ (type-3 assay) values for *Rhodopsin*-family GPCRs. Monomers are indicated in blue; dimers are indicated in red. Dimers cluster in the region of high R^2^ and low p^diff^. C5R1 and DP2 (pink) are ambiguous, because they lie near the boundary for monomer versus dimer identification in both assays. LTB4R1 (green) is the only receptor to yield conflicting results in the two assays, possibly due to high-order oligomerization. (E) Outcomes for all *Rhodopsin*-family receptors investigated. A gradient (from monomer, in blue, to dimer, in red) is colored according to the R^2^ and p^diff^ values shown in the key. The inner circle gives the type-1 assay result; the outer circle gives the type-3 assay result. Receptor relationships are shown as a divergence tree (Fredriksson et al., 2003). (F) Known ligand preferences of the monomeric and dimeric receptors. (G) G protein selectivity of the monomers and dimers. See also Figures S1, S2, S3, and S4, and Data S1 (“BRET Experiments”).

Of the 65 receptors, 60 had sufficiently good trafficking and expression characteristics to allow BRET analysis (Figure S3; Table S1). Type-1 and type-3 BRET analysis of 57 of the 60 GPCRs yielded concordant data suggesting that *Rhodopsin*-family GPCRs exist in two stoichiometric states. Representative datasets for the monomeric lysophosphatidic acid (LPA) and dimeric sphingosine-1-phosphate (S1P) receptors are shown in Figures 1A–1C. The R^2^ and p^diff^ values are plotted in Figure 1D, and absolute values are given in Data S1 (“BRET Experiments”). The results of the analysis, arranged according to *Rhodopsin*-family substructure, are shown schematically in Figure 1E. For each type-1 assay, total receptor expression was independent of [GFP]/[Rluc] (Data S1, “BRET Experiments”) as required by the method (Figure S1A). In this assay, the maximum energy transfer efficiency (BRET_max_) correlated with stoichiometry (i.e., typically higher for dimers than for monomers) and, to a lesser extent, with total expression level (Figure S4A; Data S1, “BRET Experiments”). In the type-3 assay, all dimers gave significantly non-zero projected y-intercepts, whereas, for the monomers, the y-intercepts were not significantly non-zero (Figure S4B; Data S1, “BRET Experiments”). It needs to be noted, however, that the ab initio interpretation of BRET_max_ and the y-intercepts is not straightforward. BRET_max_ does not directly report the extent of dimerization and is, instead, determined by many factors, including receptor density, clustering, and subunit geometry. The type-3 assay y-intercept is also subject to confounding effects arising from high expression. Therefore, while consistent with our assignments, these metrics were not used to assign stoichiometry to individual receptors.

Of the 60 receptors investigated, 46 exhibited monomeric behavior in both assays, indicating that they were either wholly monomeric or formed dimers at levels below the sensitivity of the assays. Eleven receptors behaved as dimers in both assays. Two receptors, C5R1 and DP2, yielded data that could not be easily assigned to either form of behavior, possibly due to dimerization at levels near the sensitivity limits of the assays. Only one receptor, LTB4R1, yielded conflicting data in the two assays, perhaps due to high-order oligomerization, which cannot be unambiguously excluded by the type-1 assay but is readily detected in type-3 assays. LTB4R1 was the only receptor for which data suggestive of high-order oligomerization was obtained, and it is unclear whether it is a bona fide oligomer and/or whether other *Rhodopsin*-family GPCRs can form high-order oligomers. The monomeric and dimeric populations exhibit no obvious differences in either ligand- or G protein specificity (Figures 1F and 1G). Moreover, the stoichiometric assignments do not correlate with C-terminal domain length (Figure S4C), implying that the cytoplasmic domains did not impose donor-acceptor separation distances greater than the RET-permissive radius (10 nm), leading to the false identification of monomers. Conversely, dimerization is not explained as an artifact of poor trafficking or expression, since the fraction of dimers did not correlate with apparent trafficking behavior (Figure S4D). Similarly, although heterodimerization with native GPCRs and/or TM proteins could conceivably restrict homodimerization, this seems very unlikely at the high levels of receptor expression used for BRET assays. For the dimers, we are unable to speculate about the strength of dimerization, as our analysis produces binary outcomes (i.e., fits that are closer to either a monomer or dimer model); however, a range of dimer stabilities could reasonably be expected. Models of partial dimerization could, in principle, be fitted to our data but would not be informative within the confidence limits of the assay.

### Single-Molecule Analysis

To further confirm that *Rhodopsin*-family GPCRs exist in more than one stoichiometric state, we used single-molecule cross-color coincidence detection (SMCCCD; Latty et al., 2015). Briefly, candidate GPCRs were transiently expressed in Chinese hamster ovary (CHO) K1 cells under the control of a weak promoter to ensure approximately physiological, i.e., low levels of expression. Unlike HEK293T cells, CHO K1 cells do not express homologs of any of the receptors studied using SMCCCD (Baycin-Hizal et al., 2012), thereby avoiding interference with the single-molecule analysis. Constructs consisting of the receptor fused with a C-terminal HaloTag or a SNAP-tag were co-expressed and then labeled with HaloTag-TMR Ligand and SNAP-Cell 505 Star. This allowed individual receptors to be localized each in one of two colors, and the degree of co-localization to be compared to the known monomeric and dimeric controls, CD86 and CD28, respectively. Coincidence values represent the fraction of HaloTag-labeled receptors that localize to within 300 nm of a SNAP-tag labeled receptor, presented as the mean for all cells analyzed. The principle of the method is summarized in Figure S1A.

**Figure 2:**
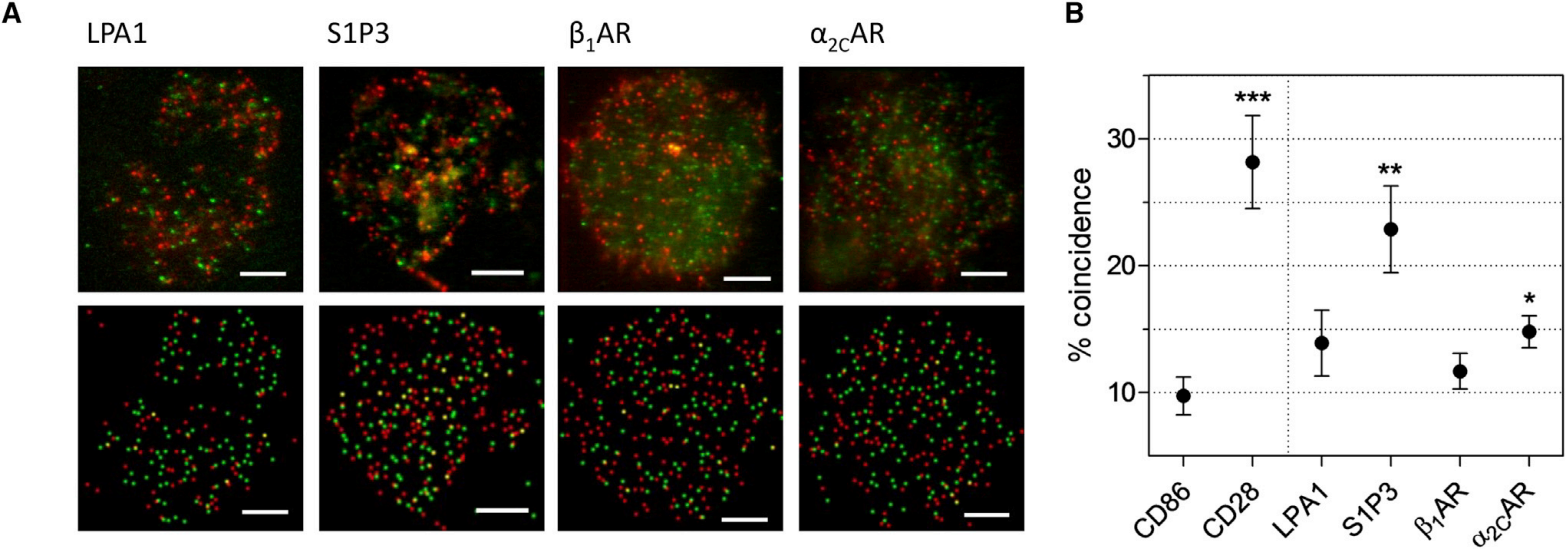
Single-Molecule Microscopy Confirms the Existence of Two Stoichiometric Classes of *Rhodopsin*-Family GPCRs (A) Representative actual data (top) and reconstructed spot detection (bottom) for transfected HaloTag-labeled (red) and SNAP-tag-labeled (green) proteins expressed in CHO K1 cells. Scale bars, 5 μm. LPA1, S1P3, β_1_AR, and α_2C_AR were expressed as C-terminal SNAP-tag- and HaloTag-fusion proteins for two-color labeling. (B) Cross-color coincidence values for GPCRs investigated using SMCCCD. S1P3 and α_2C_AR exhibit levels of coincidence significantly higher than that for the strict monomer, CD86, but lower than that for the covalent dimer, CD28. Coincidence values for both LPA1 and β_1_AR were not significantly higher than that for CD86. The data confirmed the observations made using BRET. Error bars indicate mean ± SE. ^∗^p < 0.05; ^∗∗^p < 0.01; ^∗∗∗^p < 0.005 (two-tailed t test of difference to CD86). See also Table S2.

Receptors exhibiting contrasting behavior in the BRET assays were selected for the SMCCCD analysis: both the S1P receptor 3 (S1P3) and α_2C_-adrenergic receptor (α_2C_AR) behaved as dimers, whereas LPA receptor 1 (LPA1) and β_1_-adrenergic receptor (β_1_AR) exhibited monomeric behavior. Consistent with the BRET analysis, in the single-molecule assay, S1P3 and α_2C_AR exhibited above-background levels of cross-color coincidence characteristic of dimers, whereas the LPA1 and β_1_AR receptors exhibited monomer control levels of cross-color coincidence (Figure 2; Table S2). The measured coincidence level was considerably higher for S1P3 than for α_2C_AR, however, which was only slightly higher than that for the monomer control, CD86. It is, therefore, possible that, at physiological expression levels, α_2C_AR is only a weak dimer. We cannot formally exclude the possibility that the coincidence observed in this assay is the product of indirect receptor co-localization rather than direct physical association, given that our observations are diffraction limited to a resolution of ∼300 nm, ∼60-fold larger than the hydrodynamic diameter of most GPCRs. However, this would not be consistent with the results of the BRET assays.

### Dimerization of S1P3 via TM Helix 4

**Figure 3:**
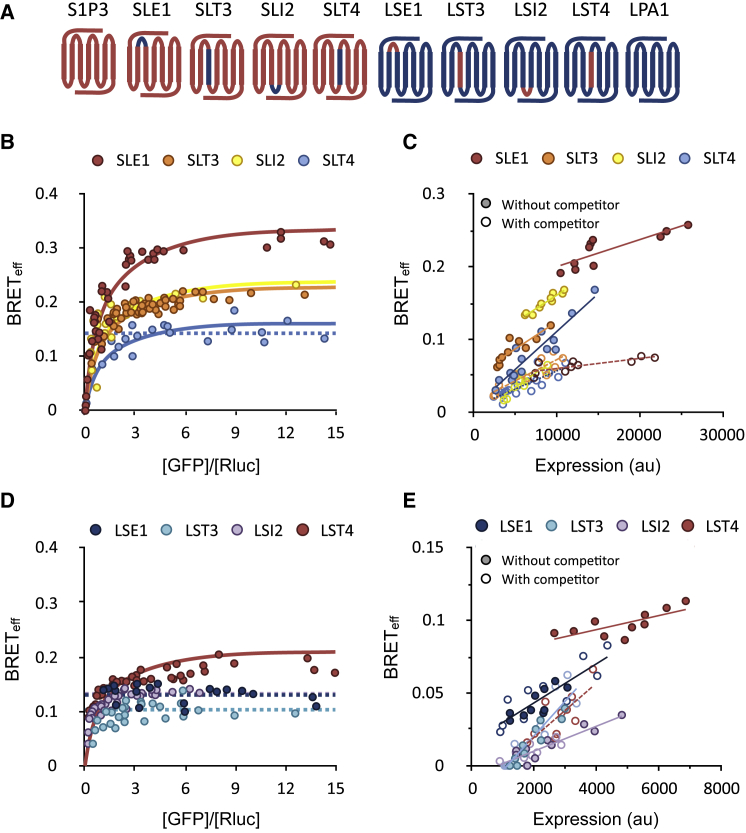
S1P3 Dimerization Is Mediated Primarily by Interactions Involving TM4 (A) Schematic representation of the S1P3/LPA1 chimeras studied, indicating the contributions of the S1P3 (red) and LPA1 (blue) sequences. TM helices are arranged from 1 to 7, left to right. (B) Type-1 BRET analysis of S1P3 chimeras containing LPA1 domains. The data indicate that all of the constructs formed dimers, with SLT4 exhibiting the worst fit to a dimer model, suggesting weaker dimerization. For SLT4, the fit to a monomer model is also shown (broken line). (C) Type-3 BRET analysis of S1P3 chimeras containing LPA1 domains, confirming the findings of the type-1 analysis. (D) Type-1 BRET analysis of LPA1 chimeras containing S1P3 domains. Only LST4, with the TM4 domain of S1P3, formed detectable dimers. (E) Type-3 BRET analysis of LPA1 chimeras containing S1P3 domains, confirming the findings of the type-1 analysis. See also Figure S5 and Data S1 (“BRET Experiments”).

The contrasting behavior of the otherwise closely related LPA and S1P subgroups allowed coarse mapping of the dimerization interface. In preliminary experiments, three LPA1/S1P3 chimeras were generated with various contributions from each receptor (Figure S5A), and these expressed well enough for BRET analysis (Figure S5B). Type-1 BRET analysis of the chimeras indicated that the presence of the N-terminal domain, TM helix 1, intracellular loop (IL) 1, and TM2 of S1P3 (“chimera 1”) were not sufficient to induce LPA1 to form dimers, whereas the inclusion also of extracellular loop (EL) 1, TM3, IL2, and TM4 (“chimera 2”) resulted in chimera dimerization (Figure S5C; Data S1, “BRET Experiments”). Including additional S1P3 domains had no further effect on receptor stoichiometry (“chimera 3”; Figure S5C). This suggested that the sequence motifs mediating interaction in S1P3 are likely predominantly located within the EL1, TM3, IL2, and TM4 regions. The contributions of these regions to S1P3 dimerization were further dissected using reciprocal swaps of the individual domains (Figure 3A). Type-1 and type-3 BRET assays identified TM4 as the principal site of dimerization, since the transfer of LPA1 TM4 to S1P3 partially abrogated S1P3 dimerization (Figures 3B and 3C), and transfer of S1P3 TM4 induced LPA1 dimerization (Figures 3D and 3E; Data S1, “BRET Experiments”).

### Correlation of GPCR Family Size and Stoichiometry

**Figure 4:**
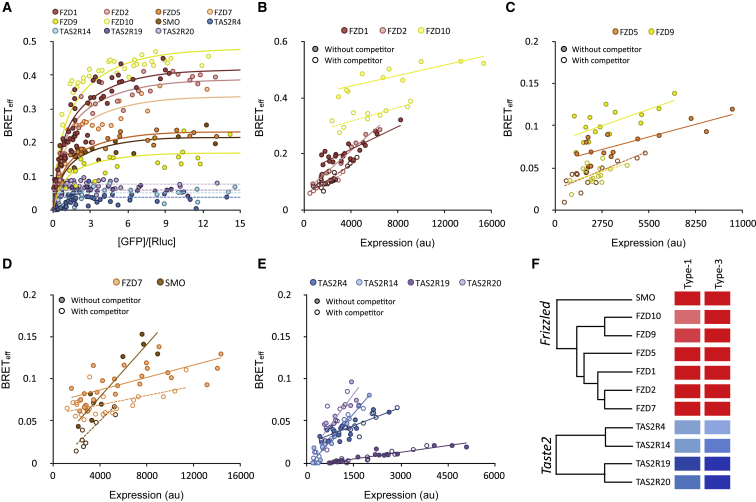
*Fizzled*- and *Taste2*-Family GPCRs Comprise Monomers and Dimers, Respectively (A) Type-1 BRET analysis of seven *Frizzled* and four *Taste2* receptors. The *Frizzled* receptors all formed dimers, whereas the *Taste2* receptors behaved as monomers. (B) Type-3 BRET analysis of the FZD1, FZD2, and FZD10 receptors, confirming the findings of the type-1 analysis. (C) Type-3 BRET analysis of FZD5 and FZD9. (D) Type-3 BRET analysis of FZD7 and SMO. (E) Type-3 BRET analysis of the *Taste2* receptors. (F) Results of the *Frizzled* and *Taste2* receptor assays summarized as in Figure 1E. See also Figure S5 and Data S1 (“BRET Experiments”).

Our analysis reveals that most *Rhodopsin*-family GPCRs expressed by HEK293T cells are monomers. The phylogenetic distribution of the dimers that we have identified appears to be non-random, however. Of the eleven dimers, seven are closely related to other dimers (Figure 1E), forming clusters: the S1P receptors S1P2, S1P3, and S1P5; the histamine receptors H1R and H2R; and the leucine-rich repeat-containing receptors LGR4 and LGR5. In addition to reinforcing the BRET-based assignments, these data suggest that the evolutionary appearance of dimers might be rare and episodic. Moreover, the contrasting stoichiometries of the large *Rhodopsin* family and the small *Glutamate* family (Gurevich and Gurevich, 2008b) are suggestive of there being a correlation between predominantly dimeric behavior and restricted family size. To test whether stoichiometry correlates with GPCR family size, we examined the *Frizzled* GPCRs, which appeared contemporaneously with *Rhodopsin*-family GPCRs but comprise only 11 receptors, and *Taste2* GPCRs, which emerged and separated from the *Rhodopsin* family just ∼300 million years ago but already comprise more than 28 members (Nordstrom et al., 2011) exhibiting significant diversification (Kim et al., 2005). Seven *Frizzled* and four *Taste2* HEK293T-derived GPCRs expressed well enough for BRET analysis (Figure S3). In type-1 and -3 assays, the *Frizzled* receptors all behaved as dimers, whereas the *Taste2* receptors exhibited only monomeric behavior (Figure 4; Data S1, “BRET Experiments”), suggesting that fast-diverging receptors might generally be monomeric and that receptors exhibiting less diversification are more often dimers. Chimeric receptors in which the N-terminal, C-terminal, and TM domains of a *Frizzled* (FZD10) receptor and a *Taste2* (TAS2R19) receptor were recombined implicated the N- and C-terminal regions of FZD10 in its dimerization, rather than the TM region (Figures S5D–S5F; Data S1, “BRET Experiments”).

### Stoichiometry of *Rhodopsin*-Family Root-Ancestor GPCRs

**Figure 5:**
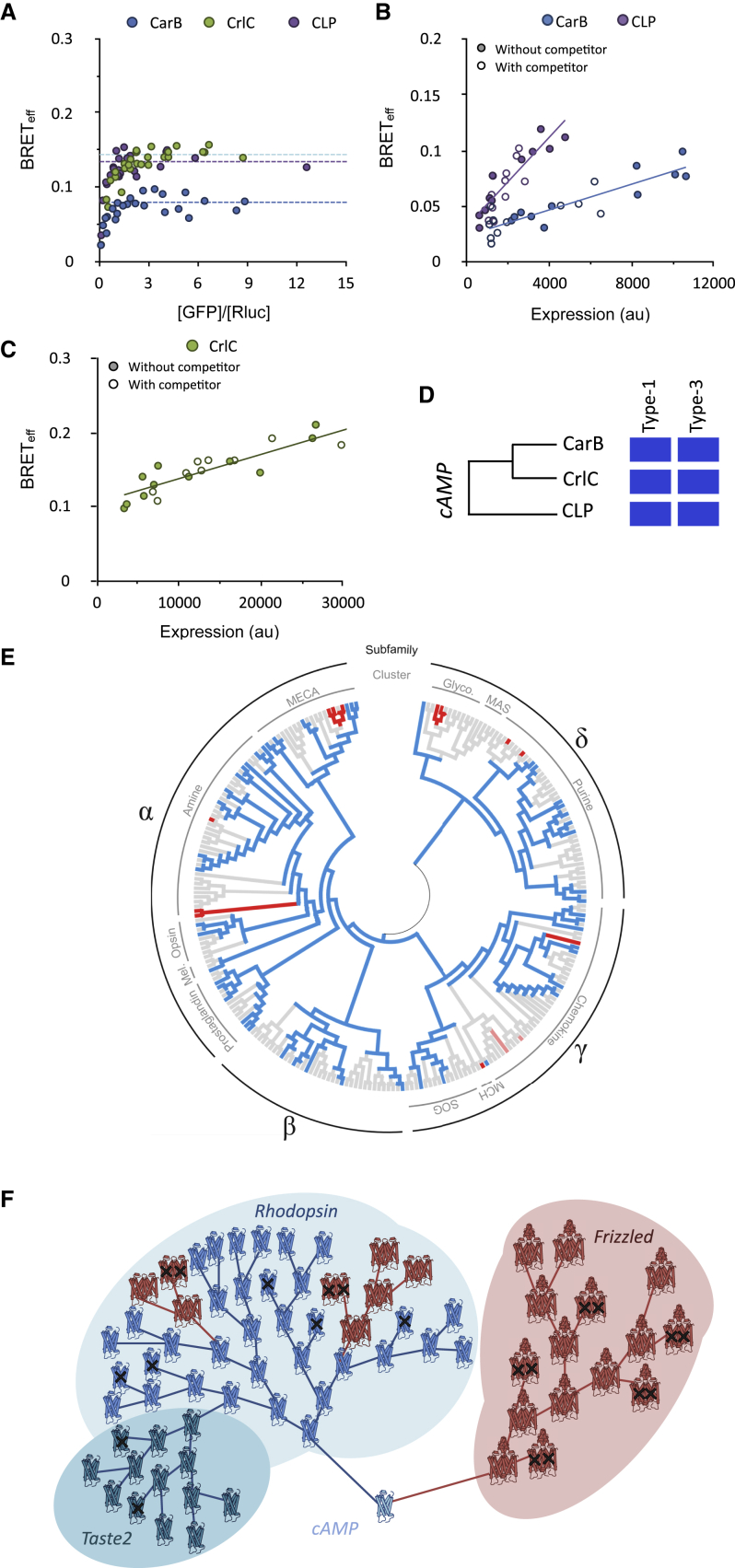
Root Ancestor *cAMP*-Family Receptors Behave as Monomers in Type-1 and -3 BRET Assays (A) Type-1 BRET analysis of three *cAMP*-family receptors. (B) Type-3 BRET analysis of CarB and CLP. (C) Type-3 BRET analysis of CrlC. (D) Results of the assays summarized as in Figure 1E. (E) Modeled lineage tree for the *Rhodopsin* family, highlighting the episodic nature of gain-of-dimerization events. The tree shows all non-olfactory *Rhodopsin*-family GPCRs along with their subfamilies and clusters (Fredriksson et al., 2003). Colored branch endpoints indicate receptors investigated in this study: red for dimers, blue for monomers, pink for C5R1 and DP2. Receptors on gray branches were not investigated. Nodes are colored according to their predicted stoichiometry assuming the spontaneous emergence of dimers. The distribution of *Rhodopsin*-family dimers suggests that ≥ 7 independent gain-of-dimerization or stabilization-of-dimerization events have occurred during the evolution of this family of receptors. (F) Evolutionary model of GPCR family expansion in which dimers (red) are constrained in their diversification compared to monomers (blue). Receptors carrying deleterious mutations are marked with a cross. These receptors either are lost during natural selection or degrade into pseudogenes over time. Relationships between receptors are represented as a simple lineage tree. See also Data S1 (“BRET Experiments”).

The *Rhodopsin* family is thought to have emerged ∼1.3 billion years ago from the *cAMP* GPCR family (Nordstrom et al., 2011), which was subsequently lost from vertebrates. We examined the stoichiometry of three root-ancestor, non-vertebrate *cAMP* family GPCRs in type-1 and type-3 BRET assays. CrlC and CarB are from *Dictyostelium discoideum*, and a receptor we call CLP (*cAMP*-like receptor in Paramecium) is the sole *cAMP*-like GPCR expressed by *Paramecium tetraurelia*. We expect that, because the two species are evolutionarily distant (*D. discoideum* belongs to the amoebozoans, and *P. tetraurelia* belongs in the chromalveolata kingdom), similarities in their behavior will likely reflect the properties of *cAMP* GPCRs generally. All three *cAMP*-family receptors exhibited monomeric behavior in the two BRET assays (Figures 5A–5D; Data S1, “BRET Experiments”).

## Discussion

The stoichiometry of *Rhodopsin*-family GPCRs has been very contentious. Part of the controversy has centered on how best to implement RET measurements in studies of these receptors (Bouvier et al., 2007, James and Davis, 2007, James et al., 2006, Salahpour and Masri, 2007). Acquired in differently formatted assays, BRET data were used, for example, to support claims that β_2_AR is an obligate dimer (Angers et al., 2000, Mercier et al., 2002, Ramsay et al., 2002) or that it is constitutively monomeric (Felce et al., 2014, James et al., 2006). *Rhodopsin*-family monomers and dimers were never identified in any single study, however, leaving open the formal possibility that these assays are incapable of detecting both types of behavior. Here, using two complementary BRET-based assays, we identified multiple examples of *Rhodopsin*-family GPCRs, each exhibiting one of two distinct types of behavior, one characteristic of substantive dimers and the other characteristic of monomers. For pairs of monomers and dimers identified in the BRET assays, we observed the same type of behavior in a third, orthogonal single-molecule fluorescence-based assay. For one of the dimers, we tentatively identified the core of the dimerizing interface as TM helix 4 of the receptor. These data, therefore, strongly suggest that *Rhodopsin*-family GPCRs comprise both monomers and dimers, with the more “typical” behavior being that of a monomer. Although we cannot draw direct conclusions regarding ligand-induced dimerization from these data, we anticipate that the observed resting-state behavior of the monomers likely reflects a general tendency toward constitutive monomeric behavior. Similarly, whether the dimers we detect can also heterodimerize with closely related monomeric or dimeric receptors, as has been claimed for CXCR4 (e.g., Contento et al., 2008), remains to be determined.

Our analysis indicates that as many as 20% of the ∼700 *Rhodopsin*-family receptors may form dimers, distributed in discrete clusters across the family (Figure 5E). We did not attempt to measure the strength of dimerization, and it is unclear what fraction of these receptors, if any, dimerize constitutively rather than transiently. S1P3, which, at very low expression levels in the SMCCCD assay, produced a coincidence signal indistinguishable from that of our covalent dimer control, CD28, seems to be a good candidate for constitutive receptor homodimerization, however, as does CXCR4, given the very high BRET_max_ measured in our type-1 assay. On the other hand, the BRET measurements were performed under conditions of very high expression, and it is possible that, due to the effects of mass action, a smaller fraction of *Rhodopsin*-family GPCRs dimerize at lower levels of physiological expression. This behavior seems to be exemplified by α_2C_AR. In the BRET experiments, α_2C_AR exhibited clear-cut dimeric behavior, but only marginally higher levels of co-association than our control monomer, CD86, at the more physiological expression levels of the SMCCCD experiments. For the large set of receptors we sampled using BRET, we are, therefore, likely to have identified the upper limit of the fraction that form dimers. That only one receptor, LTB4R1, gave data suggestive of higher order oligomerization implies that such structures could be very rare.

Our observations are, nevertheless, strongly at odds with the notion that *Rhodopsin-*family GPCRs form constitutive dimers. It is noteworthy that, of 26 receptors reported previously to be dimers, often in multiple publications (Table S3), 21 behaved as monomers in our assays. Our data are in closer agreement with the increasing number of single-molecule microscopy experiments that have failed to identify constitutive dimers in most cases (AbdAlla et al., 2001, Hern et al., 2010, Jonas et al., 2015, Kasai et al., 2011, Kuszak et al., 2009, Latty et al., 2015, Nenasheva et al., 2013). The present data also argue against a general model of allosteric regulation of GPCR homodimers founded, principally, upon pharmacological analysis. Instead, cooperative effects observed between receptors could arise from indirect cross-talk between monomers. Such effects could be caused in vitro by limiting GTP levels and in vivo by competition between receptors for shared G proteins, each of which could affect ligand binding (both mechanisms are discussed in more detail by Chabre et al., 2009). Similarly, our observation that the dimerization of *Frizzled* receptors appears to be mediated by specialized domains outside the TM region offers new support for the notion that the formation of stable dimers requires greater binding energies than can often be generated by the TM region alone, as also suggested elsewhere (Gurevich and Gurevich, 2008b). Interestingly, two of the *Rhodopsin*-family GPCR dimers identified here (LGR4 and LGR5) have large extracellular domains similar in structure to those of the *Frizzled* receptors. For the other *Rhodopsin*-family dimers, it is more likely that dimerization relies only on TM region contacts, as suggested here for S1P3, implying that these types of receptors, at best, interact only transiently.

Several of our observations would be explained by the rare, episodic appearance of *Rhodopsin-*family dimers during vertebrate evolution (Figure 5F): (1) that the dimers comprise a small fraction of GPCRs; (2) that they form small, closely related phylogenetic clusters; (3) that these receptors do not share ligand- or G protein selectivity outside the clusters; and (4) that closely related receptors can have different stoichiometries. The infrequency and distribution of the dimers seem to mirror a striking correlation between stoichiometry and GPCR family size also suggested by our data. Despite all appearing contemporaneously ∼1.3 billion years ago, the dimeric *Frizzled* and *Glutamate* receptor families contain only 11 and 22 members, respectively, whereas there are now >700 predominantly monomeric *Rhodopsin-*family receptors (Nordstrom et al., 2011). Similarly, having split from the *Rhodopsin* family just ∼300 million years ago (Nordstrom et al., 2011), it seems telling to us that there are >28 monomeric *Taste2* receptors and only three *Glutamate*-family *Taste1* dimers (Chandrashekar et al., 2000, Nelson et al., 2001), bearing in mind that both groups of receptors perform the same physiological function.

Why, then, are *Rhodopsin-*family GPCRs so dominant among extant vertebrates, and why are the numbers of putative dimers so low? Assuming that the root ancestor of the *Rhodopsin-*family GPCRs was monomeric, as our limited analysis of the *cAMP*-family GPCRs seems to imply, we suggest that at least three forces helped shape *Rhodopsin-*family receptor evolution. First, the functional autonomy of *Rhodopsin*-family monomers (e.g., Bayburt et al., 2007, Kuszak et al., 2009, Leitz et al., 2006, Whorton et al., 2007), allied with an intron-less mode of gene duplication that would have preserved this functionality, might have favored a classical birth-and-death mechanism of family expansion (Nei and Rooney, 2005). Second, the cylindrically arranged TM domains, which form deep pockets that bind mostly small ligands, could have allowed very fast functional diversification. Finally, dimerization might have increased the “fitness density” of receptors whose functions depend on dimerization, constraining the capacity of families of dimers, such as the *Frizzled* and *Glutamate* receptors, to diverge (Makino and Gojobori, 2006). This is because, following gene duplication, any “new” receptor would potentially interfere with the function of the “parent” receptor until the capacity for physical interactions was lost, as proposed for other receptor systems (e.g., Fraser et al., 2003, Makino and Gojobori, 2006). The paucity of *Rhodopsin-*family dimers could reflect the frequency of spontaneous gain-of-dimerization events if dimerization, per se, does not confer functionality.

## Experimental Procedures

### Quantification of HEK293T Transcriptome

cDNA was generated by reverse transcription of total mRNA harvested from HEK293T cells and sequenced using high-throughput RNA-seq (Illumina). Individual sequences were mapped and quantified using TopHat and Cufflinks software (Center for Computational Biology, Johns Hopkins University). Output fragments were then assigned as encoding GPCRs by reference to the Universal Protein Resource (www.uniprot.org).

### BRET Vector Construction

GPCR genes were cloned into the pGFP^2^ N3 (PerkinElmer), pRluc N3 (PerkinElmer), and pU (described in Felce et al., 2014) vectors for BRET experiments. Genes were amplified from cDNA generated from HEK293T cells by PCR using oligonucleotide primers binding the 5′ and 3′ sequences of either the open reading frame (ORF) or UTRs, or in two stages whereby the 5′ and 3′ halves of the ORF were amplified independently and then used as a template for generating a chimeric full-length product. All oligonucleotide primers used in this study and the cloning route adopted for each *Rhodopsin*-family GPCR gene are described in Data S1 (“R Primers” and “Non-R Primers”). In some instances, the full-length sequence of the gene of interest was synthesized directly (GeneArt, Invitrogen). cDNA encoding β_1_AR was obtained from Robert Lefkowitz (Duke University) in the form of a FLAG-*ADRB1* pcDNA3 construct (Tang et al., 1999) from Addgene (plasmid #14698). This was used as a template for full-length PCR amplification as described earlier. Candidate *cAMP*-family receptors were synthesized directly using GeneArt and amplified using primers given in Data S1 (“Non-R Primers”). All amplified GPCR genes were cloned into the N3 BRET vectors by digestion at the flanking restriction endonuclease sites indicated in Data S1 (“R Primers” and “Non-R Primers”). The 3′ terminal restriction site used in most cases was BamHI, leading to encoded proteins with a linker sequence of GDPPVAT between the C terminus of the GPCR and the N-terminal sequence of GFP^2^ or Rluc. Subsets of constructs contained linker sequences of GVPRARDPPVAT (12 receptors) or KLAVPRARDPPVAT (2 receptors), owing to differences in restriction site used; linker sequences are given in Data S1 (“R Primers” and “Non-R Primers”). The choice of linker seemed to have no bearing on observed receptor stoichiometry (Figures S4E and S4F).

FKBP-tagged CD86 and β_2_AR constructs were prepared by amplifying FKBP cDNA from the pC_4_-F_V_1E vector (Ariad) using oligonucleotide primers 5′-TAGTAGGGATCCAGGAGTGCAGGTGGAAACCATC-3′ and 5′-CTACTAGGCTCCCCCTCCAGCTTCAGCAGCTCCACGTCG-3′. The amplified product was then ligated between CD86- or β_2_AR-encoding cDNA and sequences encoding GFP^2^ or Rluc in N3 BRET constructs previously described by James et al. (2006), using a BamHI restriction site.

### Chimeric Receptor Cloning

Chimeras of the *S1PR3/LPAR1* and *FZD10*/*TAS2R19* genes were generated using multiple overlapping PCR reactions using primers given in Data S1 (“Non-R Primers”). Full details are provided in the Supplemental Experimental Procedures.

### Confocal Microscopy

Surface expression of GPCRs fused to GFP was assessed using confocal microscopy. HEK293T cells were transfected 24 hr after plating onto microscope coverslips, with 1 μg pGFP^2^-GPCR DNA per 6 × 10^5^ cells, using GeneJuice (Novagen) as per the manufacturer’s protocol. Cells were fixed using PBS/4% paraformaldehyde for 10 min. Coverslips were then mounted onto glass slides using VectaShield mounting medium (Vector Laboratories) and imaged using a Zeiss LSM-780 inverted confocal microscope under an oil-immersed 60× objective lens, with excitation laser light typically at 488 nm. Images were collected at the midpoint of the cell using a 515-nm ± 15-nm emission filter and manipulated minimally to improve the signal-to-noise ratio.

Receptors were assessed qualitatively for cellular distribution and assigned an expression category (Table S1). Protein aggregation was identified by the presence of non-uniform accumulations of GFP fusion protein with fluorescence intensities and z-plane distributions greater than that expected of protein retained in internal membranes (Zhang et al., 2005). Typically, such aggregates would be the brightest objects in the field of view. Proteins with expression profiles assigned to either category F or category G were not progressed to pRluc and pU expression, or to subsequent BRET analysis.

### Type-1 and -3 BRET Assays

Type-1 and -3 BRET assays were performed on all GPCRs with adequate surface expression according to confocal microscopy. Type-1 BRET was performed as described previously (James et al., 2006) on HEK293T cells transiently transfected with BRET pairs of the target gene. Additional details are provided in the Supplemental Experimental Procedures. BRET_eff_ and GFP:Rluc ratios were measured 24 hr post-transfection for the majority of receptors. Some GPCRs were insufficiently well expressed to give reliable data after 24 hr and so were, instead, assayed after 48 hr. GPCRs assayed after 48 hr were: AT_1_, CCR11, GPER, NPY1R, OR4D1, and PAR1. Each receptor was tested in at least three independent experiments until a sufficiently broad range of GFP:Rluc ratios was assayed.

Type-3 BRET assays were performed as described previously (Felce et al., 2014) in HEK293T cells transiently transfected with BRET pairs of the target gene along with competitor or blank pU expression vector. In all instances, a 2:1 ratio of pU:(pGFP^2^+pRluc) was used to ensure an excess of competitor over labeled protein (an equivalent expression of proteins from pGFP^2^, pRluc, and pU vectors was demonstrated previously; Felce et al., 2014), and a 12:1 pGFP^2^:pRluc ratio was used to ensure measurable levels of BRET. In most cases this was achieved by transfecting 6 × 10^5^ cells with 1 μg pU, 0.462 μg pGFP^2^, and 0.038 μg pRluc. In cases of low receptor expression, these amounts were increased to 2 μg pU, 0.924 μg pGFP^2^, and 0.076 μg pRluc. Increases in DNA were required for 5-HT_2B_, AT_1_, B_2_, CCR11, EDNRA, GPER, NPY1R, OR4D1, OXER1, and PAR1. Data were collected from a minimum of three independent experiments.

For BRET analysis of FKBP-tagged inducible dimers, cells were incubated for 45 min at room temperature in the presence of various amounts of AP20187 inducer in PBS prior to the assay. Amounts of inducer required to achieve various levels of dimerization were calculated from the relationship between inducer concentration and BRET_eff_ for CD86_FKBP_ (Figure S2B). The required concentrations were: 0% dimerization, 0 nM; 10% dimerization, 35 nM; 20% dimerization, 85 nM; 30% dimerization, 145 nM; 40%, 225 nM; 50%, 335 nM; and 100%, 5 μM. The statistical methods used to analyze the BRET data are described in the Supplemental Experimental Procedures.

### Vector Construction for SMCCCD

C-terminally SNAP-tag- and HaloTag-labeled LPA1, S1P3, β_1_AR, and α_2C_AR were expressed by subcloning the respective genes from pGFP^2^ into pHRI-SNAP-tag and pHRI-HaloTag vectors, as described previously (Latty et al., 2015), using *MluI* and *NotI* restriction sites. In both pHRI vectors, expression is under the control of the ecdysone-dependent minimal promoter so that expression is limited to ∼2,000–4,000 receptors per cell. CD86- and CD28-expressing vectors for SMCCCD were described previously (Latty et al., 2015). All receptors analyzed with SMCCCD contained a short Gly-Asp-Pro sequence between the C terminus of the receptor and the N terminus of either SNAP-tag or HaloTag.

### SMCCCD Analysis

CHO K1 cells were transfected with receptor-expressing constructs for SMCCCD analysis as described elsewhere (Latty et al., 2015). DNA ratios and post-transfection incubations required to achieve 100–1,000 HaloTag spots per cell and SnapTag:HaloTag ratios between 1:1 and 6:1 are indicated in Table S2. Receptor labeling, fixation, and data collection were performed as described previously (Latty et al., 2015).

## Author Contributions

J.H.F., S.L.L., D.K., and S.J.D. designed the experiments. J.H.F., S.L.L., S.F.L., D.K., and S.J.D. wrote the manuscript. J.H.F. and R.G.K. cloned, expressed, and collected BRET data for all GPCRs. S.R.M. cloned, expressed, and collected BRET data for most *Frizzled* and *Taste2* GPCRs. J.H.F. analyzed the BRET data. Y.L. performed and analyzed the RNA-seq profiling. J.H.F. and S.L.L. performed the microscopy experiments and analyzed the data.

## Data and Software Availability

The RNA-seq data described in this paper have the accession number GEO: GSE102461. The original experimental data are available at Mendeley Data, accession number http://dx.doi.org/10.17632/tb93gs9j29.1

## Supplemental Information

Document S1. Supplemental Experimental Procedures, Figures S1–S5, and Tables S1–S3

Data S1. Oligonucleotide Primers and Linker Sequences for Non-*Rhodopsin* Family GPCRs and Chimeras The file named “Supplementary Data” contains the RNA expression levels, in HEK 293T cells, for each of the receptors studied, along with the results of the statistical analysis of the types-1 and -3 BRET experiments, and lists of the primers used to clone the *Rhodopsin-* and non-*Rhodopsin*-family receptors.

Document S2. Article plus Supplemental Information
