# Supplementary material for: Receptor Quaternary Organization Explains G Protein-Coupled Receptor Family Structure
Source: Cell Rep. 2017 Sep 12;20(11):2654–65. doi: 10.1016/j.celrep.2017.08.072 (PMC5608970; doi:10.1016/j.celrep.2017.08.072)
Supplement: Document S1. Supplemental Experimental Procedures, Figures S1–S5, and Tables S1–S3 [file mmc1.pdf]

**Cell Reports, Volume *20***

## Supplemental Information

# Receptor Quaternary Organization Explains

# G Protein-Coupled Receptor Family Structure

**James H. Felce, Sarah L. Latty, Rachel G. Knox, Susan R. Mattick, Yuan Lui, Steven F. Lee, David Klenerman, and Simon J. Davis**

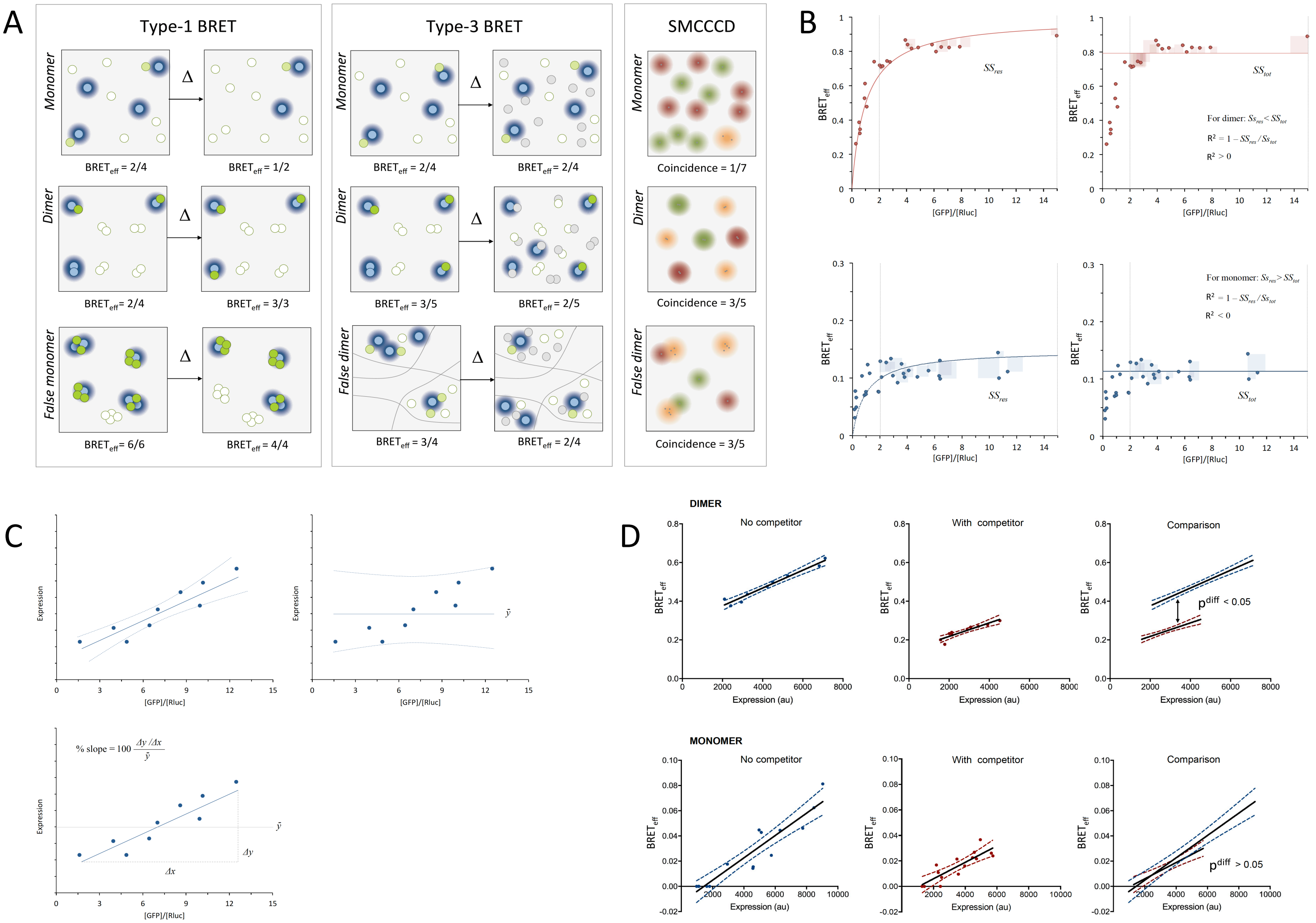
A
Type-1 BRET
Type-3 BRET
SMCCCD
Monomer
Dimer
False monomer
False dimer
Δ
BRETeff = 2/4
BRETeff = 1/2
BRETeff = 2/4
BRETeff = 3/3
BRETeff = 6/6
BRETeff = 4/4
BRETeff = 2/4
BRETeff = 2/4
BRETeff = 3/5
BRETeff = 2/5
BRETeff = 3/4
BRETeff = 2/4
Coincidence = 1/7
Coincidence = 3/5
Coincidence = 3/5
B
BRETeff
[GFP]/[Rluc]
SSres
SStot
For dimer: SSres < SStot
R² = 1 – SSres / SStot
R² > 0
For monomer: SSres > SStot
R² = 1 – SSres / SStot
R² < 0
C
Expression
[GFP]/[Rluc]
ȳ
% slope = 100 (Δy / Δx) / ȳ
Δy
Δx
D
DIMER
No competitor
With competitor
Comparison
BRETeff
Expression (au)
pdiff < 0.05
MONOMER
No competitor
With competitor
Comparison
pdiff > 0.05

**Figure S1. Explanations of Assay Principles and Statistical Analyses, Related to Figure 1.**

(A) Comparisons of outcomes and limitations of type-1 and -3 BRET assays and SMCCCD. BRET donors are represented as blue circles with halos of the RET-permissive radius; BRET acceptors as white (non-fluorescing) or green (fluorescing) circles. SMCCCD-imaged fluorophores are shown as individual color (red and green) or combined color (orange) diffraction-radius spots surrounding the tagged protein (gray). In the type-1 BRET assay, monomers exhibit no change in $BRET_{eff}$ as acceptor:donor ratio increases because the replacement of donors with acceptors does not impact on the availability of acceptors for the remaining donors (left, top). Conversely, as acceptor:donor ratio increases for dimers $BRET_{eff}$ will increase as fewer donor-donor pairs remain (left, middle). False monomer results can be produced in the case of high-order oligomers as $BRET_{eff}$ is also largely unaffected by increases in acceptor:donor ratio (left, bottom). In the type-3 BRET assay, monomers exhibit no change in $BRET_{eff}$ when untagged competitor proteins are introduced into the system (center, top), whereas $BRET_{eff}$ for dimers will decrease due to disruption of productive donor-acceptor dimers (center, middle). False dimers can be produced in the case of monomers that undergo clustering within the membrane that becomes more relaxed upon introduction of competitors. This causes a reduction in non-specific $BRET_{eff}$ due to the reduced effective concentration of tagged proteins (center, bottom). In SMCCCD, tagged proteins are detected as diffraction-limited spots with all proteins within the diffraction-limited area identified as a single spot. Proteins are tagged and imaged in two colors, allowing two or more proteins within the diffraction-limited spot (*i.e.* 'coincident' signals) to be identified. For monomers (right, top), apparent coincidence is the product only of by-chance co-localization within the diffraction-limited spots. Coincidence is higher for dimers (right, middle) because *bona fide* interaction results in up to 50% (*i.e.* 25% green-green, 25% red-red, 50% green-red) of receptors being co-localized within the diffraction-limited spots. SMCCCD cannot distinguish between genuine dimers and clusters of monomers (right, bottom), which are observed as false dimers.

(B) Graphical explanation of type-1 BRET statistical analysis. For all type-1 BRET assays, data were fitted to dimer (left) and monomer (right) models. Fits were generated only for [GFP]/[Rluc] values between 2 and 15. The dimer models fitted data to Equation 1, while the monomer model fitted to a constant $BRET_{eff}$ across all [GFP]/[Rluc] values (i.e. $BRET_{eff} = BRET_{max}$). In both cases non-linear least-squares regression was used to generate optimal fits. To determine which fit better suited the data, the $R^2$ value was determined. This compares the residual sum of squares for the dimer model fit ($SS_{res}$) to that of a flat line (*i.e.* the monomer model; $SS_{tot}$). $R^2$ is determined as $1 - SS_{res} / SS_{tot}$. For a dimer, *e.g.* CXCR4 (top), $SS_{res}$ is smaller than $SS_{tot}$, so $R^2$ is positive. For a monomer, *e.g.* CCR6 (bottom), $SS_{res}$ is larger than $SS_{tot}$, so $R^2$ is negative.

(C) Explanation of *p* and slope metrics for expression *vs* [GFP]/[Rluc] in type-1 BRET assays. The type-1 assay relies on overall expression being constant as acceptor:donor ratio increases. The probability, *p*, that total protein expression varied systematically with [GFP]/[Rluc] was tested by comparing the goodness-of-fit of a least-squares linear regression fit of the data (left, top) to that of a zero slope fit around mean expression (right, top) using a Fisher test. If the linear regression is significantly non-zero in its slope then the resulting *p* value is <0.05. *P* values for all type-1 BRET assays are provided in the Supplementary Data ("BRET Experiments"), along with mean percentage slope for all experiments. This was calculated as the slope exhibited by the linear regression fit expressed as a percentage of the mean expression value for all points (bottom); *i.e.*, if the percentage slope is 5.00, the total expression would increase 65% across the active range of 2-15 [GFP]/[Rluc].

(D) Graphical explanation of type-3 BRET statistical analysis. For all type-3 BRET assays, both datasets (*i.e.* with and without competitor) were fitted to a least-squares linear regression model (black line; dotted lines are 95% confidence limits of the fit). All points were used in the generation of the fit. The difference in the elevation of the two fits was then tested using a *t* test to determine the probability that the two fits came from samples with identical *t* distributions. A significant difference in the linear regression models resulted in $p^{diff}$ value below 0.05, whereas a $p^{diff}$ over 0.05 indicated no significant difference. The existence of a significant difference between datasets indicated the presence of dimers (*e.g.* CXCR4; top), whereas its absence suggested monomeric behavior (*e.g.* CCR6; bottom).

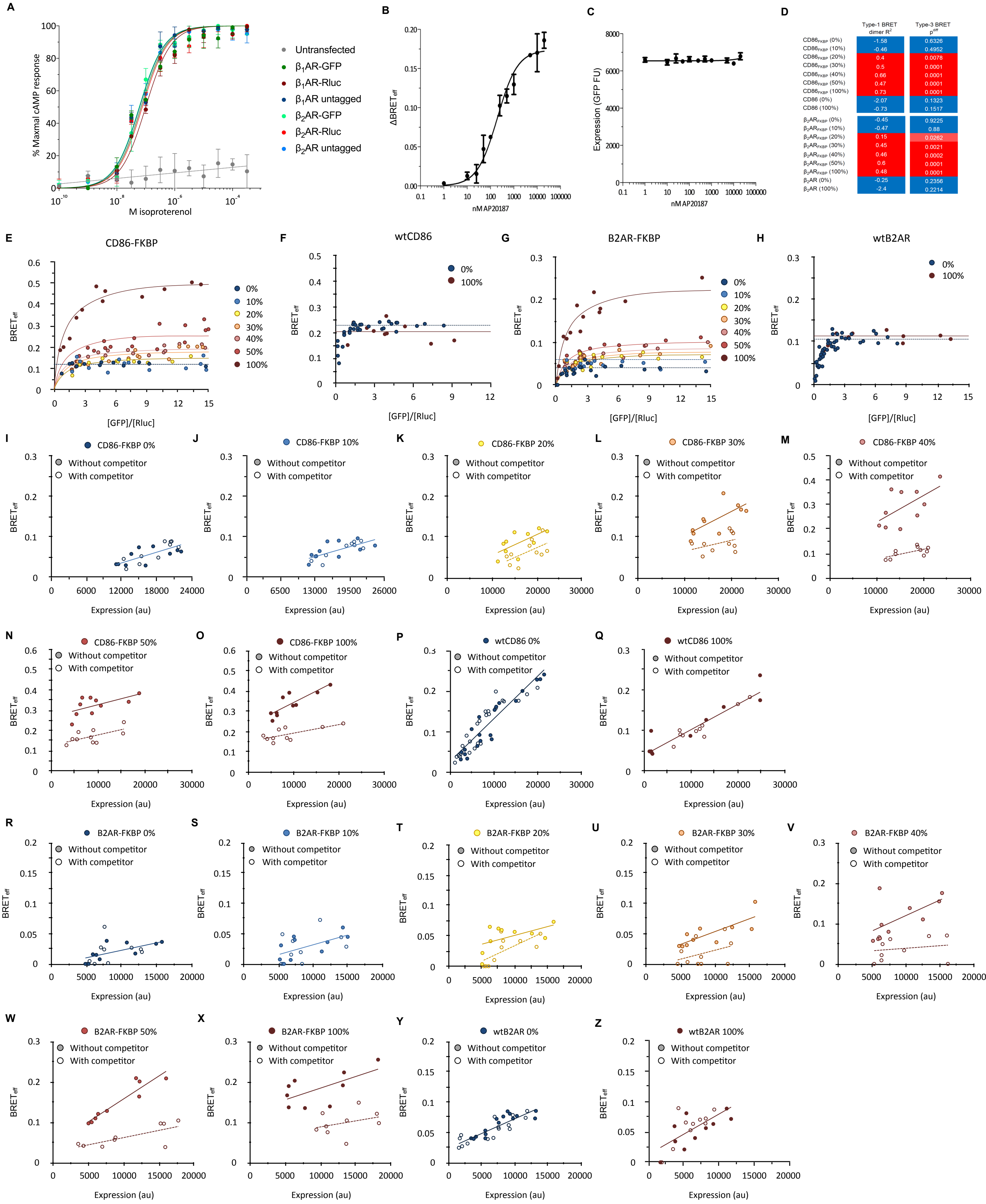
A
% Maxmal cAMP response
M isoproterenol
Untransfected
β1AR-GFP
β1AR-Rluc
β1AR untagged
β2AR-GFP
β2AR-Rluc
β2AR untagged
B
ΔBRETeff
nM AP20187
C
Expression (GFP FU)
nM AP20187
D
Type-1 BRET dimer R2
Type-3 BRET pdiff
CD86FKBP (0%) -1.58 0.6326
CD86FKBP (10%) -0.46 0.4952
CD86FKBP (20%) 0.4 0.0078
CD86FKBP (30%) 0.5 0.0001
CD86FKBP (40%) 0.66 0.0001
CD86FKBP (50%) 0.47 0.0001
CD86FKBP (100%) 0.73 0.0001
CD86 (0%) -2.07 0.1323
CD86 (100%) -0.73 0.1517
β2ARFKBP (0%) -0.45 0.9225
β2ARFKBP (10%) -0.47 0.88
β2ARFKBP (20%) 0.15 0.0262
β2ARFKBP (30%) 0.45 0.0021
β2ARFKBP (40%) 0.46 0.0002
β2ARFKBP (50%) 0.6 0.0001
β2ARFKBP (100%) 0.48 0.0001
β2AR (0%) -0.25 0.2356
β2AR (100%) -2.4 0.2214
E CD86-FKBP
F wtCD86
G B2AR-FKBP
H wtB2AR
BRETeff
[GFP]/[Rluc]
0% 10% 20% 30% 40% 50% 100%
I CD86-FKBP 0%
J CD86-FKBP 10%
K CD86-FKBP 20%
L CD86-FKBP 30%
M CD86-FKBP 40%
N CD86-FKBP 50%
O CD86-FKBP 100%
P wtCD86 0%
Q wtCD86 100%
R B2AR-FKBP 0%
S B2AR-FKBP 10%
T B2AR-FKBP 20%
U B2AR-FKBP 30%
V B2AR-FKBP 40%
W B2AR-FKBP 50%
X B2AR-FKBP 100%
Y wtB2AR 0%
Z wtB2AR 100%
Without competitor
With competitor
Expression (au)

**Figure S2 The Type-1 and Type-3 BRET Assays Readily Detect Dimers Corresponding to Only 20% of the Tagged Receptor Population, Related to Figure 1.**

(A) cAMP responses of untransfected CHO-K1 cells and CHO-K1 cells transiently transfected with untagged, GFP-, and Rluc-tagged versions of human $\beta_1$AR and $\beta_2$AR, as measured using the GloSensor cAMP assay. Data are expressed as % maximal response in each dataset, apart from the untransfected control, which are normalized to data for untagged $\beta_2$AR. Error bars indicate mean ± SE.

(B) Change in $BRET_{eff}$ between $CD86_{FKBP}GFP^2$ and $CD86_{FKBP}$Rluc in the presence of increasing concentrations of AP20187. AP20187 concentrations required to achieve various levels of dimerization were derived from these data. Error bars indicate mean ± SE.

(C) Total $CD86_{FKBP}GFP^2$ fluorescence at increasing AP20187 concentrations. Fluorescence is constant across concentrations, indicating AP20187 does not induce internalization and degradation of $CD86_{FKBP}$. The type-1 and type-3 BRET assays can detect dimerization of $CD86_{FKBP}$ and $\beta_2AR_{FKBP}$ at AP20187 concentrations sufficient to induce 20% dimerization. Error bars indicate mean ± SE.

(D) Color-coded summary of type-1 and -3 BRET assay outcomes for inducible dimers and wild-type controls at concentrations of AP20187 sufficient to induce various degrees of dimerization. Color coding is the same as in Figure 1E, ranging from monomeric (blue) to dimeric (red).

(E) Type-1 BRET data for $CD86_{FKBP}$ collected at various levels of induced dimerization. $CD86_{FKBP}$ demonstrates detectably dimeric behavior in the type-1 assay at AP20187 concentrations sufficient to induce 20% or more dimerization.

(F) Type-1 BRET data for wtCD86 collected without (0% dimerization) and with 5 μM AP20187 (100% dimerization). wtCD86 exhibits monomeric behavior even at an AP20187 concentration sufficient to induce 100% dimerization of FKBP-tagged equivalents.

(G) Type-1 BRET data for $\beta_2AR_{FKBP}$ collected at various levels of induced dimerization. $\beta_2AR_{FKBP}$ demonstrates detectably dimeric behavior in the type-1 assay at AP20187 concentrations sufficient to induce 20% or more dimerization.

(H) Type-1 BRET data for wt$\beta_2$AR collected without (0% dimerization) and with 5 μM AP20187 (100% dimerization). wt$\beta_2$AR exhibits monomeric behavior even at an AP20187 concentration sufficient to induce 100% dimerization of FKBP-tagged equivalents.

(I) Type-3 BRET data for $CD86_{FKBP}$ without (0% dimerization) AP20187. Data are consistent with monomeric behavior.

(J) Type-3 BRET data for $CD86_{FKBP}$ in the presence of 35 nM (10% dimerization) AP20187. Data are consistent with monomeric behavior.

(K) Type-3 BRET data for $CD86_{FKBP}$ in the presence of 85 nM (20% dimerization) AP20187. Data are consistent with dimeric behavior.

(L) Type-3 BRET data for $CD86_{FKBP}$ in the presence of 145 nM (30% dimerization) AP20187. Data are consistent with dimeric behavior.

(M) Type-3 BRET data for $CD86_{FKBP}$ in the presence of 225 nM (40% dimerization) AP20187. Data are consistent with dimeric behavior.

(N) Type-3 BRET data for $CD86_{FKBP}$ in the presence of 335 nM (50% dimerization) AP20187. Data are consistent with dimeric behavior.

(O) Type-3 BRET data for $CD86_{FKBP}$ in the presence of 5 μM (100% dimerization) AP20187. Data are consistent with dimeric behavior.

(P) Type-3 BRET data for wtCD86 without (0% dimerization) AP20187. Data are consistent with monomeric behavior.

(Q) Type-3 BRET data for wtCD86 in the presence of 5 μM (100% dimerization) AP20187. Data are consistent with monomeric behavior.

(R) Type-3 BRET data for $\beta_2AR_{FKBP}$ without (0% dimerization) AP20187. Data are consistent with monomeric behavior.

(S) Type-3 BRET data for $\beta_2AR_{FKBP}$ in the presence of 35 nM (10% dimerization) AP20187. Data are consistent with monomeric behavior.

(T) Type-3 BRET data for $\beta_2AR_{FKBP}$ in the presence of 85 nM (20% dimerization) AP20187. Data are consistent with dimeric behavior.

(U) Type-3 BRET data for $\beta_2AR_{FKBP}$ in the presence of 145 nM (30% dimerization) AP20187. Data are consistent with dimeric behavior.

(V) Type-3 BRET data for $\beta_2AR_{FKBP}$ in the presence of 225 nM (40% dimerization) AP20187. Data are consistent with dimeric behavior.

(W) Type-3 BRET data for $\beta_2AR_{FKBP}$ in the presence of 335 nM (50% dimerization) AP20187. Data are consistent with dimeric behavior.

(X) Type-3 BRET data for $\beta_2AR_{FKBP}$ in the presence of 5 μM (100% dimerization) AP20187. Data are consistent with dimeric behavior.

(Y) Type-3 BRET data for wt$\beta_2$AR without (0% dimerization) AP20187. Data are consistent with monomeric behavior.

(Z) Type-3 BRET data for wt$\beta_2$AR in the presence of 5 μM (100% dimerization) AP20187. Data are consistent with monomeric behavior.

See also Supplementary Data (“BRET Experiments”).

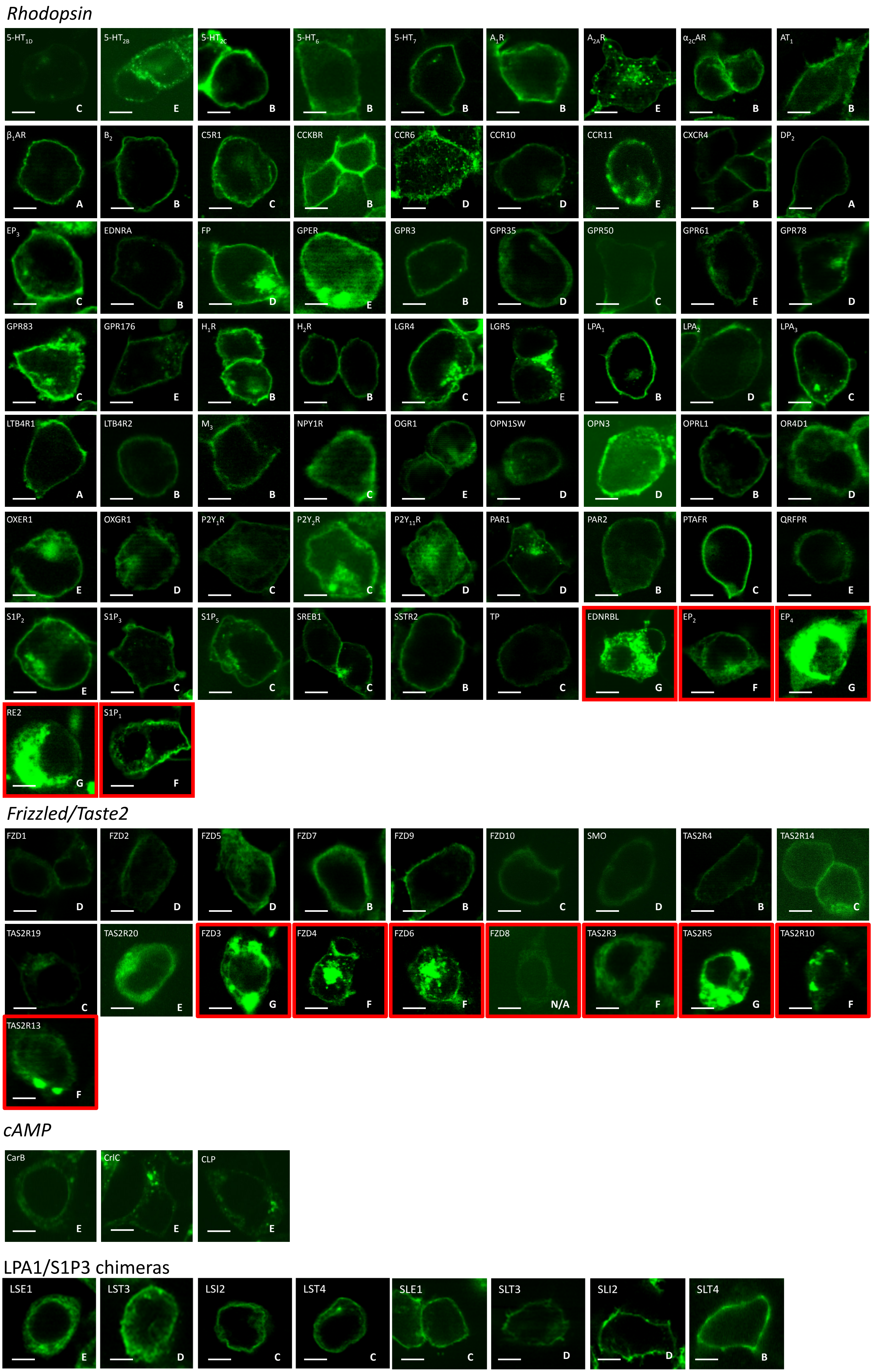
Rhodopsin
5-HT1D C
5-HT2B E
5-HT2C B
5-HT6 B
5-HT7 B
A1R B
A2AR E
α2CAR B
AT1 B
β1AR A
B2 B
C5R1 C
CCKBR B
CCR6 D
CCR10 D
CCR11 E
CXCR4 B
DP2 A
EP3 C
EDNRA B
FP D
GPER E
GPR3 B
GPR35 D
GPR50 C
GPR61 E
GPR78 D
GPR83 C
GPR176 E
H1R B
H2R B
LGR4 C
LGR5 E
LPA1 B
LPA2 D
LPA3 C
LTB4R1 A
LTB4R2 B
M3 B
NPY1R C
OGR1 E
OPN1SW D
OPN3 D
OPRL1 B
OR4D1 D
OXER1 E
OXGR1 D
P2Y1R C
P2Y2R C
P2Y11R D
PAR1 D
PAR2 B
PTAFR C
QRFPR E
S1P2 E
S1P3 C
S1P5 C
SREB1 C
SSTR2 B
TP C
EDNRBL G
EP2 F
EP4 G
RE2 G
S1P1 F
Frizzled/Taste2
FZD1 D
FZD2 D
FZD5 D
FZD7 B
FZD9 B
FZD10 C
SMO D
TAS2R4 B
TAS2R14 C
TAS2R19 C
TAS2R20 E
FZD3 G
FZD4 F
FZD6 F
FZD8 N/A
TAS2R3 F
TAS2R5 G
TAS2R10 F
TAS2R13 F
cAMP
CarB E
CrlC E
CLP E
LPA1/S1P3 chimeras
LSE1 E
LST3 D
LSI2 C
LST4 C
SLE1 C
SLT3 D
SLI2 D
SLT4 B

**Figure S3 Representative Confocal Microscopy Images of GPCR-GFP Constructs Expressed in HEK293T Cells, Related to Figure 1.**

Receptors were placed into categories A-G (Table S1) based on their subcellular localization and degree of observable GFP aggregation. Receptors identified as categories F and G (red borders) were not studied. FZD8 expressed too weakly for reliable assessment of localization, and was also not studied. Scale bars are 5 µm.

See also Table S1.

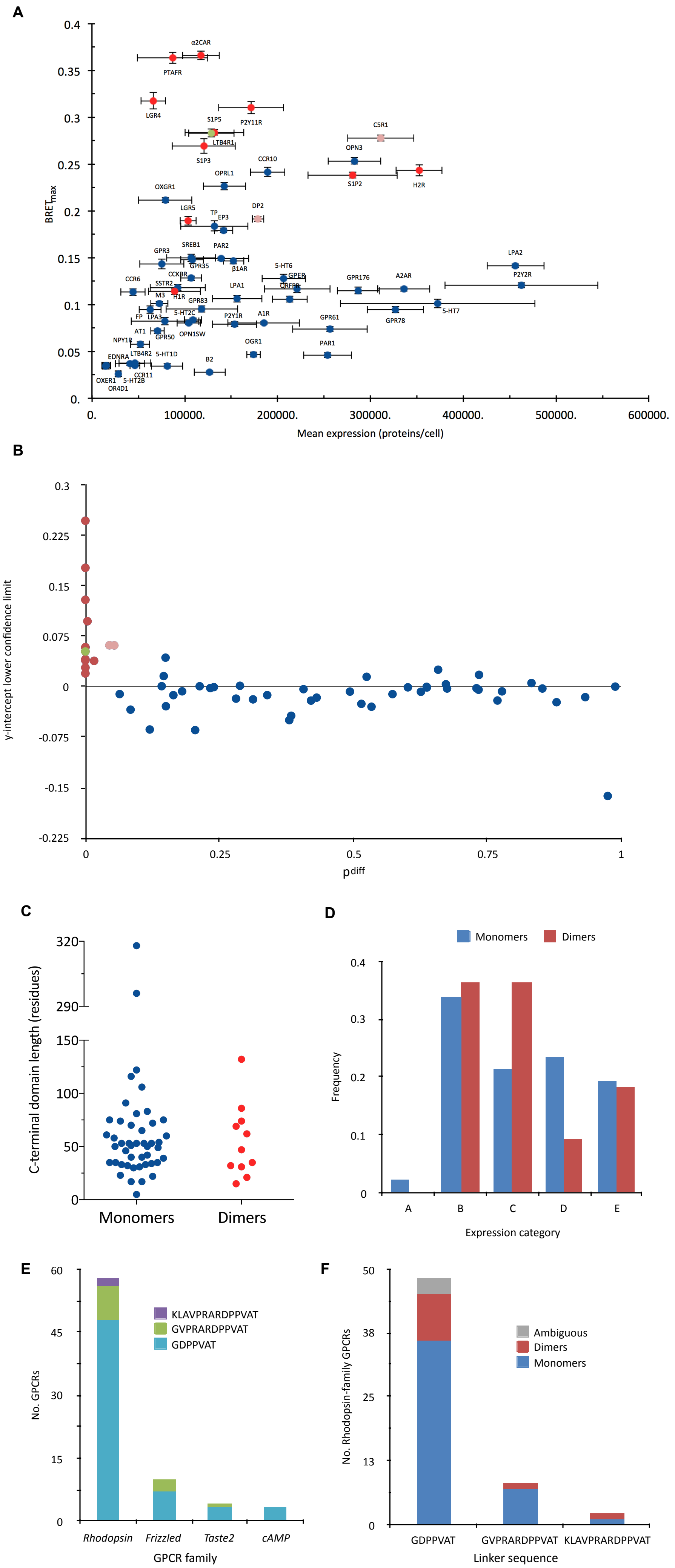
A
BRET$_{max}$
Mean expression (proteins/cell)
0.4
0.35
0.3
0.25
0.2
0.15
0.1
0.05
0.
0.
100000.
200000.
300000.
400000.
500000.
600000.
α2CAR
PTAFR
LGR4
P2Y11R
S1P5
LTB4R1
S1P3
C5R1
OPN3
CCR10
S1P2
H2R
OPRL1
OXGR1
LGR5
DP2
TP
EP3
SREB1
PAR2
GPR3
GPR35
β1AR
5-HT6
LPA2
P2Y2R
CCKBR
GPER
GPR176
A2AR
CCR6
SSTR2
M3
H1R
GPR83
LPA1
5-HT7
GPR78
FP
LPA3
5-HT2C
P2Y1R
A1R
GPR61
AT1
GPR50
OPN1SW
NPY1R
OGR1
PAR1
EDNRA
LTB4R2
5-HT1D
B2
OXER1
CCR11
5-HT2B
OR4D1
B
y-intercept lower confidence limit
p$^{diff}$
0.3
0.225
0.15
0.075
0
-0.075
-0.15
-0.225
0
0.25
0.5
0.75
1
C
C-terminal domain length (residues)
320
290
150
100
50
0
Monomers
Dimers
D
Monomers
Dimers
Frequency
Expression category
0.4
0.3
0.2
0.1
0
A
B
C
D
E
E
No. GPCRs
GPCR family
60
45
30
15
0
KLAVPRARDPPVAT
GVPRARDPPVAT
GDPPVAT
Rhodopsin
Frizzled
Taste2
cAMP
F
No. Rhodopsin-family GPCRs
Linker sequence
50
38
25
13
0
Ambiguous
Dimers
Monomers
GDPPVAT
GVPRARDPPVAT
KLAVPRARDPPVAT

**Figure S4 BRET$_{max}$, *y*-Intercept, C-Terminal Domain Length, and Expression Category for the *Rhodopsin*-Family GPCRs studied, Related to Figure 1.**

(A) Relationship between BRET$_{max}$ and expression level for HEK293T cell-expressed *Rhodopsin*-family GPCRs. BRET$_{max}$ values for monomers are generally lower than those for dimers at similar expression levels. Monomeric receptors are shown in blue; dimers in red. The ambiguous cases C5R1 and DP2 are shown in pink. LTB4R1 is shown in green. Error bars indicate SEM for each parameter. CXCR4 is not shown for clarity. Absolute values are given in the Supplementary Data ("BRET Experiments").

(B) The *y*-intercept values obtained using type-3 BRET in the absence of competitor. Receptors behaving as monomers are shown in blue, dimers in red. C5R1 and DP2 are shown in pink, LTB4R1 in green. All *Rhodopsin*-family dimers identified in this study yielded a *y*-intercept value with a lower 95% confidence limit that is above zero, as did the ambiguous cases C5R1, DP2, and LTB4R1. Most of the monomeric receptors had *y*-intercept values that are not significantly non-zero, although a small number have lower 95% confidence limits greater than zero. Absolute values are given in the Supplementary Data ("BRET Experiments").

(C) C-terminal domain lengths of receptors in the monomer and dimer populations. The lack of significant difference between the two populations suggests that the observed monomers are genuine and are not the mis-assignment of dimers with large C-terminal domains that preclude efficient energy transfer.

(D) Expression profiles of monomers and dimers. The two profiles are highly similar, which indicates that the dimers are not artefacts of intracellular retention. C5R1, DP2, and LTB4R1 are not included. Expression categories correspond to those described in Table S1.

(E) Number of GPCRs in each studied family using the specified linker sequences between the receptor C terminus and the N terminus of GFP/Rluc. Linkers are provided for each receptor in the Supplementary Data ("R Primers" and "Non-R Primers").

(F) Number of *Rhodopsin*-family GPCRs studied using each linker sequence exhibiting each stoichiometric state.

See also Supplementary Data ("R Primers" and "Non-R Primers"), Table S1.

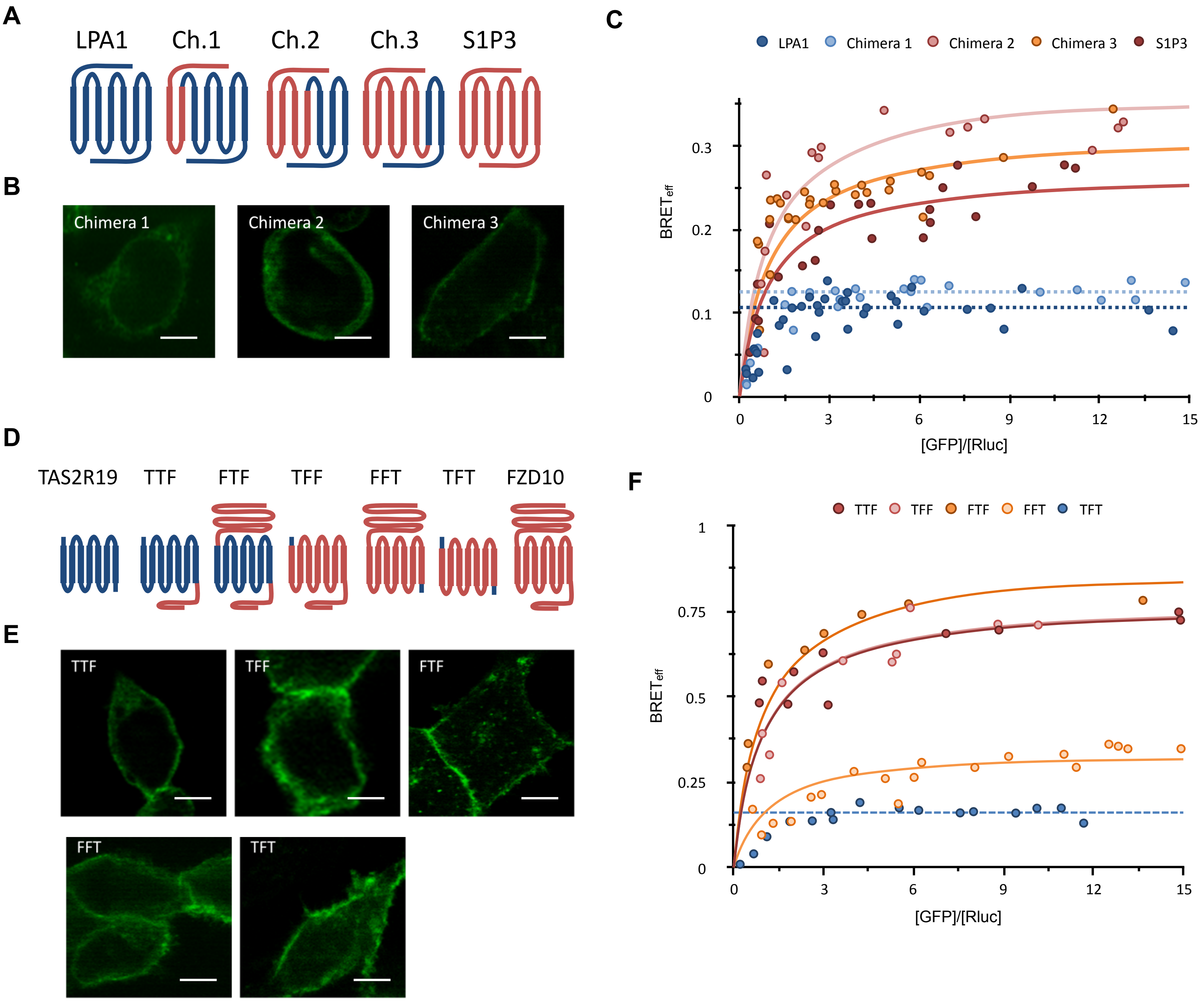
A
LPA1
Ch.1
Ch.2
Ch.3
S1P3
B
Chimera 1
Chimera 2
Chimera 3
C
LPA1
Chimera 1
Chimera 2
Chimera 3
S1P3
BRETeff
[GFP]/[Rluc]
D
TAS2R19
TTF
FTF
TFF
FFT
TFT
FZD10
E
TTF
TFF
FTF
FFT
TFT
F
TTF
TFF
FTF
FFT
TFT
BRETeff
[GFP]/[Rluc]

**Figure S5 Analysis of Chimeric Receptors Reveals Different Mechanisms of Dimerization between *Rhodopsin*- and *Frizzled*-Family Receptors, Related to Figures 3 & 4.**
(A) Schematic representations of the LPA1 (blue) and S1P3 (red) composition of each construct.
(B) Representative confocal microscopy images of HEK293T cells expressing the three LPA1/S1P3 chimeras from the pGFP$^2$ vector. All three constructs exhibit increased intracellular retention compared to the parent genes, but not aggregation and so all were suitable for use in BRET. Scale bars are 5 μm.
(C) Type-1 BRET analysis of three LPA1/S1P3 chimeras as well as the parent receptors (for comparison). For chimera 1, $BRET_{eff}$ is independent of [GFP]/[Rluc], indicative of monomeric behavior. Fits of LPA1 and chimera 1 data to a constant model are shown as broken lines. Chimeras 2 and 3 exhibit hyperbolic dependences of $BRET_{eff}$ on [GFP]/[Rluc] that fit well to a dimer model (solid lines). This indicates that S1P3 dimerization is dependent on motifs between EL1 and TM4.
(D) Schematic representations of chimeras of TAS2R19 (blue) and FZD10 (red). Chimeras were given a three-letter designation based on their composition, in which F and T denote FZD10 and TAS2R19 components, and the first, second, and third letters indicate the origin of the N-terminal domain, TM region, and C-terminal domain, respectively. Of the six possible combinations, only FTT failed to express sufficiently for BRET analysis.
(E) Representative confocal microscopy images of HEK293T cells expressing the 5 successfully expressed TAS2R19/FZD10 chimeras from the pGFP$^2$ vector. Aggregation was not apparent in any case and so all were suitable for use in BRET. Scale bars are 5 μm.
(F) Type-1 BRET analysis of TAS2R19/FZD10 chimeras indicates a role in dimerization of both the FZD10 N- and C-terminal domains. All chimeras containing either the FZD10 N- or C-terminal domains (TTF, TFF, FTF, and FFT) exhibited $BRET_{eff}$ dependence on [GFP]/[Rluc] in the manner predicted for a dimer. Replacement of the N- and C-terminal domains of FZD10 with those of TAS2R19 (chimera TFT) resulted in $BRET_{eff}$ being independent of [GFP]/[Rluc], indicating monomeric behavior. This suggests that the FZD10 TM region does not possess any inherent dimerization ability, in contrast to S1P3.
See also Supplementary Data ("BRET Experiments").

**Table S1. Qualitative Categories of GPCR Expression Based on Cellular Localization and Degree of Receptor Aggregation.**

| Category | Definition |
|---|---|
| A | Protein entirely in plasma membrane; almost no visible protein in internal membranes; no aggregation. |
| B | Protein almost entirely in plasma membrane; small amounts in internal membranes; no aggregation. |
| C | Majority of protein in plasma membrane; moderate amounts in internal membranes; no aggregation. |
| D | Some protein in plasma membrane; large amounts in internal membranes; no aggregation. |
| E | Some protein in plasma membrane; large amounts in internal membranes; small amounts of aggregation in some cells. |
| F | Some protein in plasma membrane; large amounts in internal membranes; small amounts of aggregation in most cells. |
| G | Little or no protein in plasma membrane or internal membranes; large amounts of aggregation in most cells. |

**Table S2. Transfection Conditions, HaloTag Spot Density, and Coincidence Values for Controls and GPCRs Analyzed Using SMCCCD in CHO K1 Cells.**

| Protein | HALO-tagged Construct (μg) | SNAP-tagged Construct (μg) | Post-transfection incubation (hours) | Cells imaged | No. experimental repeats | Mean HaloTag spots/cell ± SD | Mean % coincidence ± SEM | *p*-value of difference from CD86 |
|---|---|---|---|---|---|---|---|---|
| CD86 | 0.975 | 0.175 | 20 | 10 | 4 | 311±138 | 9.7±1.5 | - |
| CD28 | 0.975 | 0.175 | 48 | 8 | 3 | 288±85 | 28.2±3.7 | 0.0001 |
| LPA1 | 0.975 | 0.08 | 24 | 9 | 3 | 312±194 | 13.8±2.6 | 0.173 |
| S1P3 | 0.975 | 0.08 | 20 | 9 | 3 | 422±150 | 22.9±3.4 | 0.002 |
| $\beta_1$AR | 0.975 | 0.08 | 15 | 8 | 3 | 299±88 | 11.7±1.4 | 0.366 |
| $\alpha_{2C}$AR | 0.975 | 0.08 | 20 | 15 | 4 | 385±114 | 14.8±1.3 | 0.017 |

**Table S3. Published Reports of Homo-Oligomerization of the *Rhodopsin*-Family GPCRs Investigated in this Study.**

Twenty-six of the 60 receptors investigated have previously been reported to be homodimers in studies using a variety of approaches. GPCRs found here to be dimeric using type-1 and -3 BRET assays are underlined. Only reports of homo-oligomerization are included in this table; studies of heteromeric interactions are not shown. In most cases only one study per technique per receptor is cited.

| Receptor | Technique used to support homo-oligomerization |
|---|---|
| 5-$HT_{1D}$ | Co-IP (Salim et al., 2002), Western blotting (Lee et al., 2003) |
| 5-$HT_{2C}$ | Cysteine crosslinking (Mancia et al., 2008), Co-IP (Herrick-Davis et al., 2006), BiFC (Herrick-Davis et al., 2012), FCS (Herrick-Davis et al., 2012), FRET (Herrick-Davis et al., 2005; Herrick-Davis et al., 2006), Radioligand binding (Herrick-Davis et al., 2005; Herrick-Davis et al., 2006) |
| $A_{2A}R$ | BRET (Gandia et al., 2008), BiFC (Gandia et al., 2008; Vidi et al., 2008), FRET (Lukasiewicz et al., 2007) |
| <u>$\alpha_{2C}AR$</u> | BRET (Small et al., 2006), Co-IP (Small et al., 2006) |
| $AT_1$ | BRET (Hansen et al., 2004), Western blotting (AbdAlla et al., 2001; AbdAlla et al., 2004) |
| $\beta_1AR$ | BRET (Mercier et al., 2003), FRAP (Dorsch et al., 2009), X-ray crystallography (Huang et al., 2013), Single-molecule microscopy (Calebiro et al., 2013) |
| $B_2$ | Co-IP (Michineau et al., 2006), Western blotting (Michineau et al., 2006) |
| C5R1 | FRET (Floyd et al., 2003) |
| CCKBR | BRET (Cheng et al., 2003) |
| <u>CXCR4</u> | BiFC (Hammad et al., 2010), BRET (Babcock et al., 2003; Percherancier et al., 2005), Bivalent ligand crosslinking (Tanaka et al., 2010), X-ray crystallography (Wu et al., 2010) |
| EDNRA | Size-exclusion chromatography (Lee et al., 2012), FRET (Evans and Walker, 2008) |
| GPR50 | Co-IP (Levoye et al., 2006) |
| <u>$H_1R$</u> | Co-IP (Carrillo et al., 2003), FRET (Carrillo et al., 2003) |
| <u>$H_2R$</u> | Western blotting (Fukushima et al., 1997) |
| $LPA_1$ | β-galactosidase complementation (Zaslavsky et al., 2006) |
| $LPA_2$ | β-galactosidase complementation (Zaslavsky et al., 2006) |
| $LPA_3$ | β-galactosidase complementation (Zaslavsky et al., 2006) |
| LTB4R1 | Cysteine crosslinking (Baneres and Parello, 2003), Radioligand binding (Baneres and Parello, 2003), Cooperative ligand binding (Damian et al., 2008; Mesnier and Baneres, 2004) |
| $M_3$ | BRET (Nemoto and Toh, 2005) |
| NPY1R | FRET (Dinger et al., 2003) |
| OGR1 | β-galactosidase complementation (Zaslavsky et al., 2006) |
| $P2Y_2R$ | BRET (Suzuki et al., 2013), FRET (Kotevic et al., 2005) |
| <u>$S1P_2$</u> | β-galactosidase complementation (Zaslavsky et al., 2006), Co-IP (Van Brocklyn et al., 2002) |
| <u>$S1P_3$</u> | β-galactosidase complementation (Zaslavsky et al., 2006), Co-IP (Van Brocklyn et al., 2002) |
| SSTR2 | FRET (Grant et al., 2004), Western blotting (Grant et al., 2004) |
| TP | Co-IP (Laroche et al., 2005), ELISA (Laroche et al., 2005) |

## SUPPLEMENTARY EXPERIMENTAL PROCEDURES

### Cloning of chimeric GPCR constructs

Chimeras of the *S1PR3* and *LPAR1* genes were generated using multiple overlapping PCR reactions that in turn amplified the relevant segments of each gene and then extended them to produce the final construct. Domain boundaries for each gene were identified using the TMHMM v2.0 software from the Center for Biological Sequence Analysis, Technical University of Denmark. Oligonucleotide primers were designed to be complementary to the domain boundaries within the chimeras (sequences are given in Supplementary Data: "Non-R Primers"), *e.g.* S3-EL1, L1-TM3 is complementary to the final 15 nucleotides of the segment of *S1PR3* encoding EL1, immediately followed by a sequence complementary to the first 15 nucleotides of the segment of *LPAR1* encoding TM3. Individual fragments of each gene were amplified with the relevant combination of primers (*e.g.* TM3 of *LPAR1* was amplified using the S3-EL1, L1-TM3 F and L1-TM3, S3-IL2 R primers), and then combined by chimeric PCR in various combinations to generate full-length sequences (*e.g.* the *SLT3* chimera was generated by combining the gene fragments encoding *S1PR3* N terminus-EL1, *LPAR1* TM3, and *S1PR3* IL2-C terminus). Final PCR products were then inserted into the $pGFP^2$ vector using restriction digests of the *MluI* and *BamHI* sites, and their sequences confirmed using reversible terminator base sequencing. All constructs were then subcloned into the pRluc vector using the same restriction sites. All S1P3/LPA1 chimeras contained the receptor-GFP/Rluc linker sequence GDPPVAT.

Chimeras of the genes *FZD10* and *TAS2R19* were generated using multiple overlapping PCR reactions in the same manner as the S1P3/LPA1 chimeras. Oligonucleotide primers are given in the Supplementary Data: "Non-R Primers", and are named analogously to the *S1PR3/LPAR1* primers (*e.g.* F10-N, T19-TM is complementary to the first 15 nucleotides of *FZD10* and the first 15 nucleotides encoding the TM region in *TAS2R19*). Individual domain-encoding fragments were amplified individually and then combined in chimeric PCR reactions as described for the *S1PR3/LPAR1* constructs. The final PCR product was inserted into the $pGFP^2$ vector using restriction digests of the *MluI* and *BamHI* sites, and its sequence confirmed. All constructs were then subcloned into the pRluc vector using the same restriction sites, giving the receptor-GFP/Rluc linker sequence GDPPVAT.

### Quantitative flow cytometric analysis

Absolute receptor numbers were quantified using flow cytometric analysis of the GFP-tagged receptor variants. HEK293T cells were transiently transfected with 1 μg $pGFP^2$-GPCR vector per $6 \times 10^5$ cells using GeneJuice® (Novagen) in the same manner as for the type-1 BRET assay, and incubated for an equivalent length of time as the type-1 BRET assay (*i.e.* 24h for the majority of receptors, 48h for six exceptions). Transfected cells were analyzed by flow cytometry for GFP expression and their respective fluorescence converted into absolute protein numbers by reference to calibrated Quantibrite™ (BD Biosciences) beads. Data were collected for a total of $5 \times 10^4$ HEK293T cells for each GPCR-GFP fusion protein in each experiment, and viable single cells were gated-for using forward scatter, side scatter, and pulse width. GFP-positive cells were selected using a two-dimensional FL1 *vs* FL2 gate to prevent artefacts arising from cellular autofluorescence, and the geometric mean of GFP fluorescence determined for the GFP-positive population.

GFP fluorescence was converted into absolute receptor numbers by reference to a standard curve of GFP fluorescence *vs* surface protein expression generated for each experiment using HEK293T cells expressing a human CD2-GFP fusion protein labeled with phycoerythrin (PE)-conjugated mouse anti-human CD2 antibody (eBioscience 12-0029). Labeling was performed at an antibody concentration of 100 μg/ml to ensure saturating, monovalent binding to CD2. PE-GFP compensation was performed using unlabeled cells expressing CD2-GFP as a GFP-only control, and cells expressing CD2-Rluc labeled with PE-anti-CD2 as a PE-only control. PE fluorescence on the labeled CD2-GFP cells was then converted to antigen-binding events by reference to calibrated Quantibrite™ (BD Biosciences) PE beads as per the manufacturer's instructions. Absolute protein numbers were determined in this manner for each receptor in three independent replicate experiments.

**BRET assays**

6-well plates were seeded with $6 \times 10^5$ HEK293T cells in 2 ml DMEM (+ 10% FCS, 2 mM L-glutamine) to ensure ~80% confluence after 24h. BRET constructs were co-transfected as BRET pairs consisting of both GFP- and Rluc-tagged proteins using GeneJuice® (Novagen) as per the manufacturer's instructions. DNA was always used at a final concentration of 0.05 μg/μl and total volume of 20 μl per well (*i.e.* 1 μg). In the type-1 BRET assay $pGFP^2$:pRluc ratios ranging from 1:2 to 66:1 were used as this gave the most useful spread of GFP:Rluc protein values. This was achieved by varying the volume of $pGFP^2$ and pRluc solutions in the final 20 μl volume; for example, a 3:1 $pGFP^2$:pRluc ratio would constitute 15 μl $pGFP^2$ (*i.e.* 0.75 μg) and 5 μl pRluc (*i.e.* 0.25 μg). In the type-3 assay, a 2:1 ratio of pU:($pGFP^2$+pRluc) was used to ensure an excess of competitor over labeled proteins, and a 12:1 $pGFP^2$:pRluc ratio was used to ensure measurable levels of BRET. In the majority of cases this was achieved using a transfection strategy of 1 μg pU, 0.462 μg $pGFP^2$, and 0.038 μg pRluc per well of $6 \times 10^5$ cells, however in cases of low receptor expression these amounts were increased to 2 μg pU, 0.924 μg $pGFP^2$, and 0.076 μg pRluc per well. Such an increase in DNA was required for 5-$HT_{2B}$, $AT_1$, $B_2$, CCR11, EDNRA, GPER, NPY1R, OR4D1, OXER1, and PAR1. 1 μg of expression vector for a soluble, fused form of Rluc-GFP (sGFP-Luc; PerkinElmer) was always used to transfect one well as the positive BRET control, and a negative control of mock-transfected cells (*i.e.* no DNA) was also always included.

Transfected HEK293T cells were collected from wells 24h after initial transfection and resuspended in PBS to a density of ~$1.5 \times 10^6$ cells/ml. $BRET_{eff}$ ratios were obtained by adding DeepBlueC (PerkinElmer) to a final concentration of 10 μM in 100 μl cell suspension in a 96-well OptiPlate (PerkinElmer) and collecting light emission in the BRET-A (410 ± 40 nm) and BRET-B (515 ± 15 nm) wavelengths. Collection for each wavelength was performed 3 times integrated over 1 second on a Fusion® Microplate Analyzer (PerkinElmer), thus giving a BRET-A and BRET-B value for each transfection. $BRET_{eff}$ values were calculated as BRET-B/BRET-A after background subtraction and correction for luciferase expression. $BRET_{eff}$ is normalized as a function of the $BRET_{eff}$ measured for the sGFP-Luc positive control, which was assigned a constant value of 1.

GFP expression was determined by exciting 100 μl of cells (in a new well) at 425 ± 25 nm and measuring emission at 515 ± 15 nm three times over 1 second, thereby giving the total fluorescence units (RFU). The same cells were then incubated for 2 min with 10 μM coelenterazine-*h* (final concentration) before measuring total emission 3 times over 1 second to give the total luminescence units (RLU). Acceptor/donor ratio was calculated using the

RLU/RFU value obtained from cell expressing the sGFP-Luc positive control, since the acceptor:donor ratio for this construct is one.

**Analysis of BRET data**

Analysis of all BRET data was performed using the Prism5 (GraphPad) software.

**(i) Type-1 assays**

Type-1 assay data were fitted to models of both dimeric (Equation 1) and monomeric (constant) behavior using the nonlinear least-squares regression function. The lower and upper range limits of [GFP]/[Rluc] values included in the analysis were 2 and 15, respectively, since 2 is the value at which $BRET_{eff}$ becomes independent of acceptor:donor ratio as confirmed with numerous controls (James et al., 2006), while 15 is the point at which the dimer model curve has flatted sufficiently to make it indistinguishable from a flat line within the typical error of the experiment. Restricting analysis within these thresholds therefore allows the most sensitive discrimination between monomer and dimer models. $BRET_{eff}$ data for [GFP]/[Rluc] values between 0 and 2 were included in plots for completeness, but were not used in either curve fitting or statistical analysis. The coefficient of determination ($R^2$) for the monomer fit is always zero as calculated $BRET_{eff}$ is a constant value. An $R^2$ value of less than zero for the dimer model indicates that it has a worse goodness-of-fit to the data than the monomer model, whereas an $R^2$ greater than zero indicates a better goodness-of-fit. Examples of monomer and dimer statistical outcomes are given in Figure S1.

$$\frac{\mathrm{BRET_{eff}}}{\mathrm{BRET_{max}}} = 1 - \frac{1}{(1+f)^{n-1}} \qquad \text{(Equation 1)}$$

Where:

$f$ = acceptor:donor ratio

$n$=stoichiometry

$BRET_{max}$ = maximal $BRET_{eff}$ achievable in each experiment

The relative expression level for all BRET assays was calculated as the combined expression of the GFP- and Rluc-tagged proteins expressed as a function of Rluc emission. This was achieved by converting GFP fluorescence units (FLU) into arbitrary Rluc luminescence (RLU) units using the GFP:Rluc ratio (*f*), whereupon total relative expression was calculated as the sum of GFP and Rluc luminescence units (Equation 2). In effect, this calculates the total RLU that would be expected if all GFP molecules in the sample were replaced with Rluc.

$$\mathrm{Expression_{total}^{RLU}} = \mathrm{GFP^{RLU}} + \mathrm{Rluc^{RLU}} \qquad \text{(Equation 2)}$$

$$\mathrm{GFP^{RLU}} = f\mathrm{Rluc^{RLU}}$$

Where:

$f$ = acceptor:donor ratio

Values for $f$ were determined individually for each sample by reference to the FLU and RLU values of the GFP-Rluc positive control, which has an inherent GFP:Rluc of 1 (Equation 3).

$$\frac{[\mathrm{GFP}]}{[\mathrm{Rluc}]} = \frac{\mathrm{RLU/FLU}}{\mathrm{RLU^{positive}/FLU^{positive}}} \qquad \text{(Equation 3)}$$

Total expression *vs* [GFP]/[Rluc] was plotted and fitted to a linear least-squares regression, then assessed for deviation from a non-zero slope using a Fisher F test ($p<0.05$ indicates a significant deviation from zero). The $p$ values are given in the Supplementary Data: "BRET Experiments", along with mean percentage slope as explained in Figure S1C.

**(ii) Type-3 assays**

All type-3 assay data were fitted using the linear least-squares regression function for total expression (from Equation 2) *vs* $BRET_{eff}$, generating separate fits for the data collected in the presence and absence of competitor. Goodness-of-fit was confirmed using the $R^2$ statistic and found to be high in all cases. $p^{diff}$ values were determined as the probability that the two datasets were from populations with identical $t$ distributions. The larger the $p^{diff}$ value, the lower the probability of difference between datasets, and hence dimers were defined as those receptors yielding a significant difference between the two conditions (Figure S1D).

**cAMP assay**

Gs-coupled signaling by tagged and untagged $\beta_1$AR and $\beta_2$AR was assessed using the GloSensor cAMP Assay (Promega) as per the manufacturer's instructions. This was performed in CHO K1 cells in order to avoid the complication of natively expressed human receptors. CHO K1 cells were plated at $1 \times 10^5$ cells/well in 96-well plates. 24 h post plating, cells were transfected with 100 ng/well each of the expression vector for the receptor of interest and the pGloSensor-22F cAMP plasmid using GeneJuice (Novagen), as per the manufacturer's instructions. pGloSensor-22F encodes a variant of firefly luciferase containing a cAMP-binding moiety, binding of which to cAMP induces a conformational change leading to a 100 fold increase in light emission. 24 h after transfection, cells were equilibrated for 2 h with GloSensor cAMP reagent as per the manufacturer's instructions. Cells were incubated at room temperature with various concentrations of the partial agonist isoproterenol (Sigma Aldrich) for 5 min before light output was measured using a Fusion Microplate Analyzer (PerkinElmer).

All cAMP assay data were corrected for background luciferase emission by subtraction of values from cells in the absence of agonist. Changes in light emission were normalized to a percentage maximal response for each read. Mean percentage response and SEM values were calculated for each agonist concentration and then fitted to a nonlinear regression normalized dose-response stimulation model: response ($x$) = $100/(1 + 10^{\log(EC50-x)})$.

## SUPPLEMENTAL REFERENCES

AbdAlla, S., Lother, H., el Massiery, A., and Quitterer, U. (2001). Increased AT(1) receptor heterodimers in preeclampsia mediate enhanced angiotensin II responsiveness. Nat Med *7*, 1003-1009.
AbdAlla, S., Lother, H., Langer, A., el Faramawy, Y., and Quitterer, U. (2004). Factor XIIIA transglutaminase crosslinks AT(1) receptor dimers of monocytes at the onset of atherosclerosis. Cell *119*, 343-354.
Babcock, G.J., Farzan, M., and Sodroski, J. (2003). Ligand-independent dimerization of CXCR4, a principal HIV-1 coreceptor. J Biol Chem *278*, 3378-3385.
Baneres, J.L., and Parello, J. (2003). Structure-based analysis of GPCR function: Evidence for a novel pentameric assembly between the dimeric leukotriene B-4 receptor BLT1 and the G-protein. J Mol Biol *329*, 815-829.
Calebiro, D., Rieken, F., Wagner, J., Sungkaworn, T., Zabel, U., Borzi, A., Cocucci, E., Zuern, A., and Lohse, M.J. (2013). Single-molecule analysis of fluorescently labeled G-protein-coupled receptors reveals complexes with distinct dynamics and organization. Proc Natl Acad Sci U S A *110*, 743-748.
Carrillo, J.J., Pediani, J., and Milligan, G. (2003). Dimers of class A G protein-coupled receptors function via agonist-mediated trans-activation of associated G proteins. J Biol Chem *278*, 42578-42587.
Cheng, Z.J., Harikumar, K.G., Holicky, E.L., and Miller, L.J. (2003). Heterodimerization of type A and B cholecystokinin receptors enhance signaling and promote cell growth. J Biol Chem *278*, 52972-52979.
Damian, M., Mary, S., Martin, A., Pin, J.-P., and Baneres, J.-L. (2008). G protein activation by the leukotriene B(4) receptor dimer - Evidence for an absence of trans-activation. J Biol Chem *283*, 21084-21092.
Dinger, M.C., Bader, J.E., Kobor, A.D., Kretzschmar, A.K., and Beck-Sickinger, A.G. (2003). Homodimerization of neuropeptide y receptors investigated by fluorescence resonance energy transfer in living cells. J Biol Chem *278*, 10562-10571.
Dorsch, S., Klotz, K.N., Engelhardt, S., Lohse, M.J., and Bunemann, M. (2009). Analysis of receptor oligomerization by FRAP microscopy. Nat Methods *6*, 225-230.
Evans, N.J., and Walker, J.W. (2008). Endothelin receptor dimers evaluated by FRET, ligand binding, and calcium mobilization. Biophys J *95*, 483-492.
Floyd, D.H., Geva, A., Bruinsma, S.P., Overton, M.C., Blumer, K.J., and Baranski, T.J. (2003). C5a receptor oligomerization - II. Fluorescence resonance energy transfer studies of a human G protein-coupled receptor expressed in yeast. J Biol Chem *278*, 35354-35361.
Fukushima, Y., Asano, T., Saitoh, T., Anai, M., Funaki, M., Ogihara, T., Katagiri, H., Matsuhashi, N., Yazaki, Y., and Sugano, K. (1997). Oligomer formation of histamine H2 receptors expressed in Sf9 and COS7 cells. FEBS Lett *409*, 283-286.
Gandia, J., Galino, J., Amaral, O.B., Soriano, A., Lluis, C., Franco, R., and Ciruela, F. (2008). Detection of higher-order G protein-coupled receptor oligomers by a combined BRET-BiFC technique. FEBS Lett *582*, 2979-2984.
Grant, M., Collier, B., and Kumar, U. (2004). Agonist-dependent dissociation of human somatostatin receptor 2 dimers - A role in receptor trafficking. J Biol Chem *279*, 36179-36183.
Hammad, M.M., Kuang, Y.Q., Yan, R., Allen, H., and Dupre, D.J. (2010). Na+/H+ Exchanger Regulatory Factor-1 Is Involved in Chemokine Receptor Homodimer CCR5 Internalization and Signal Transduction but Does Not Affect CXCR4 Homodimer or CXCR4-CCR5 Heterodimer. J Biol Chem *285*, 34653-34664.
Hansen, J.L., Theilade, J., Haunso, S., and Sheikh, S.P. (2004). Oligomerization of wild type and nonfunctional mutant angiotensin II type I receptors inhibits G alpha(q) protein signaling but not ERK activation. J Biol Chem *279*, 24108-24115.
Herrick-Davis, K., Grinde, E., Harrigan, T.J., and Mazurkiewicz, J.E. (2005). Inhibition of serotonin 5-hydroxytryptamine2C receptor function through heterodimerization - Receptor dimers bind two molecules of ligand and one G-protein. J Biol Chem *280*, 40144-40151.
Herrick-Davis, K., Grinde, E., Lindsley, T., Cowan, A., and Mazurkiewicz, J.E. (2012). Oligomer Size of the Serotonin 5-Hydroxytryptamine 2C (5-HT2C) Receptor Revealed by Fluorescence Correlation Spectroscopy with Photon Counting Histogram Analysis. J Biol Chem *287*, 23604-23614.
Herrick-Davis, K., Weaver, B.A., Grinde, E., and Mazurkiewicz, J.E. (2006). Serotonin 5-HT2C receptor homodimer biogenesis in the endoplasmic reticulum - Real-time visualization with confocal fluorescence resonance energy transfer. J Biol Chem *281*, 27109-27116.
Huang, J., Chen, S., Zhang, J.J., and Huang, X.-Y. (2013). Crystal structure of oligomeric beta(1)-adrenergic G protein-coupled receptors in ligand-free basal state. Nature Structural & Molecular Biology *20*, 419-425.
James, J.R., Oliveira, M.I., Carmo, A.M., Iaboni, A., and Davis, S.J. (2006). A rigorous experimental framework for detecting protein oligomerization using bioluminescence resonance energy transfer. Nat Methods *3*, 1001-1006.

Kotevic, I., Kirschner, K.M., Porzig, H., and Baltensperger, K. (2005). Constitutive interaction of the P2Y(2) receptor with the hematopoietic cell-specific G protein G(alpha 16) and evidence for receptor oligomers. Cell Signal *17*, 869-880.
Laroche, G., Lepine, M.C., Theriault, C., Giguere, P., Giguere, V., Gallant, M.A., de Brum-Fernandes, A., and Parent, J.L. (2005). Oligomerization of the alpha and beta isoforms of the thromboxane A(2) receptor: Relevance to receptor signaling and endocytosis. Cell Signal *17*, 1373-1383.
Lee, K., Jung, Y., Lee, J.Y., Lee, W.K., Lim, D., and Yu, Y.G. (2012). Purification and characterization of recombinant human endothelin receptor type A. Protein Expr Purif *84*, 14-18.
Lee, S.P., O'Dowd, B.F., Rajaram, R.D., Nguyen, T., and George, S.R. (2003). D2 dopamine receptor homodimerization is mediated by multiple sites of interaction, including an intermolecular interaction involving transmembrane domain 4. Biochemistry *42*, 11023-11031.
Levoye, A., Dam, J., Ayoub, M.A., Guilllaume, J.L., Couturier, C., Delagrange, P., and Jockers, R. (2006). The orphan GPR50 receptor specifically inhibits MT1 melatonin receptor function through heterodimerization. Embo J *25*, 3012-3023.
Lukasiewicz, S., Blasiak, E., Faron-Gorecka, A., Polit, A., Tworzydlo, M., Gorecki, A., Wasylewski, J., and Dziedzicka-Wasylewska, M. (2007). Fluorescence studies of homooligomerization of adenosine A(2A) and serotonin 5-HT1A receptors reveal the specificity of receptor interactions in the plasma membrane. Pharmacol Rep *59*, 379-392.
Mancia, F., Assur, Z., Herman, A.G., Siegel, R., and Hendrickson, W.A. (2008). Ligand sensitivity in dimeric associations of the serotonin 5HT2c receptor. EMBO Rep *9*, 363-369.
Mercier, J.F., Salahpour, A., Angers, P., Breit, A., and Bouvier, M. (2003). Quantitative assessment of beta(1)- and beta(2)-adrenergic re-receptor homo- and heterodimerization by bioluminescence resonance energy transfer J Biol Chem *278*, 18704-18704.
Mesnier, D., and Baneres, J.L. (2004). Cooperative conformational changes in a G-protein-coupled receptor dimer, the leukotriene B-4 receptor BLT1. J Biol Chem *279*, 49664-49670.
Michineau, S., Alhenc-Gelas, F., and Rajerison, R.M. (2006). Human bradykinin B2 receptor sialylation and N-glycosylation participate with disulfide bonding in surface receptor dimerization. Biochemistry *45*, 2699-2707.
Nemoto, W., and Toh, H. (2005). Prediction of interfaces for oligomerizations of G-protein coupled receptors. Proteins *58*, 644-660.
Percherancier, Y., Berchiche, Y.A., Slight, I., Volkmer-Engert, R., Tamamura, H., Fujii, N., Bouvier, M., and Heveker, N. (2005). Bioluminescence resonance energy transfer reveals ligand-induced conformational changes in CXCR4 homo- and heterodimers. J Biol Chem *280*, 9895-9903.
Salim, K., Fenton, T., Bacha, J., Urien-Rodriguez, H., Bonnert, T., Skynner, H.A., and Watts, E. (2002). Oligomerization of G-protein-coupled receptors shown by selective co-immunoprecipitation. J Biol Chem *277*, 15482-15485.
Small, K.M., Schwarb, M.R., Glinka, C., Theiss, C.T., Brown, K.M., Seman, C.A., and Liggett, S.B. (2006). Alpha(2A)- and alpha(2C)-adrenergic receptors form homo- and heterodimers: The heterodimeric state impairs agonist-promoted GRK phosphorylation and beta-arrestin recruitment. Biochemistry *45*, 4760-4767.
Suzuki, T., Namba, K., Mizuno, N., and Nakata, H. (2013). Hetero-oligomerization and specificity changes of G protein-coupled purinergic receptors: novel insight into diversification of signal transduction. Methods in enzymology *521*, 239-257.
Tanaka, T., Nomura, W., Narumi, T., Masuda, A., and Tamamura, H. (2010). Bivalent Ligands of CXCR4 with Rigid Linkers for Elucidation of the Dimerization State in Cells. J Am Chem Soc *132*, 15899-15901.
Van Brocklyn, J.R., Behbahani, B., and Lee, N.H. (2002). Homodimerization and heterodimerization of SlP/EDG sphingosine-1-phosphate receptors. Biochim Biophys Acta Mol Cell Biol Lipids *1582*, 89-93.
Vidi, P.A., Chen, J.J., Irudayaraj, J.M.K., and Watts, V.J. (2008). Adenosine A(2A) receptors assemble into higher-order oligomers at the plasma membrane. FEBS Lett *582*, 3985-3990.
Wu, B.L., Chien, E.Y.T., Mol, C.D., Fenalti, G., Liu, W., Katritch, V., Abagyan, R., Brooun, A., Wells, P., Bi, F.C., *et al.* (2010). Structures of the CXCR4 Chemokine GPCR with Small-Molecule and Cyclic Peptide Antagonists. Science *330*, 1066-1071.
Zaslavsky, A., Singh, L.S., Tan, H.Y., Ding, H.W., Liang, Z.C., and Xu, Y. (2006). Homo- and hetero-dimerization of LPA/S1P receptors, OGR1 and GPR4. Biochim Biophys Acta Mol Cell Biol Lipids *1761*, 1200-1212.
