# Supplementary material for: Receptor Quaternary Organization Explains G Protein-Coupled Receptor Family Structure
Source: Cell Rep. 2017 Sep 12;20(11):2654–65. doi: 10.1016/j.celrep.2017.08.072 (PMC5608970; doi:10.1016/j.celrep.2017.08.072)
Supplement: Document S2. Article plus Supplemental Information [file mmc3.pdf]

Cell Reports

Article

# Receptor Quaternary Organization Explains G Protein-Coupled Receptor Family Structure

## Graphical Abstract

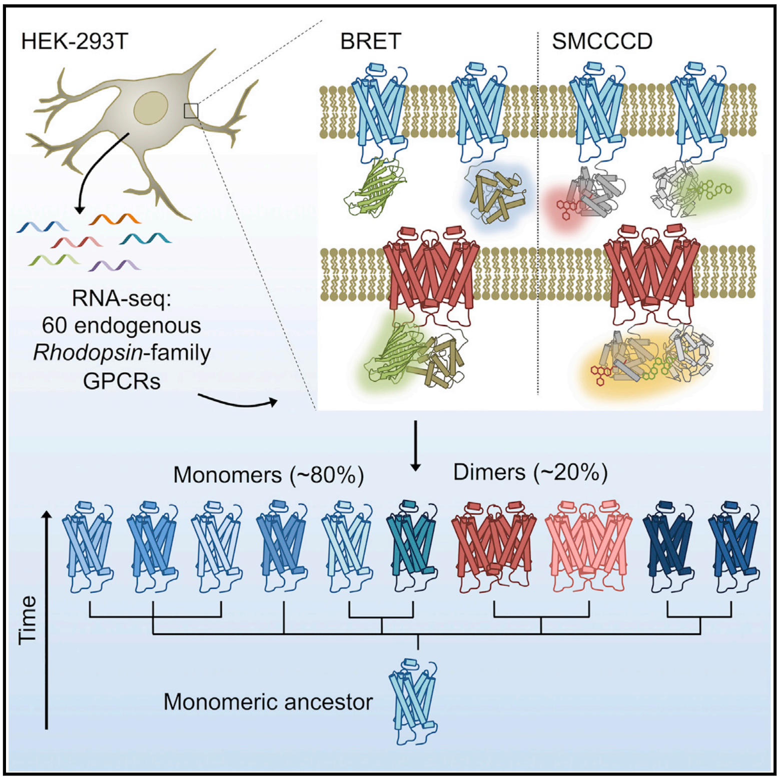


## Highlights

- Systematic analysis of 60 *Rhodopsin*-family GPCRs reveals that most are monomers
- *Rhodopsin*-family root ancestor GPCRs are also monomers
- Across families, rates of receptor diversification correlate with stoichiometry
- Skewed family structure suggests dimerization increases receptor "fitness density"

## Authors

James H. Felce, Sarah L. Latty, Rachel G. Knox, ..., Steven F. Lee, David Klenerman, Simon J. Davis

## Correspondence

dk10012@cam.ac.uk (D.K.),
simon.davis@imm.ox.ac.uk (S.J.D.)

## In Brief

The quaternary organization of *Rhodopsin*-family GPCRs is controversial. Felce et al. show that 60 receptors are mostly monomeric. They propose a simple explanation for the remarkable asymmetry in GPCR family structure, i.e., that it is underpinned by the lineage expansion of monomers rather than dimers.

## Data and Software Availability

GSE102461

Felce et al., 2017, Cell Reports *20*, 2654–2665
September 12, 2017 Crown Copyright © 2017
http://dx.doi.org/10.1016/j.celrep.2017.08.072

OPEN ACCESS
CellPress

Cell Reports
Article

# Receptor Quaternary Organization Explains G Protein-Coupled Receptor Family Structure

James H. Felce,[1] Sarah L. Latty,[2] Rachel G. Knox,[1] Susan R. Mattick,[1] Yuan Lui,[1] Steven F. Lee,[2] David Klenerman,[2,*] and Simon J. Davis[1,3,*]
[1]Radcliffe Department of Medicine and Medical Research Council Human Immunology Unit, Weatherall Institute of Molecular Medicine, University of Oxford, Oxford OX3 9DS, UK
[2]Department of Chemistry, University of Cambridge, Cambridge CB2 1EW, UK
[3]Lead Contact
*Correspondence: dk10012@cam.ac.uk (D.K.), simon.davis@imm.ox.ac.uk (S.J.D.)
http://dx.doi.org/10.1016/j.celrep.2017.08.072

## SUMMARY

**The organization of *Rhodopsin*-family G protein-coupled receptors (GPCRs) at the cell surface is controversial. Support both for and against the existence of dimers has been obtained in studies of mostly individual receptors. Here, we use a large-scale comparative study to examine the stoichiometric signatures of 60 receptors expressed by a single human cell line. Using bioluminescence resonance energy transfer- and single-molecule microscopy-based assays, we found that a relatively small fraction of *Rhodopsin*-family GPCRs behaved as dimers and that these receptors otherwise appear to be monomeric. Overall, the analysis predicted that fewer than 20% of ∼700 *Rhodopsin*-family receptors form dimers. The clustered distribution of the dimers in our sample and a striking correlation between receptor organization and GPCR family size that we also uncover each suggest that receptor stoichiometry might have profoundly influenced GPCR expansion and diversification.**

## INTRODUCTION

G protein-coupled receptors (GPCRs) are organized into six main families: the *Glutamate*, *Rhodopsin*, *Adhesion*, *Frizzled*, *Secretin*, and *Taste2* families (Fredriksson et al., 2003). A striking feature of GPCR family structure is the overwhelming dominance of the *Rhodopsin* (class A) family, which comprises >80% of all human GPCRs and a similar fraction of the GPCRs expressed by other vertebrates (Fredriksson and Schioth, 2005). GPCRs all consist of a core of seven transmembrane (TM) α helices joined by six interhelical loops of variable length. The loops combine with the N and C termini forming, respectively, an extracellular region that, together with the TM region, creates the ligand-binding site and a cytoplasmic region that interacts with secondary signaling components, e.g., G proteins. The organization of the TM region is strikingly similar across all GPCRs for which structures have been obtained and is stabilized by a conserved network of interactions between topologically equivalent residues (Venkatakrishnan et al., 2013). The most significant structural variation between GPCRs is restricted to the ligand-binding regions, and the parts of the receptors involved in signal transduction are typically much more highly conserved (Katritch et al., 2012), allowing similar conformational changes to accompany receptor activation (Deupi and Standfuss, 2011). Several studies of isolated GPCRs (Bayburt et al., 2007; Ernst et al., 2007; Kuszak et al., 2009; Leitz et al., 2006; Whorton et al., 2007) convincingly show that signal transduction can occur on the scale of single, autonomous receptors, consistent with GPCRs forming 1:1 complexes with G proteins (Rasmussen et al., 2011).

Without question, the most contentious aspect of GPCR biology concerns their quaternary structures. This is not an insignificant issue, as homo- or hetero-oligomer formation offers, e.g., a simple explanation for a wealth of pharmacological data implying that receptors engage in "cross-talk" (although other explanations are possible; Chabre et al., 2009; Tubio et al., 2010) and new opportunities for pharmacological intervention. Whereas several small families of GPCRs comprise receptors whose large N- and C-terminal domains are known to effect dimerization, e.g., the *Glutamate* (class C) receptors (Gurevich and Gurevich, 2008b), there is no consensus regarding the "typical" quaternary structure of the largest group of GPCRs, i.e., the *Rhodopsin* family. It was initially thought that *Rhodopsin*-family GPCRs are generally monomeric, but the more prevalent view now (Pétrin and Hebert, 2012; Pfleger and Eidne, 2005) is that these receptors form transiently associating or stable dimeric and oligomeric complexes, with implications for their signaling behavior (the cases for and against oligomerization have been summarized by Bouvier and Hebert [2014] and by Lambert and Javitch [2014]). The first applications of resonance energy transfer (RET)-based assays seemed to precipitate this shift in thinking, but these assays are prone to difficulties in distinguishing genuine interactions from chance co-localizations, and the interpretation of some early studies is disputed (Chabre et al., 2009; Chabre and le Maire, 2005; Felce and Davis, 2012; James et al., 2006). More recently, single-molecule measurements have failed to demonstrate constitutive oligomerization in transfected and native cells (Cai et al., 2017; Hern et al., 2010; Jonas et al., 2015; Kasai et al., 2011; Latty et al., 2015; Nenasheva et al., 2013), with one exception (Calebiro et al., 2013). Equally, lattice contacts in GPCR crystals tend to argue against dimeric interactions, and, where putative dimers have been observed, the proposed interfaces were not conserved

 Crown Copyright © 2017
This is an open access article under the CC BY license (http://creativecommons.org/licenses/by/4.0/).

(Huang et al., 2013; Manglik et al., 2012; Salom et al., 2006; Wu et al., 2010). It remains a possibility, however, that dimeric *Rhodopsin*-family GPCRs exist, but only rarely (e.g., Gurevich and Gurevich, 2008a). With few exceptions (e.g., Calebiro et al., 2013), differences in receptor behavior have not been reported in individual studies, although systematic, comparative analyses of GPCR organization have not been undertaken.

Of the RET-based approaches for studying GPCR stoichiometry, which still represent the highest resolution (<10 nm) in situ assays, bioluminescence RET (BRET) is the most widely used, because it is relatively straightforward and uncomplicated by photobleaching and photoconversion effects confounding Förster RET-based measurements. We have established three BRET-based assays (types-1 to -3; Felce et al., 2014; James et al., 2006), each indicating that human $\beta_2$-adrenergic receptor ($\beta_2$AR) and mouse cannabinoid receptor 2 (mCannR2) are monomers. Here, we report a systematic analysis of the stoichiometry of 60 *Rhodopsin*-family GPCRs using two of these assays and a single-molecule fluorescence-based assay (Latty et al., 2015). We found (1) that a small fraction of *Rhodopsin*-family GPCRs formed authentic dimers and that these receptors were otherwise monomeric, (2) that dimers comprised closely related phylogenetic clusters, (3) that these receptor clusters did not share ligand or G protein selectivity outside the clusters, and (4) that even closely related receptors could have different stoichiometries. These findings suggest a simple explanation for the remarkable asymmetry in GPCR family structure, i.e., that it is underpinned by the lineage expansion of monomers rather than dimers.

## RESULTS

### BRET Assay Sensitivity

The BRET assays used in this study are described in detail elsewhere (Felce et al., 2014; James et al., 2006). Briefly, in type-1 BRET experiments (James et al., 2006), the ratio of acceptor- to donor-tagged proteins is varied at constant expression, resulting in a hyperbolic relationship between energy transfer efficiency ($BRET_{eff}$) and acceptor:donor ratio for dimers (the principles of the assays are illustrated in Figure S1A). For monomers, as confirmed for type-1 receptors of known stoichiometry (James et al., 2006), $BRET_{eff}$ is effectively independent of this ratio above a certain threshold. Stoichiometry is indicated by $R^2$ values for the data fitted to monomer versus dimer models (Figures S1B and S1C). In type-3 BRET assays, untagged "competitor" receptors reduce $BRET_{eff}$ for dimers, but not monomers, for a range of expression levels (Figure S1A), with stoichiometry confirmed by the likelihood ($p^{diff}$) that $BRET_{eff}$ is affected by the competitor (Figure S1D; Felce et al., 2014). These assays are complementary insofar as type-1 assays are not prone to false-dimer artifacts but could, in principle, give false-monomer results in cases of higher order oligomerization or very weak dimerization, whereas type-3 assays avoid false-monomer results but could produce false-dimer signals, e.g., when the addition of competitor proteins causes the clustering of tagged receptors to be relaxed, reducing effective density (Figure S1A). Concordant data obtained with these assays, therefore, afford confident assignment of receptor stoichiometry.

We undertook a systematic exploration of *Rhodopsin*-family GPCR stoichiometry using both type-1 and -3 BRET assays. By focusing on the set of GPCRs expressed by HEK293T cells, the host cell typically used for BRET assays (Pfleger and Eidne, 2005), we could characterize receptor behavior in its native cellular milieu. The choice of cell line precluded the use of type-2 assays reliant on observations made at very low expression levels (James et al., 2006), because this type of analysis would have been complicated by the presence of untagged, native receptors. As reported previously (e.g., Barak et al., 1997), C-terminal tagging of two example GPCRs (i.e., human $\beta_1$-adrenergic receptor [$\beta_1$AR] and $\beta_2$AR) with GFP and Rluc did not alter their responses to agonists (Figure S2A). The sensitivity of the type-1 and -3 BRET assays was first tested using an inducible system for generating dimers. The monomeric receptor CD86 was fused to the FK506-binding protein (FKBP), allowing the bivalent FKBP ligand AP20187 to induce various levels of receptor dimerization (Figures S2B and S2C). Type-1 and -3 BRET assays detected dimers comprising as few as 20% of the total receptor population (Figures S2D–S2F and S2I–S2Q); similar data were obtained for induced $\beta_2$AR dimers (Figures S2D, S2G, S2H, and S2R–S2Z). For simplicity, we hereinafter refer to two classes of receptors: "dimers" ($\geq$20% dimerization) and "monomers" (<20% dimerization).

### Two Types of *Rhodopsin*-Family GPCR Behavior

HEK293T-cell-expressed GPCRs were identified by mining the Universal Protein Resource (UniProt) database (www.uniprot.org) and comparing the results to gene expression data generated by deep sequencing (RNA sequencing; RNA-seq) of the HEK293T cell transcriptome. mRNA encoding 65 *Rhodopsin*-family GPCRs was detected in HEK293T cells (Data S1, "Receptors"), with assignment of receptors to the *Rhodopsin* family based mostly on published phylogenetic analyses (Fredriksson et al., 2003). These receptors comprised a cross-section of *Rhodopsin*-family GPCRs, with a diverse range of physiological functions and ligands, although some important receptor families were not represented, e.g., the dopamine receptors, and some receptors may only exist at the RNA level in HEK293T cells. Transient transfection of GPCRs in the form of GFP fusion proteins in HEK293T cells gave expression levels of ~100,000 per cell (Data S1, "BRET Experiments"), as determined by flow-cytometric analysis. This included expression on intracellular membranes, consistent with many GPCRs residing mostly in internal membranes until stimulated to traffic to the cell surface (e.g., Brismar et al., 1998; Hein et al., 1994). These expression levels were 1–2 orders of magnitude higher than that of native receptors (e.g., Hegener et al., 2004; Nenasheva et al., 2013), avoiding interference of the assays by homo- and heteromeric interactions with native receptors.

Of the 65 receptors, 60 had sufficiently good trafficking and expression characteristics to allow BRET analysis (Figure S3; Table S1). Type-1 and type-3 BRET analysis of 57 of the 60 GPCRs yielded concordant data suggesting that *Rhodopsin*-family GPCRs exist in two stoichiometric states. Representative datasets for the monomeric lysophosphatidic acid (LPA) and dimeric sphingosine-1-phosphate (S1P) receptors are shown in Figures 1A–1C. The $R^2$ and $p^{diff}$ values are plotted in Figure 1D,

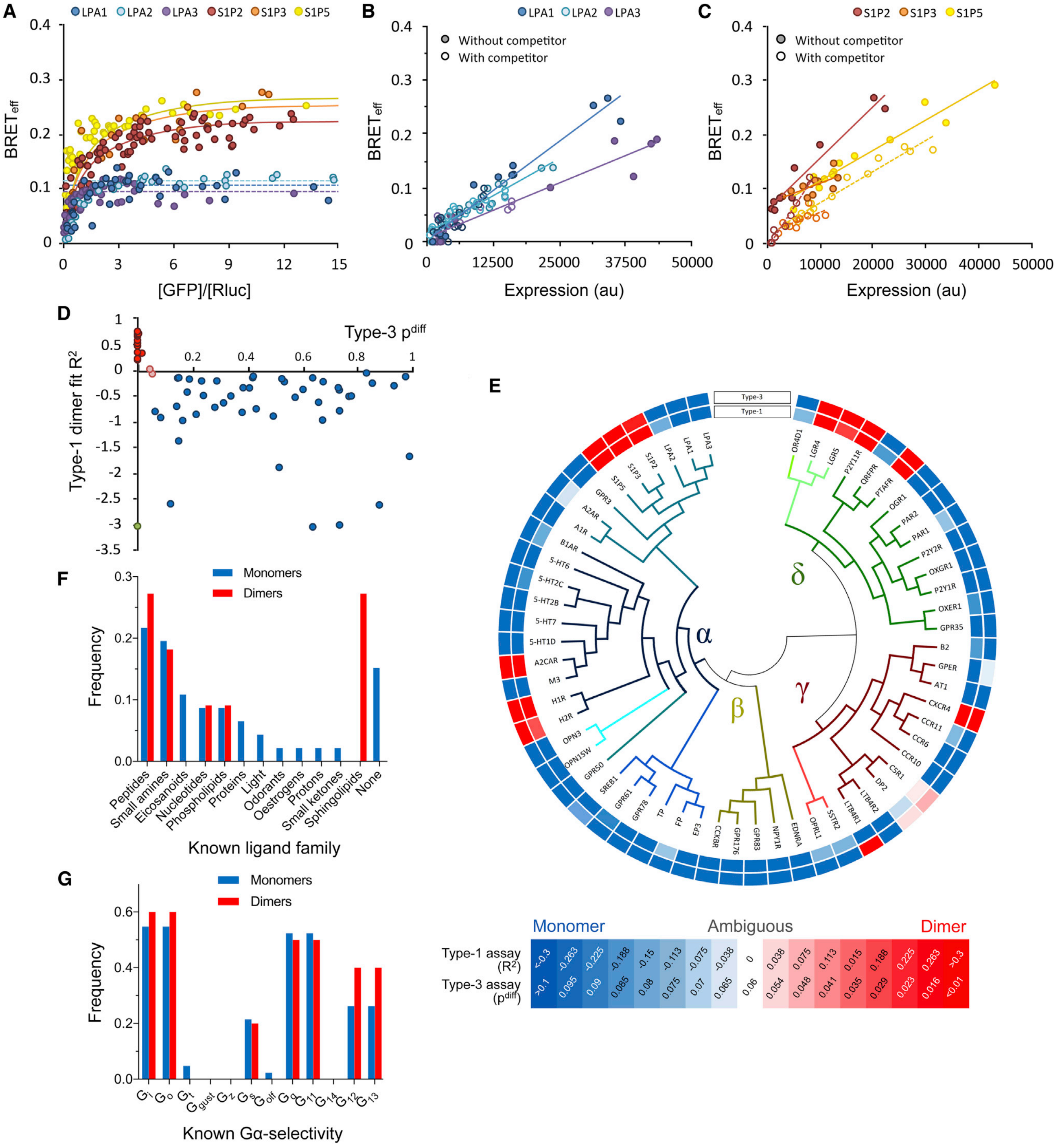


**Figure 1. Two Stoichiometric Classes of *Rhodopsin*-Family GPCRs Revealed by Type-1 and -3 BRET Assays**

(A) Type-1 BRET data for HEK293T-cell-expressed S1P and LPA receptors as representatives of two stoichiometric classes of *Rhodopsin*-family GPCRs. Optimal fits of the data are shown as a solid line for a dimer model, and as a broken line for a monomer model.

(B) Type-3 BRET data for the LPA receptors, revealing monomeric behavior consistent with the type-1 analysis. Data collected in both the absence and presence of competitor proteins are shown as filled and empty circles, respectively. A single fit of all data is shown as a solid line.

(C) Type-3 BRET data for the S1P receptors, revealing dimeric behavior consistent with the type-1 analysis. Data fits are shown as solid lines (no competitor) and broken lines (with competitor).

*(legend continued on next page)*

and absolute values are given in Data S1 ("BRET Experiments"). The results of the analysis, arranged according to *Rhodopsin*-family substructure, are shown schematically in Figure 1E. For each type-1 assay, total receptor expression was independent of [GFP]/[Rluc] (Data S1, "BRET Experiments") as required by the method (Figure S1A). In this assay, the maximum energy transfer efficiency ($BRET_{max}$) correlated with stoichiometry (i.e., typically higher for dimers than for monomers) and, to a lesser extent, with total expression level (Figure S4A; Data S1, "BRET Experiments"). In the type-3 assay, all dimers gave significantly non-zero projected y-intercepts, whereas, for the monomers, the y-intercepts were not significantly non-zero (Figure S4B; Data S1, "BRET Experiments"). It needs to be noted, however, that the ab initio interpretation of $BRET_{max}$ and the y-intercepts is not straightforward. $BRET_{max}$ does not directly report the extent of dimerization and is, instead, determined by many factors, including receptor density, clustering, and subunit geometry. The type-3 assay y-intercept is also subject to confounding effects arising from high expression. Therefore, while consistent with our assignments, these metrics were not used to assign stoichiometry to individual receptors.

Of the 60 receptors investigated, 46 exhibited monomeric behavior in both assays, indicating that they were either wholly monomeric or formed dimers at levels below the sensitivity of the assays. Eleven receptors behaved as dimers in both assays. Two receptors, C5R1 and DP2, yielded data that could not be easily assigned to either form of behavior, possibly due to dimerization at levels near the sensitivity limits of the assays. Only one receptor, LTB4R1, yielded conflicting data in the two assays, perhaps due to high-order oligomerization, which cannot be unambiguously excluded by the type-1 assay but is readily detected in type-3 assays. LTB4R1 was the only receptor for which data suggestive of high-order oligomerization was obtained, and it is unclear whether it is a bona fide oligomer and/or whether other *Rhodopsin*-family GPCRs can form high-order oligomers. The monomeric and dimeric populations exhibit no obvious differences in either ligand- or G protein specificity (Figures 1F and 1G). Moreover, the stoichiometric assignments do not correlate with C-terminal domain length (Figure S4C), implying that the cytoplasmic domains did not impose donor-acceptor separation distances greater than the RET-permissive radius (10 nm), leading to the false identification of monomers. Conversely, dimerization is not explained as an artifact of poor trafficking or expression, since the fraction of dimers did not correlate with apparent trafficking behavior (Figure S4D). Similarly, although heterodimerization with native GPCRs and/or TM proteins could conceivably restrict homodimerization, this seems very unlikely at the high levels of receptor expression used for BRET assays. For the dimers, we are unable to speculate about the strength of dimerization, as our analysis produces binary outcomes (i.e., fits that are closer to either a monomer or dimer model); however, a range of dimer stabilities could reasonably be expected. Models of partial dimerization could, in principle, be fitted to our data but would not be informative within the confidence limits of the assay.

## Single-Molecule Analysis

To further confirm that *Rhodopsin*-family GPCRs exist in more than one stoichiometric state, we used single-molecule cross-color coincidence detection (SMCCCD; Latty et al., 2015). Briefly, candidate GPCRs were transiently expressed in Chinese hamster ovary (CHO) K1 cells under the control of a weak promoter to ensure approximately physiological, i.e., low levels of expression. Unlike HEK293T cells, CHO K1 cells do not express homologs of any of the receptors studied using SMCCCD (Baycin-Hizal et al., 2012), thereby avoiding interference with the single-molecule analysis. Constructs consisting of the receptor fused with a C-terminal HaloTag or a SNAP-tag were co-expressed and then labeled with HaloTag-TMR Ligand and SNAP-Cell 505 Star. This allowed individual receptors to be localized each in one of two colors, and the degree of co-localization to be compared to the known monomeric and dimeric controls, CD86 and CD28, respectively. Coincidence values represent the fraction of HaloTag-labeled receptors that localize to within 300 nm of a SNAP-tag labeled receptor, presented as the mean for all cells analyzed. The principle of the method is summarized in Figure S1A.

Receptors exhibiting contrasting behavior in the BRET assays were selected for the SMCCCD analysis: both the S1P receptor 3 (S1P3) and $\alpha_{2C}$-adrenergic receptor ($\alpha_{2C}$AR) behaved as dimers, whereas LPA receptor 1 (LPA1) and $\beta_1$-adrenergic receptor ($\beta_1$AR) exhibited monomeric behavior. Consistent with the BRET analysis, in the single-molecule assay, S1P3 and $\alpha_{2C}$AR exhibited above-background levels of cross-color coincidence characteristic of dimers, whereas the LPA1 and $\beta_1$AR receptors exhibited monomer control levels of cross-color coincidence (Figure 2; Table S2). The measured coincidence level was considerably higher for S1P3 than for $\alpha_{2C}$AR, however, which was only slightly higher than that for the monomer control, CD86. It is, therefore, possible that, at physiological expression levels, $\alpha_{2C}$AR is only a weak dimer. We cannot formally exclude the possibility that the coincidence observed in this assay is the product of indirect receptor co-localization rather than direct physical association, given that our observations are diffraction limited to a resolution of $\sim$300 nm, $\sim$60-fold larger than the hydrodynamic diameter of most GPCRs. However, this would not be consistent with the results of the BRET assays.

(D) $R^2$ (type-1 assay) and $p^{diff}$ (type-3 assay) values for *Rhodopsin*-family GPCRs. Monomers are indicated in blue; dimers are indicated in red. Dimers cluster in the region of high $R^2$ and low $p^{diff}$. C5R1 and DP2 (pink) are ambiguous, because they lie near the boundary for monomer versus dimer identification in both assays. LTB4R1 (green) is the only receptor to yield conflicting results in the two assays, possibly due to high-order oligomerization.

(E) Outcomes for all *Rhodopsin*-family receptors investigated. A gradient (from monomer, in blue, to dimer, in red) is colored according to the $R^2$ and $p^{diff}$ values shown in the key. The inner circle gives the type-1 assay result; the outer circle gives the type-3 assay result. Receptor relationships are shown as a divergence tree (Fredriksson et al., 2003).

(F) Known ligand preferences of the monomeric and dimeric receptors.

(G) G protein selectivity of the monomers and dimers.

See also Figures S1, S2, S3, and S4, and Data S1 ("BRET Experiments").

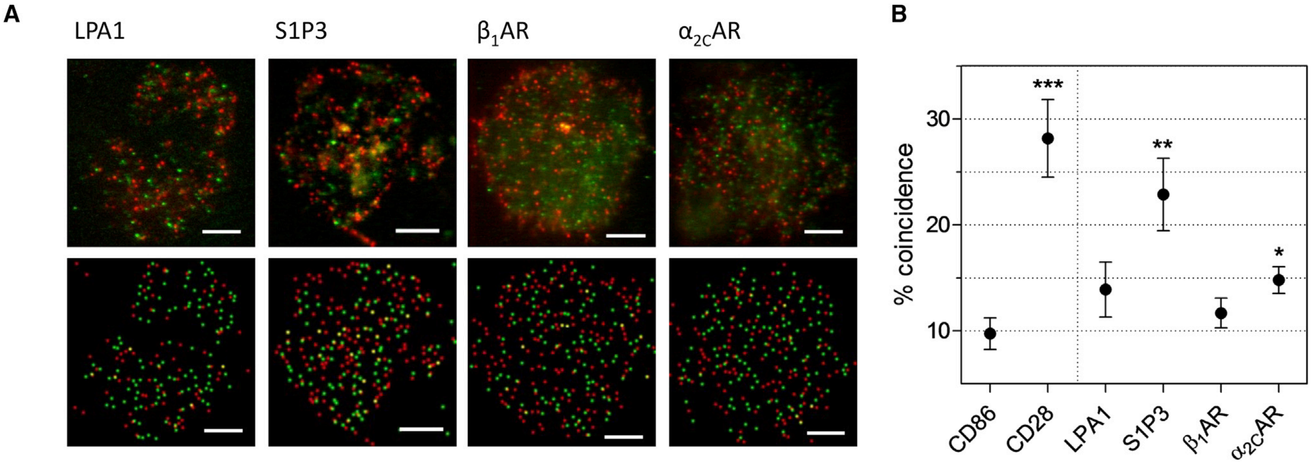


**Figure 2. Single-Molecule Microscopy Confirms the Existence of Two Stoichiometric Classes of *Rhodopsin*-Family GPCRs**

(A) Representative actual data (top) and reconstructed spot detection (bottom) for transfected HaloTag-labeled (red) and SNAP-tag-labeled (green) proteins expressed in CHO K1 cells. Scale bars, 5 μm. LPA1, S1P3, $\beta_1$AR, and $\alpha_{2C}$AR were expressed as C-terminal SNAP-tag- and HaloTag-fusion proteins for two-color labeling.

(B) Cross-color coincidence values for GPCRs investigated using SMCCCD. S1P3 and $\alpha_{2C}$AR exhibit levels of coincidence significantly higher than that for the strict monomer, CD86, but lower than that for the covalent dimer, CD28. Coincidence values for both LPA1 and $\beta_1$AR were not significantly higher than that for CD86. The data confirmed the observations made using BRET. Error bars indicate mean ± SE. *$p < 0.05$; **$p < 0.01$; ***$p < 0.005$ (two-tailed t test of difference to CD86). See also Table S2.

## Dimerization of S1P3 via TM Helix 4

The contrasting behavior of the otherwise closely related LPA and S1P subgroups allowed coarse mapping of the dimerization interface. In preliminary experiments, three LPA1/S1P3 chimeras were generated with various contributions from each receptor (Figure S5A), and these expressed well enough for BRET analysis (Figure S5B). Type-1 BRET analysis of the chimeras indicated that the presence of the N-terminal domain, TM helix 1, intracellular loop (IL) 1, and TM2 of S1P3 ("chimera 1") were not sufficient to induce LPA1 to form dimers, whereas the inclusion also of extracellular loop (EL) 1, TM3, IL2, and TM4 ("chimera 2") resulted in chimera dimerization (Figure S5C; Data S1, "BRET Experiments"). Including additional S1P3 domains had no further effect on receptor stoichiometry ("chimera 3"; Figure S5C). This suggested that the sequence motifs mediating interaction in S1P3 are likely predominantly located within the EL1, TM3, IL2, and TM4 regions. The contributions of these regions to S1P3 dimerization were further dissected using reciprocal swaps of the individual domains (Figure 3A). Type-1 and type-3 BRET assays identified TM4 as the principal site of dimerization, since the transfer of LPA1 TM4 to S1P3 partially abrogated S1P3 dimerization (Figures 3B and 3C), and transfer of S1P3 TM4 induced LPA1 dimerization (Figures 3D and 3E; Data S1, "BRET Experiments").

## Correlation of GPCR Family Size and Stoichiometry

Our analysis reveals that most *Rhodopsin*-family GPCRs expressed by HEK293T cells are monomers. The phylogenetic distribution of the dimers that we have identified appears to be non-random, however. Of the eleven dimers, seven are closely related to other dimers (Figure 1E), forming clusters: the S1P receptors S1P2, S1P3, and S1P5; the histamine receptors H1R and H2R; and the leucine-rich repeat-containing receptors LGR4 and LGR5. In addition to reinforcing the BRET-based assignments, these data suggest that the evolutionary appearance of dimers might be rare and episodic. Moreover, the contrasting stoichiometries of the large *Rhodopsin* family and the small *Glutamate* family (Gurevich and Gurevich, 2008b) are suggestive of there being a correlation between predominantly dimeric behavior and restricted family size. To test whether stoichiometry correlates with GPCR family size, we examined the *Frizzled* GPCRs, which appeared contemporaneously with *Rhodopsin*-family GPCRs but comprise only 11 receptors, and *Taste2* GPCRs, which emerged and separated from the *Rhodopsin* family just ~300 million years ago but already comprise more than 28 members (Nordstrom et al., 2011) exhibiting significant diversification (Kim et al., 2005). Seven *Frizzled* and four *Taste2* HEK293T-derived GPCRs expressed well enough for BRET analysis (Figure S3). In type-1 and -3 assays, the *Frizzled* receptors all behaved as dimers, whereas the *Taste2* receptors exhibited only monomeric behavior (Figure 4; Data S1, "BRET Experiments"), suggesting that fast-diverging receptors might generally be monomeric and that receptors exhibiting less diversification are more often dimers. Chimeric receptors in which the N-terminal, C-terminal, and TM domains of a *Frizzled* (FZD10) receptor and a *Taste2* (TAS2R19) receptor were recombined implicated the N- and C-terminal regions of FZD10 in its dimerization, rather than the TM region (Figures S5D–S5F; Data S1, "BRET Experiments").

## Stoichiometry of *Rhodopsin*-Family Root-Ancestor GPCRs

The *Rhodopsin* family is thought to have emerged ~1.3 billion years ago from the *cAMP* GPCR family (Nordstrom et al., 2011), which was subsequently lost from vertebrates. We

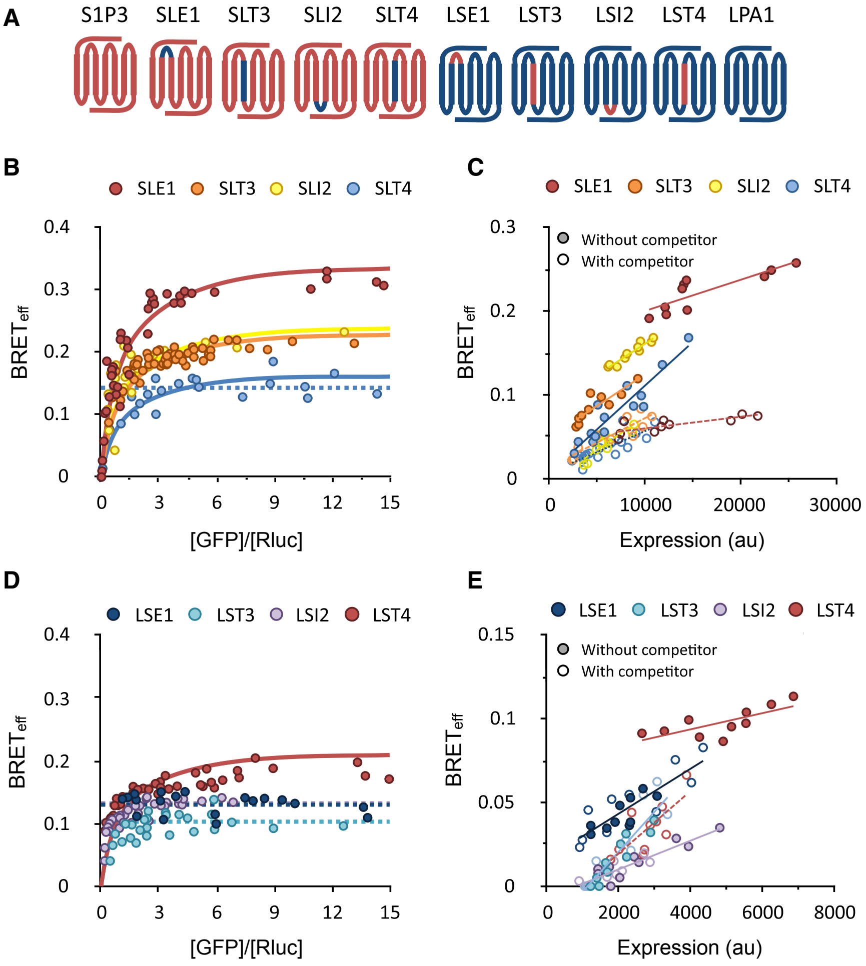


**Figure 3. S1P3 Dimerization Is Mediated Primarily by Interactions Involving TM4**

(A) Schematic representation of the S1P3/LPA1 chimeras studied, indicating the contributions of the S1P3 (red) and LPA1 (blue) sequences. TM helices are arranged from 1 to 7, left to right.

(B) Type-1 BRET analysis of S1P3 chimeras containing LPA1 domains. The data indicate that all of the constructs formed dimers, with SLT4 exhibiting the worst fit to a dimer model, suggesting weaker dimerization. For SLT4, the fit to a monomer model is also shown (broken line).

(C) Type-3 BRET analysis of S1P3 chimeras containing LPA1 domains, confirming the findings of the type-1 analysis.

(D) Type-1 BRET analysis of LPA1 chimeras containing S1P3 domains. Only LST4, with the TM4 domain of S1P3, formed detectable dimers.

(E) Type-3 BRET analysis of LPA1 chimeras containing S1P3 domains, confirming the findings of the type-1 analysis.

See also Figure S5 and Data S1 ("BRET Experiments").

examined the stoichiometry of three root-ancestor, non-vertebrate *cAMP* family GPCRs in type-1 and type-3 BRET assays. CrlC and CarB are from *Dictyostelium discoideum*, and a receptor we call CLP (*cAMP*-like receptor in Paramecium) is the sole *cAMP*-like GPCR expressed by *Paramecium tetraurelia*. We expect that, because the two species are evolutionarily distant (*D. discoideum* belongs to the amoebozoans, and *P. tetraurelia* belongs in the chromalveolata kingdom), similarities in their behavior will likely reflect the properties of *cAMP* GPCRs generally. All three *cAMP*-family receptors exhibited monomeric behavior in the two BRET assays (Figures 5A–5D; Data S1, "BRET Experiments").

## DISCUSSION

The stoichiometry of *Rhodopsin*-family GPCRs has been very contentious. Part of the controversy has centered on how best to implement RET measurements in studies of these receptors (Bouvier et al., 2007; James and Davis, 2007; James et al., 2006; Salahpour and Masri, 2007). Acquired in differently formatted assays, BRET data were used, for example, to support claims that $\beta_2$AR is an obligate dimer (Angers et al., 2000; Mercier et al., 2002; Ramsay et al., 2002) or that it is constitutively monomeric (Felce et al., 2014; James et al., 2006). *Rhodopsin*-family monomers and dimers were never identified in any single study, however, leaving open the formal possibility that these assays are incapable of detecting both types of behavior. Here, using two complementary BRET-based assays, we identified multiple examples of *Rhodopsin*-family GPCRs, each exhibiting one of two distinct types of behavior, one characteristic of substantive dimers and the other characteristic of monomers. For pairs of monomers and dimers identified in the BRET assays, we observed the same type of behavior in a third, orthogonal single-molecule fluorescence-based assay. For one of the dimers, we tentatively identified the core of the dimerizing interface as TM helix 4 of the receptor. These data, therefore, strongly suggest that *Rhodopsin*-family GPCRs comprise both monomers and dimers, with the more "typical" behavior being that of a monomer. Although we cannot draw direct conclusions regarding ligand-induced dimerization from these data, we anticipate that the observed resting-state behavior of the monomers likely reflects a general tendency toward constitutive monomeric behavior. Similarly, whether the dimers we detect can also heterodimerize with closely related monomeric or dimeric receptors, as has been claimed for CXCR4 (e.g., Contento et al., 2008), remains to be determined.

Our analysis indicates that as many as 20% of the ~700 *Rhodopsin*-family receptors may form dimers, distributed in discrete clusters across the family (Figure 5E). We did not attempt to measure the strength of dimerization, and it is unclear what fraction of these receptors, if any, dimerize constitutively rather than transiently. S1P3, which, at very low expression levels in the SMCCCD assay, produced a

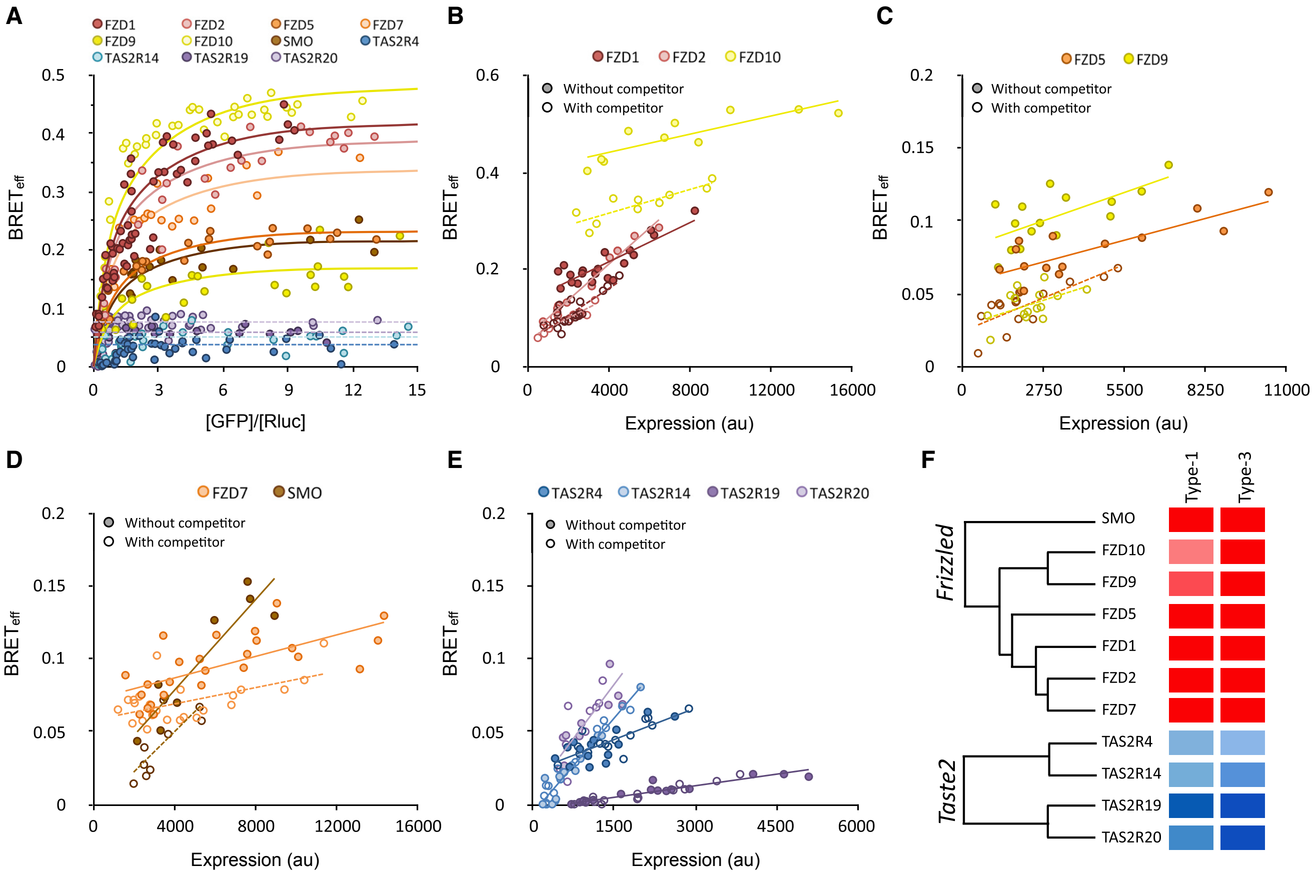


**Figure 4. *Fizzled*- and *Taste2*-Family GPCRs Comprise Monomers and Dimers, Respectively**

(A) Type-1 BRET analysis of seven *Frizzled* and four *Taste2* receptors. The *Frizzled* receptors all formed dimers, whereas the *Taste2* receptors behaved as monomers.

(B) Type-3 BRET analysis of the FZD1, FZD2, and FZD10 receptors, confirming the findings of the type-1 analysis.

(C) Type-3 BRET analysis of FZD5 and FZD9.

(D) Type-3 BRET analysis of FZD7 and SMO.

(E) Type-3 BRET analysis of the *Taste2* receptors.

(F) Results of the *Frizzled* and *Taste2* receptor assays summarized as in Figure 1E.

See also Figure S5 and Data S1 ("BRET Experiments").

coincidence signal indistinguishable from that of our covalent dimer control, CD28, seems to be a good candidate for constitutive receptor homodimerization, however, as does CXCR4, given the very high $BRET_{max}$ measured in our type-1 assay. On the other hand, the BRET measurements were performed under conditions of very high expression, and it is possible that, due to the effects of mass action, a smaller fraction of *Rhodopsin*-family GPCRs dimerize at lower levels of physiological expression. This behavior seems to be exemplified by $\alpha_{2C}$AR. In the BRET experiments, $\alpha_{2C}$AR exhibited clear-cut dimeric behavior, but only marginally higher levels of co-association than our control monomer, CD86, at the more physiological expression levels of the SMCCCD experiments. For the large set of receptors we sampled using BRET, we are, therefore, likely to have identified the upper limit of the fraction that form dimers. That only one receptor, LTB4R1, gave data suggestive of higher order oligomerization implies that such structures could be very rare.

Our observations are, nevertheless, strongly at odds with the notion that *Rhodopsin*-family GPCRs form constitutive dimers. It is noteworthy that, of 26 receptors reported previously to be dimers, often in multiple publications (Table S3), 21 behaved as monomers in our assays. Our data are in closer agreement with the increasing number of single-molecule microscopy experiments that have failed to identify constitutive dimers in most cases (AbdAlla et al., 2001; Hern et al., 2010; Jonas et al., 2015; Kasai et al., 2011; Kuszak et al., 2009; Latty et al., 2015; Nenasheva et al., 2013). The present data also argue against a general model of allosteric regulation of GPCR homodimers founded, principally, upon pharmacological analysis. Instead, cooperative effects observed between receptors could arise from indirect cross-talk between monomers. Such effects could be caused in vitro by limiting GTP levels and in vivo by competition between receptors for shared G proteins, each of which could affect ligand binding (both mechanisms are discussed in more detail by Chabre et al., 2009).

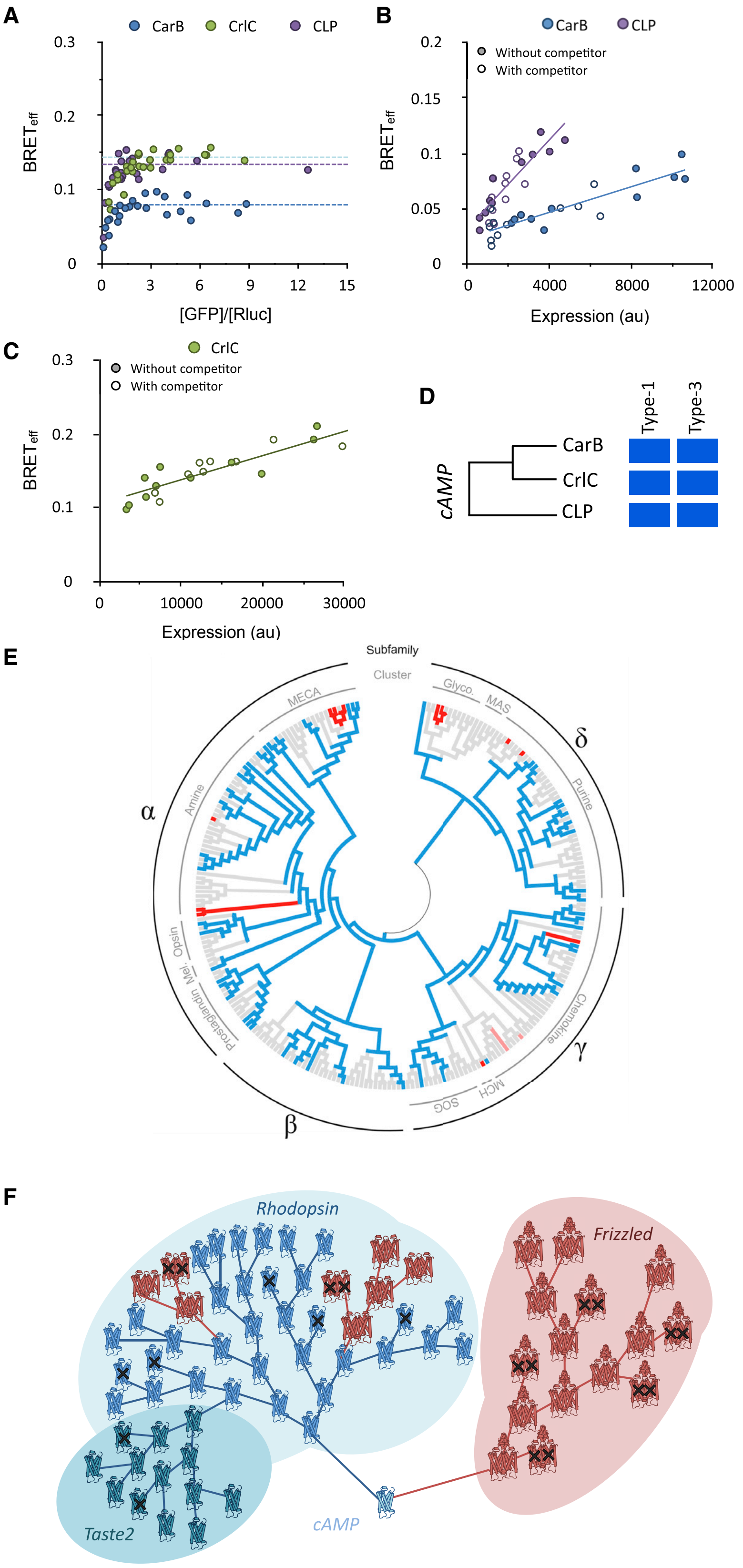


**Figure 5. Root Ancestor *cAMP*-Family Receptors Behave as Monomers in Type-1 and -3 BRET Assays**

(A) Type-1 BRET analysis of three *cAMP*-family receptors.

(B) Type-3 BRET analysis of CarB and CLP.

(C) Type-3 BRET analysis of CrlC.

(D) Results of the assays summarized as in Figure 1E.

(E) Modeled lineage tree for the *Rhodopsin* family, highlighting the episodic nature of gain-of-dimerization events. The tree shows all non-olfactory *Rhodopsin*-family GPCRs along with their subfamilies and clusters (Fredriksson et al., 2003). Colored branch endpoints indicate receptors investigated in this study: red for dimers, blue for monomers, pink for C5R1 and DP2. Receptors on gray branches were not investigated. Nodes are colored according to their predicted stoichiometry assuming the spontaneous emergence of dimers. The distribution of *Rhodopsin*-family dimers suggests that ≥ 7 independent gain-of-dimerization or stabilization-of-dimerization events have occurred during the evolution of this family of receptors.

(F) Evolutionary model of GPCR family expansion in which dimers (red) are constrained in their diversification compared to monomers (blue). Receptors carrying deleterious mutations are marked with a cross. These receptors either are lost during natural selection or degrade into pseudogenes over time. Relationships between receptors are represented as a simple lineage tree.

See also Data S1 (“BRET Experiments”).

Similarly, our observation that the dimerization of *Frizzled* receptors appears to be mediated by specialized domains outside the TM region offers new support for the notion that the formation of stable dimers requires greater binding energies than can often be generated by the TM region alone, as also suggested elsewhere (Gurevich and Gurevich, 2008b). Interestingly, two of the *Rhodopsin*-family GPCR dimers identified here (LGR4 and LGR5) have large extracellular domains similar in structure to those of the *Frizzled* receptors. For the other *Rhodopsin*-family dimers, it is more likely that dimerization relies only on TM region contacts, as suggested here for S1P3, implying that these types of receptors, at best, interact only transiently.

Several of our observations would be explained by the rare, episodic appearance of *Rhodopsin*-family dimers during vertebrate evolution (Figure 5F): (1) that the dimers comprise a small fraction of GPCRs; (2) that they form small, closely related phylogenetic clusters; (3) that these receptors do not share ligand- or G protein selectivity outside the clusters; and (4) that closely related receptors can have different stoichiometries. The infrequency and distribution of the dimers seem to mirror a striking correlation between stoichiometry and GPCR family size also suggested by our data. Despite all appearing contemporaneously ~1.3 billion years ago, the dimeric *Frizzled* and *Glutamate* receptor families contain only 11 and 22 members, respectively, whereas there are now >700 predominantly monomeric *Rhodopsin*-family receptors (Nordstrom et al., 2011). Similarly, having split from the *Rhodopsin* family just ~300 million years ago (Nordstrom et al., 2011), it seems telling to us that there are >28 monomeric *Taste2* receptors and only three *Glutamate*-family *Taste1* dimers (Chandrashekar et al., 2000; Nelson et al., 2001), bearing in mind that both groups of receptors perform the same physiological function.

Why, then, are *Rhodopsin*-family GPCRs so dominant among extant vertebrates, and why are the numbers of putative dimers so low? Assuming that the root ancestor of the *Rhodopsin*-family GPCRs was monomeric, as our limited analysis of the *cAMP*-family GPCRs seems to imply, we suggest that at least three forces helped shape *Rhodopsin*-family receptor evolution. First, the functional autonomy of *Rhodopsin*-family monomers (e.g., Bayburt et al., 2007; Kuszak et al., 2009; Leitz et al., 2006; Whorton et al., 2007), allied with an intron-less mode of gene duplication that would have preserved this functionality, might have favored a classical birth-and-death mechanism of family expansion (Nei and Rooney, 2005). Second, the cylindrically arranged TM domains, which form deep pockets that bind mostly small ligands, could have allowed very fast functional diversification. Finally, dimerization might have increased the "fitness density" of receptors whose functions depend on dimerization, constraining the capacity of families of dimers, such as the *Frizzled* and *Glutamate* receptors, to diverge (Makino and Gojobori, 2006). This is because, following gene duplication, any "new" receptor would potentially interfere with the function of the "parent" receptor until the capacity for physical interactions was lost, as proposed for other receptor systems (e.g., Fraser et al., 2003; Makino and Gojobori, 2006). The paucity of *Rhodopsin*-family dimers could reflect the frequency of spontaneous gain-of-dimerization events if dimerization, per se, does not confer functionality.

## EXPERIMENTAL PROCEDURES

### Quantification of HEK293T Transcriptome

cDNA was generated by reverse transcription of total mRNA harvested from HEK293T cells and sequenced using high-throughput RNA-seq (Illumina). Individual sequences were mapped and quantified using TopHat and Cufflinks software (Center for Computational Biology, Johns Hopkins University). Output fragments were then assigned as encoding GPCRs by reference to the Universal Protein Resource (www.uniprot.org).

### BRET Vector Construction

GPCR genes were cloned into the pGFP$^2$ N3 (PerkinElmer), pRluc N3 (PerkinElmer), and pU (described in Felce et al., 2014) vectors for BRET experiments. Genes were amplified from cDNA generated from HEK293T cells by PCR using oligonucleotide primers binding the 5′ and 3′ sequences of either the open reading frame (ORF) or UTRs, or in two stages whereby the 5′ and 3′ halves of the ORF were amplified independently and then used as a template for generating a chimeric full-length product. All oligonucleotide primers used in this study and the cloning route adopted for each *Rhodopsin*-family GPCR gene are described in Data S1 ("R Primers" and "Non-R Primers"). In some instances, the full-length sequence of the gene of interest was synthesized directly (GeneArt, Invitrogen). cDNA encoding $\beta_1$AR was obtained from Robert Lefkowitz (Duke University) in the form of a FLAG-*ADRB1* pcDNA3 construct (Tang et al., 1999) from Addgene (plasmid #14698). This was used as a template for full-length PCR amplification as described earlier. Candidate *cAMP*-family receptors were synthesized directly using GeneArt and amplified using primers given in Data S1 ("Non-R Primers"). All amplified GPCR genes were cloned into the N3 BRET vectors by digestion at the flanking restriction endonuclease sites indicated in Data S1 ("R Primers" and "Non-R Primers"). The 3′ terminal restriction site used in most cases was BamHI, leading to encoded proteins with a linker sequence of GDPPVAT between the C terminus of the GPCR and the N-terminal sequence of GFP$^2$ or Rluc. Subsets of constructs contained linker sequences of GVPRARDPPVAT (12 receptors) or KLAVPRARDPPVAT (2 receptors), owing to differences in restriction site used; linker sequences are given in Data S1 ("R Primers" and "Non-R Primers"). The choice of linker seemed to have no bearing on observed receptor stoichiometry (Figures S4E and S4F).

FKBP-tagged CD86 and $\beta_2$AR constructs were prepared by amplifying FKBP cDNA from the pC$_4$-F$_V$1E vector (Ariad) using oligonucleotide primers 5′-TAGTAGGGATCCAGGAGTGCAGGTGGAAACCATC-3′ and 5′-CTACTAG GCTCCCCCTCCAGCTTCAGCAGCTCCACGTCG-3′. The amplified product was then ligated between CD86- or $\beta_2$AR-encoding cDNA and sequences encoding GFP$^2$ or Rluc in N3 BRET constructs previously described by James et al. (2006), using a BamHI restriction site.

### Chimeric Receptor Cloning

Chimeras of the *S1PR3/LPAR1* and *FZD10/TAS2R19* genes were generated using multiple overlapping PCR reactions using primers given in Data S1 ("Non-R Primers"). Full details are provided in the Supplemental Experimental Procedures.

### Confocal Microscopy

Surface expression of GPCRs fused to GFP was assessed using confocal microscopy. HEK293T cells were transfected 24 hr after plating onto microscope coverslips, with 1 μg pGFP$^2$-GPCR DNA per 6 × 10$^5$ cells, using GeneJuice (Novagen) as per the manufacturer's protocol. Cells were fixed using PBS/4% paraformaldehyde for 10 min. Coverslips were then mounted onto glass slides using VectaShield mounting medium (Vector Laboratories) and imaged using a Zeiss LSM-780 inverted confocal microscope under an oil-immersed 60× objective lens, with excitation laser light typically at 488 nm. Images were collected at the midpoint of the cell using a 515-nm ± 15-nm emission filter and manipulated minimally to improve the signal-to-noise ratio.

Receptors were assessed qualitatively for cellular distribution and assigned an expression category (Table S1). Protein aggregation was identified by the

presence of non-uniform accumulations of GFP fusion protein with fluorescence intensities and z-plane distributions greater than that expected of protein retained in internal membranes (Zhang et al., 2005). Typically, such aggregates would be the brightest objects in the field of view. Proteins with expression profiles assigned to either category F or category G were not progressed to pRluc and pU expression, or to subsequent BRET analysis.

### Type-1 and -3 BRET Assays

Type-1 and -3 BRET assays were performed on all GPCRs with adequate surface expression according to confocal microscopy. Type-1 BRET was performed as described previously (James et al., 2006) on HEK293T cells transiently transfected with BRET pairs of the target gene. Additional details are provided in the Supplemental Experimental Procedures. $BRET_{eff}$ and GFP:Rluc ratios were measured 24 hr post-transfection for the majority of receptors. Some GPCRs were insufficiently well expressed to give reliable data after 24 hr and so were, instead, assayed after 48 hr. GPCRs assayed after 48 hr were: $AT_1$, CCR11, GPER, NPY1R, OR4D1, and PAR1. Each receptor was tested in at least three independent experiments until a sufficiently broad range of GFP:Rluc ratios was assayed.

Type-3 BRET assays were performed as described previously (Felce et al., 2014) in HEK293T cells transiently transfected with BRET pairs of the target gene along with competitor or blank pU expression vector. In all instances, a 2:1 ratio of pU:($pGFP^2$+pRluc) was used to ensure an excess of competitor over labeled protein (an equivalent expression of proteins from $pGFP^2$, pRluc, and pU vectors was demonstrated previously; Felce et al., 2014), and a 12:1 $pGFP^2$:pRluc ratio was used to ensure measurable levels of BRET. In most cases this was achieved by transfecting $6 \times 10^5$ cells with 1 $\mu$g pU, 0.462 $\mu$g $pGFP^2$, and 0.038 $\mu$g pRluc. In cases of low receptor expression, these amounts were increased to 2 $\mu$g pU, 0.924 $\mu$g $pGFP^2$, and 0.076 $\mu$g pRluc. Increases in DNA were required for 5-$HT_{2B}$, $AT_1$, $B_2$, CCR11, EDNRA, GPER, NPY1R, OR4D1, OXER1, and PAR1. Data were collected from a minimum of three independent experiments.

For BRET analysis of FKBP-tagged inducible dimers, cells were incubated for 45 min at room temperature in the presence of various amounts of AP20187 inducer in PBS prior to the assay. Amounts of inducer required to achieve various levels of dimerization were calculated from the relationship between inducer concentration and $BRET_{eff}$ for $CD86_{FKBP}$ (Figure S2B). The required concentrations were: 0% dimerization, 0 nM; 10% dimerization, 35 nM; 20% dimerization, 85 nM; 30% dimerization, 145 nM; 40%, 225 nM; 50%, 335 nM; and 100%, 5 $\mu$M. The statistical methods used to analyze the BRET data are described in the Supplemental Experimental Procedures.

### Vector Construction for SMCCCD

C-terminally SNAP-tag- and HaloTag-labeled LPA1, S1P3, $\beta_1$AR, and $\alpha_{2C}$AR were expressed by subcloning the respective genes from $pGFP^2$ into pHRI-SNAP-tag and pHRI-HaloTag vectors, as described previously (Latty et al., 2015), using *MluI* and *NotI* restriction sites. In both pHRI vectors, expression is under the control of the ecdysone-dependent minimal promoter so that expression is limited to ~2,000–4,000 receptors per cell. CD86- and CD28-expressing vectors for SMCCCD were described previously (Latty et al., 2015). All receptors analyzed with SMCCCD contained a short Gly-Asp-Pro sequence between the C terminus of the receptor and the N terminus of either SNAP-tag or HaloTag.

### SMCCCD Analysis

CHO K1 cells were transfected with receptor-expressing constructs for SMCCCD analysis as described elsewhere (Latty et al., 2015). DNA ratios and post-transfection incubations required to achieve 100–1,000 HaloTag spots per cell and SnapTag:HaloTag ratios between 1:1 and 6:1 are indicated in Table S2. Receptor labeling, fixation, and data collection were performed as described previously (Latty et al., 2015).

## DATA AND SOFTWARE AVAILABILITY

The RNA-seq data described in this paper have the accession number GEO: GSE102461. The original experimental data are available at Mendeley Data, accession number http://dx.doi.org/10.17632/tb93gs9j29.1

## SUPPLEMENTAL INFORMATION

Supplemental Information includes Supplemental Experimental Procedures, five figures, three tables, and one data file and can be found with this article online at http://dx.doi.org/10.1016/j.celrep.2017.08.072.

## AUTHOR CONTRIBUTIONS

J.H.F., S.L.L., D.K., and S.J.D. designed the experiments. J.H.F., S.L.L., S.F.L., D.K., and S.J.D. wrote the manuscript. J.H.F. and R.G.K. cloned, expressed, and collected BRET data for all GPCRs. S.R.M. cloned, expressed, and collected BRET data for most *Frizzled* and *Taste2* GPCRs. J.H.F. analyzed the BRET data. Y.L. performed and analyzed the RNA-seq profiling. J.H.F. and S.L.L. performed the microscopy experiments and analyzed the data.

## ACKNOWLEDGMENTS

This work was supported by the Wellcome Trust (grant number 098274/Z/12/Z to S.J.D.), the UK Medical Research Council (G0901545 to S.J.D. and D.K.), and the Royal Society (RP150066 to D.K. and UF120277 to S.F.L.).

Received: April 11, 2017
Revised: July 14, 2017
Accepted: August 23, 2017
Published: September 12, 2017

## REFERENCES

AbdAlla, S., Lother, H., el Massiery, A., and Quitterer, U. (2001). Increased AT(1) receptor heterodimers in preeclampsia mediate enhanced angiotensin II responsiveness. Nat. Med. *7*, 1003–1009.

Angers, S., Salahpour, A., Joly, E., Hilairet, S., Chelsky, D., Dennis, M., and Bouvier, M. (2000). Detection of beta 2-adrenergic receptor dimerization in living cells using bioluminescence resonance energy transfer (BRET). Proc. Natl. Acad. Sci. USA *97*, 3684–3689.

Barak, L.S., Ferguson, S.S.G., Zhang, J., Martenson, C., Meyer, T., and Caron, M.G. (1997). Internal trafficking and surface mobility of a functionally intact beta2-adrenergic receptor-green fluorescent protein conjugate. Mol. Pharmacol. *51*, 177–184.

Bayburt, T.H., Leitz, A.J., Xie, G., Oprian, D.D., and Sligar, S.G. (2007). Transducin activation by nanoscale lipid bilayers containing one and two rhodopsins. J. Biol. Chem. *282*, 14875–14881.

Baycin-Hizal, D., Tabb, D.L., Chaerkady, R., Chen, L., Lewis, N.E., Nagarajan, H., Sarkaria, V., Kumar, A., Wolozny, D., Colao, J., et al. (2012). Proteomic analysis of Chinese hamster ovary cells. J. Proteome Res. *11*, 5265–5276.

Bouvier, M., and Hebert, T.E. (2014). CrossTalk proposal: Weighing the evidence for class A GPCR dimers, the evidence favours dimers. J. Physiol. *592*, 2439–2441.

Bouvier, M., Heveker, N., Jockers, R., Marullo, S., and Milligan, G. (2007). BRET analysis of GPCR oligomerization: newer does not mean better. Nat. Methods *4*, 3–4.

Brismar, H., Asghar, M., Carey, R.M., Greengard, P., and Aperia, A. (1998). Dopamine induced recruitment of dopamine D1 receptors to the plasma membrane. Proc. Natl. Acad. Sci. USA *95*, 5573–5578.

Cai, X., Bai, B., Zhang, R., Wang, C., and Chen, J. (2017). Apelin receptor homodimer-oligomers revealed by single-molecule imaging and novel G protein-dependent signaling. Sci. Rep. *7*, 40335.

Calebiro, D., Rieken, F., Wagner, J., Sungkaworn, T., Zabel, U., Borzi, A., Cocucci, E., Zuern, A., and Lohse, M.J. (2013). Single-molecule analysis of fluorescently labeled G-protein-coupled receptors reveals complexes with distinct dynamics and organization. Proc. Natl. Acad. Sci. USA *110*, 743–748.

Chabre, M., and le Maire, M. (2005). Monomeric G-protein-coupled receptor as a functional unit. Biochemistry *44*, 9395–9403.

Chabre, M., Deterre, P., and Antonny, B. (2009). The apparent cooperativity of some GPCRs does not necessarily imply dimerization. Trends Pharmacol. Sci. *30*, 182–187.

Chandrashekar, J., Mueller, K.L., Hoon, M.A., Adler, E., Feng, L.X., Guo, W., Zuker, C.S., and Ryba, N.J.P. (2000). T2Rs function as bitter taste receptors. Cell *100*, 703–711.

Contento, R.L., Molon, B., Boularan, C., Pozzan, T., Manes, S., Marullo, S., and Viola, A. (2008). CXCR4-CCR5: A couple modulating T cell functions. Proc. Natl. Acad. Sci. USA *105*, 10101–10106.

Deupi, X., and Standfuss, J. (2011). Structural insights into agonist-induced activation of G-protein-coupled receptors. Curr. Opin. Struct. Biol. *21*, 541–551.

Ernst, O.P., Gramse, V., Kolbe, M., Hofmann, K.P., and Heck, M. (2007). Monomeric G protein-coupled receptor rhodopsin in solution activates its G protein transducin at the diffusion limit. Proc. Natl. Acad. Sci. USA *104*, 10859–10864.

Felce, J.H., and Davis, S.J. (2012). Unraveling receptor stoichiometry using bret. Front. Endocrinol. *3*, 86–86.

Felce, J.H., Knox, R.G., and Davis, S.J. (2014). Type-3 BRET, an improved competition-based bioluminescence resonance energy transfer assay. Biophys. J. *106*, L41–L43.

Fraser, H.B., Wall, D.P., and Hirsh, A.E. (2003). A simple dependence between protein evolution rate and the number of protein-protein interactions. BMC Evol. Biol. *3*, 11.

Fredriksson, R., and Schioth, H.B. (2005). The repertoire of G-protein-coupled receptors in fully sequenced genomes. Mol. Pharmacol. *67*, 1414–1425.

Fredriksson, R., Lagerstrom, M.C., Lundin, L.G., and Schioth, H.B. (2003). The G-protein-coupled receptors in the human genome form five main families. Phylogenetic analysis, paralogon groups, and fingerprints. Mol. Pharmacol. *63*, 1256–1272.

Gurevich, V.V., and Gurevich, E.V. (2008a). GPCR monomers and oligomers: it takes all kinds. Trends Neurosci. *31*, 74–81.

Gurevich, V.V., and Gurevich, E.V. (2008b). How and why do GPCRs dimerize? Trends Pharmacol. Sci. *29*, 234–240.

Hegener, O., Prenner, L., Runkel, F., Baader, S.L., Kappler, J., and Häberlein, H. (2004). Dynamics of beta2-adrenergic receptor-ligand complexes on living cells. Biochemistry *43*, 6190–6199.

Hein, L., Ishii, K., Coughlin, S.R., and Kobilka, B.K. (1994). Intracellular targeting and trafficking of thrombin receptors. A novel mechanism for resensitization of a G protein-coupled receptor. J. Biol. Chem. *269*, 27719–27726.

Hern, J.A., Baig, A.H., Mashanov, G.I., Birdsall, B., Corrie, J.E.T., Lazareno, S., Molloy, J.E., and Birdsall, N.J.M. (2010). Formation and dissociation of M-1 muscarinic receptor dimers seen by total internal reflection fluorescence imaging of single molecules. Proc. Natl. Acad. Sci. USA *107*, 2693–2698.

Huang, J., Chen, S., Zhang, J.J., and Huang, X.-Y. (2013). Crystal structure of oligomeric beta(1)-adrenergic G protein-coupled receptors in ligand-free basal state. Nat. Struct. Mol. Biol. *20*, 419.

James, J.R., and Davis, S.J. (2007). Reply to: experimental challenge to a ‘rigorous’ BRET analysis of GPCR oligomerization. Nat. Methods *4*, 601.

James, J.R., Oliveira, M.I., Carmo, A.M., Iaboni, A., and Davis, S.J. (2006). A rigorous experimental framework for detecting protein oligomerization using bioluminescence resonance energy transfer. Nat. Methods *3*, 1001–1006.

Jonas, K.C., Fanelli, F., Huhtaniemi, I.T., and Hanyaloglu, A.C. (2015). Single molecule analysis of functionally asymmetric G protein-coupled receptor (GPCR) oligomers reveals diverse spatial and structural assemblies. J. Biol. Chem. *290*, 3875–3892.

Kasai, R.S., Suzuki, K.G.N., Prossnitz, E.R., Koyama-Honda, I., Nakada, C., Fujiwara, T.K., and Kusumi, A. (2011). Full characterization of GPCR monomer-dimer dynamic equilibrium by single molecule imaging. J. Cell Biol. *192*, 463–480.

Katritch, V., Cherezov, V., and Stevens, R.C. (2012). Diversity and modularity of G protein-coupled receptor structures. Trends Pharmacol. Sci. *33*, 17–27.

Kim, U., Wooding, S., Ricci, D., Jorde, L.B., and Drayna, D. (2005). Worldwide haplotype diversity and coding sequence variation at human bitter taste receptor loci. Hum. Mutat. *26*, 199–204.

Kuszak, A.J., Pitchiaya, S., Anand, J.P., Mosberg, H.I., Walter, N.G., and Sunahara, R.K. (2009). Purification and functional reconstitution of monomeric mu-opioid receptors: allosteric modulation of agonist binding by Gi2. J. Biol. Chem. *284*, 26732–26741.

Lambert, N.A., and Javitch, J.A. (2014). CrossTalk opposing view: weighing the evidence for class A GPCR dimers, the jury is still out. J. Physiol. *592*, 2443–2445.

Latty, S.L., Felce, J.H., Weimann, L., Lee, S.F., Davis, S.J., and Klenerman, D. (2015). Referenced single-molecule measurements differentiate between GPCR oligomerization states. Biophys. J. *109*, 1798–1806.

Leitz, A.J., Bayburt, T.H., Barnakov, A.N., Springer, B.A., and Sligar, S.G. (2006). Functional reconstitution of $\beta_2$-adrenergic receptors utilizing self-assembling Nanodisc technology. Biotechniques *40*, 601–612.

Makino, T., and Gojobori, T. (2006). The evolutionary rate of a protein is influenced by features of the interacting partners. Mol. Biol. Evol. *23*, 784–789.

Manglik, A., Kruse, A.C., Kobilka, T.S., Thian, F.S., Mathiesen, J.M., Sunahara, R.K., Pardo, L., Weis, W.I., Kobilka, B.K., and Granier, S. (2012). Crystal structure of the μ-opioid receptor bound to a morphinan antagonist. Nature *485*, 321–326.

Mercier, J.F., Salahpour, A., Angers, P., Breit, A., and Bouvier, M. (2002). Quantitative assessment of beta 1- and beta 2-adrenergic receptor homo- and heterodimerization by bioluminescence resonance energy transfer. J. Biol. Chem. *277*, 44925–44931.

Nei, M., and Rooney, A.P. (2005). Concerted and birth-and-death evolution of multigene families. Annu. Rev. Genet. *39*, 121–152.

Nelson, G., Hoon, M.A., Chandrashekar, J., Zhang, Y.F., Ryba, N.J.P., and Zuker, C.S. (2001). Mammalian sweet taste receptors. Cell *106*, 381–390.

Nenasheva, T.A., Neary, M., Mashanov, G.I., Birdsall, N.J.M., Breckenridge, R.A., and Molloy, J.E. (2013). Abundance, distribution, mobility and oligomeric state of M-2 muscarinic acetylcholine receptors in live cardiac muscle. J. Mol. Cell. Cardiol. *57*, 129–136.

Nordstrom, K.J.V., Almen, M.S., Edstam, M.M., Fredriksson, R., and Schioth, H.B. (2011). Independent HHsearch, Needleman-Wunsch-based, and motif analyses reveal the overall hierarchy for most of the G protein-coupled receptor families. Mol. Biol. Evol. *28*, 2471–2480.

Pétrin, D., and Hebert, T.E. (2012). The functional size of GPCRs - monomers, dimers or tetramers? Subcell. Biochem. *63*, 67–81.

Pfleger, K.D.G., and Eidne, K.A. (2005). Monitoring the formation of dynamic G-protein-coupled receptor-protein complexes in living cells. Biochem. J. *385*, 625–637.

Ramsay, D., Kellett, E., McVey, M., Rees, S., and Milligan, G. (2002). Homo- and hetero-oligomeric interactions between G-protein-coupled receptors in living cells monitored by two variants of bioluminescence resonance energy transfer (BRET): hetero-oligomers between receptor subtypes form more efficiently than between less closely related sequences. Biochem. J. *365*, 429–440.

Rasmussen, S.G.F., DeVree, B.T., Zou, Y., Kruse, A.C., Chung, K.Y., Kobilka, T.S., Thian, F.S., Chae, P.S., Pardon, E., Calinski, D., et al. (2011). Crystal structure of the β2 adrenergic receptor-Gs protein complex. Nature *477*, 549–555.

Salahpour, A., and Masri, B. (2007). Experimental challenge to a ‘rigorous’ BRET analysis of GPCR oligomerization. Nat. Methods *4*, 599–600.

Salom, D., Lodowski, D.T., Stenkamp, R.E., Le Trong, I., Golczak, M., Jastrzebska, B., Harris, T., Ballesteros, J.A., and Palczewski, K. (2006). Crystal structure of a photoactivated deprotonated intermediate of rhodopsin. Proc. Natl. Acad. Sci. USA *103*, 16123–16128.

Tang, Y.T., Hu, L.Y.A., Miller, W.E., Ringstad, N., Hall, R.A., Pitcher, J.A., DeCamilli, P., and Lefkowitz, R.J. (1999). Identification of the endophilins (SH3p4/p8/p13) as novel binding partners for the beta 1-adrenergic receptor. Proc. Natl. Acad. Sci. USA *96*, 12559–12564.

Tubio, M.R., Fernandez, N., Fitzsimons, C.P., Copsel, S., Santiago, S., Shayo, C., Davio, C., and Monczor, F. (2010). Expression of a G protein-coupled receptor (GPCR) leads to attenuation of signaling by other GPCRs: experimental

evidence for a spontaneous GPCR constitutive inactive form. J. Biol. Chem. *285*, 14990–14998.

Venkatakrishnan, A.J., Deupi, X., Lebon, G., Tate, C.G., Schertler, G.F., and Babu, M.M. (2013). Molecular signatures of G-protein-coupled receptors. Nature *494*, 185–194.

Whorton, M.R., Bokoch, M.P., Rasmussen, S.G.F., Huang, B., Zare, R.N., Kobilka, B., and Sunahara, R.K. (2007). A monomeric G protein-coupled receptor isolated in a high-density lipoprotein particle efficiently activates its G protein. Proc. Natl. Acad. Sci. USA *104*, 7682–7687.

Wu, B.L., Chien, E.Y.T., Mol, C.D., Fenalti, G., Liu, W., Katritch, V., Abagyan, R., Brooun, A., Wells, P., Bi, F.C., et al. (2010). Structures of the CXCR4 chemokine GPCR with small-molecule and cyclic peptide antagonists. Science *330*, 1066–1071.

Zhang, X.Q., Smith, D.L., Merlin, A.B., Engemann, S., Russel, D.E., Roark, M., Washington, S.L., Maxwell, M.M., Marsh, J.L., Thompson, L.M., et al. (2005). A potent small molecule inhibits polyglutamine aggregation in Huntington's disease neurons and suppresses neurodegeneration in vivo. Proc. Natl. Acad. Sci. USA *102*, 892–897.

Cell Reports, Volume *20*

## Supplemental Information

# Receptor Quaternary Organization Explains

# G Protein-Coupled Receptor Family Structure

**James H. Felce, Sarah L. Latty, Rachel G. Knox, Susan R. Mattick, Yuan Lui, Steven F. Lee, David Klenerman, and Simon J. Davis**

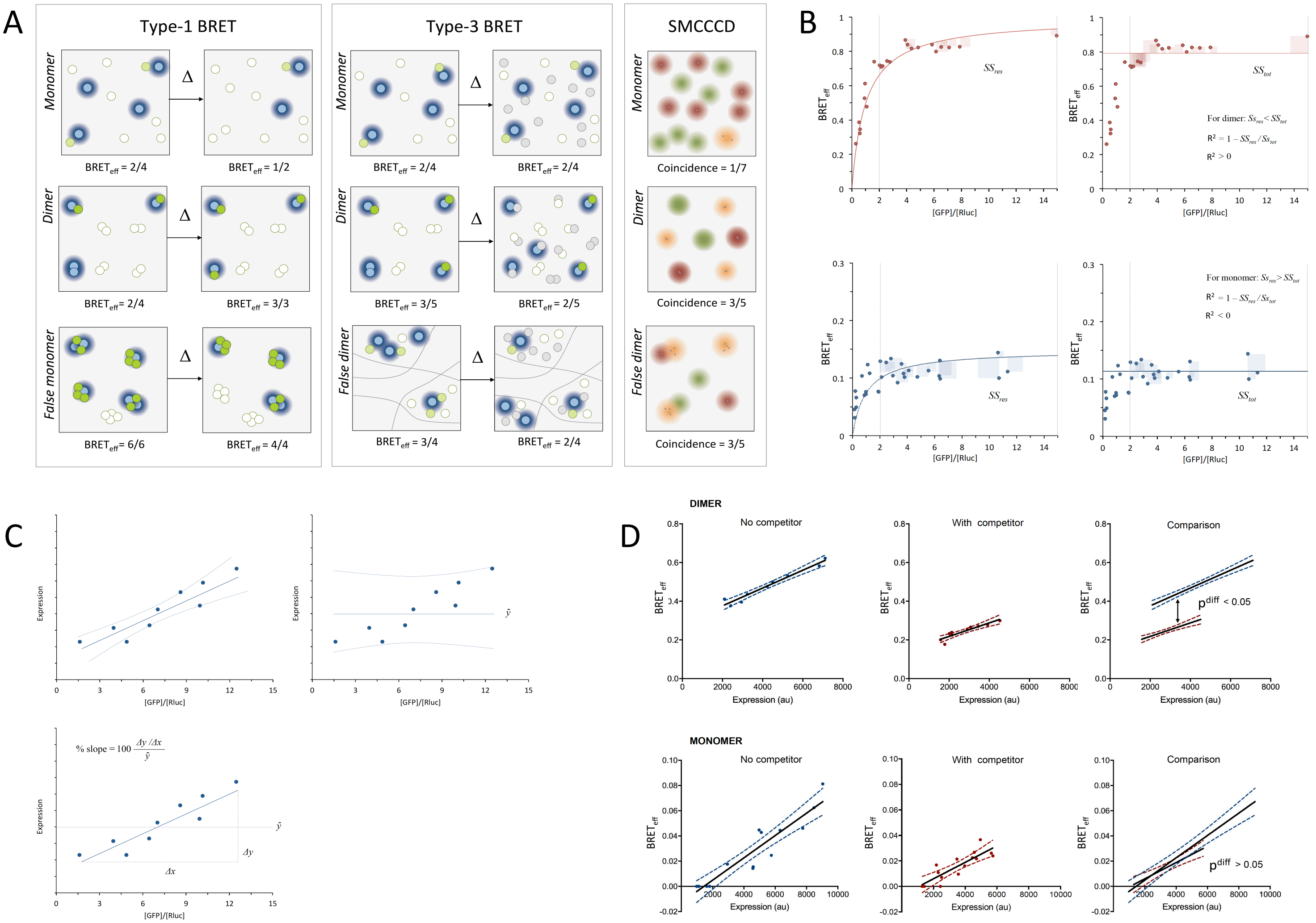
A
Type-1 BRET
Type-3 BRET
SMCCCD
Monomer
Dimer
False monomer
False dimer
Δ
BRETeff = 2/4
BRETeff = 1/2
BRETeff = 2/4
BRETeff = 3/3
BRETeff = 6/6
BRETeff = 4/4
BRETeff = 2/4
BRETeff = 2/4
BRETeff = 3/5
BRETeff = 2/5
BRETeff = 3/4
BRETeff = 2/4
Coincidence = 1/7
Coincidence = 3/5
Coincidence = 3/5
B
BRETeff
[GFP]/[Rluc]
SSres
SStot
For dimer: SSres < SStot
R² = 1 − SSres / SStot
R² > 0
For monomer: SSres > SStot
R² = 1 − SSres / SStot
R² < 0
C
Expression
[GFP]/[Rluc]
ȳ
% slope = 100 (Δy / Δx) / ȳ
Δy
Δx
D
DIMER
No competitor
With competitor
Comparison
BRETeff
Expression (au)
pdiff < 0.05
MONOMER
pdiff > 0.05

**Figure S1. Explanations of Assay Principles and Statistical Analyses, Related to Figure 1.**
(A) Comparisons of outcomes and limitations of type-1 and -3 BRET assays and SMCCCD. BRET donors are represented as blue circles with halos of the RET-permissive radius; BRET acceptors as white (non-fluorescing) or green (fluorescing) circles. SMCCCD-imaged fluorophores are shown as individual color (red and green) or combined color (orange) diffraction-radius spots surrounding the tagged protein (gray). In the type-1 BRET assay, monomers exhibit no change in $BRET_{eff}$ as acceptor:donor ratio increases because the replacement of donors with acceptors does not impact on the availability of acceptors for the remaining donors (left, top). Conversely, as acceptor:donor ratio increases for dimers $BRET_{eff}$ will increase as fewer donor-donor pairs remain (left, middle). False monomer results can be produced in the case of high-order oligomers as $BRET_{eff}$ is also largely unaffected by increases in acceptor:donor ratio (left, bottom). In the type-3 BRET assay, monomers exhibit no change in $BRET_{eff}$ when untagged competitor proteins are introduced into the system (center, top), whereas $BRET_{eff}$ for dimers will decrease due to disruption of productive donor-acceptor dimers (center, middle). False dimers can be produced in the case of monomers that undergo clustering within the membrane that becomes more relaxed upon introduction of competitors. This causes a reduction in non-specific $BRET_{eff}$ due to the reduced effective concentration of tagged proteins (center, bottom). In SMCCCD, tagged proteins are detected as diffraction-limited spots with all proteins within the diffraction-limited area identified as a single spot. Proteins are tagged and imaged in two colors, allowing two or more proteins within the diffraction-limited spot (*i.e.* 'coincident' signals) to be identified. For monomers (right, top), apparent coincidence is the product only of by-chance co-localization within the diffraction-limited spots. Coincidence is higher for dimers (right, middle) because *bona fide* interaction results in up to 50% (*i.e.* 25% green-green, 25% red-red, 50% green-red) of receptors being co-localized within the diffraction-limited spots. SMCCCD cannot distinguish between genuine dimers and clusters of monomers (right, bottom), which are observed as false dimers.
(B) Graphical explanation of type-1 BRET statistical analysis. For all type-1 BRET assays, data were fitted to dimer (left) and monomer (right) models. Fits were generated only for [GFP]/[Rluc] values between 2 and 15. The dimer models fitted data to Equation 1, while the monomer model fitted to a constant $BRET_{eff}$ across all [GFP]/[Rluc] values (i.e. $BRET_{eff} = BRET_{max}$). In both cases non-linear least-squares regression was used to generate optimal fits. To determine which fit better suited the data, the $R^2$ value was determined. This compares the residual sum of squares for the dimer model fit ($SS_{res}$) to that of a flat line (*i.e.* the monomer model; $SS_{tot}$). $R^2$ is determined as $1 - SS_{res} / SS_{tot}$. For a dimer, *e.g.* CXCR4 (top), $SS_{res}$ is smaller than $SS_{tot}$, so $R^2$ is positive. For a monomer, *e.g.* CCR6 (bottom), $SS_{res}$ is larger than $SS_{tot}$, so $R^2$ is negative.
(C) Explanation of $p$ and slope metrics for expression *vs* [GFP]/[Rluc] in type-1 BRET assays. The type-1 assay relies on overall expression being constant as acceptor:donor ratio increases. The probability, $p$, that total protein expression varied systematically with [GFP]/[Rluc] was tested by comparing the goodness-of-fit of a least-squares linear regression fit of the data (left, top) to that of a zero slope fit around mean expression (right, top) using a Fisher test. If the linear regression is significantly non-zero in its slope then the resulting $p$ value is <0.05. $P$ values for all type-1 BRET assays are provided in the Supplementary Data ("BRET Experiments"), along with mean percentage slope for all experiments. This was calculated as the slope exhibited by the linear regression fit expressed as a percentage of the mean expression value for all points (bottom); *i.e.*, if the percentage slope is 5.00, the total expression would increase 65% across the active range of 2-15 [GFP]/[Rluc].
(D) Graphical explanation of type-3 BRET statistical analysis. For all type-3 BRET assays, both datasets (*i.e.* with and without competitor) were fitted to a least-squares linear regression model (black line; dotted lines are 95% confidence limits of the fit). All points were used in the generation of the fit. The difference in the elevation of the two fits was then tested using a $t$ test to determine the probability that the two fits came from samples with identical $t$ distributions. A significant difference in the linear regression models resulted in $p^{diff}$ value below 0.05, whereas a $p^{diff}$ over 0.05 indicated no significant difference. The existence of a significant difference between datasets indicated the presence of dimers (*e.g.* CXCR4; top), whereas its absence suggested monomeric behavior (*e.g.* CCR6; bottom).

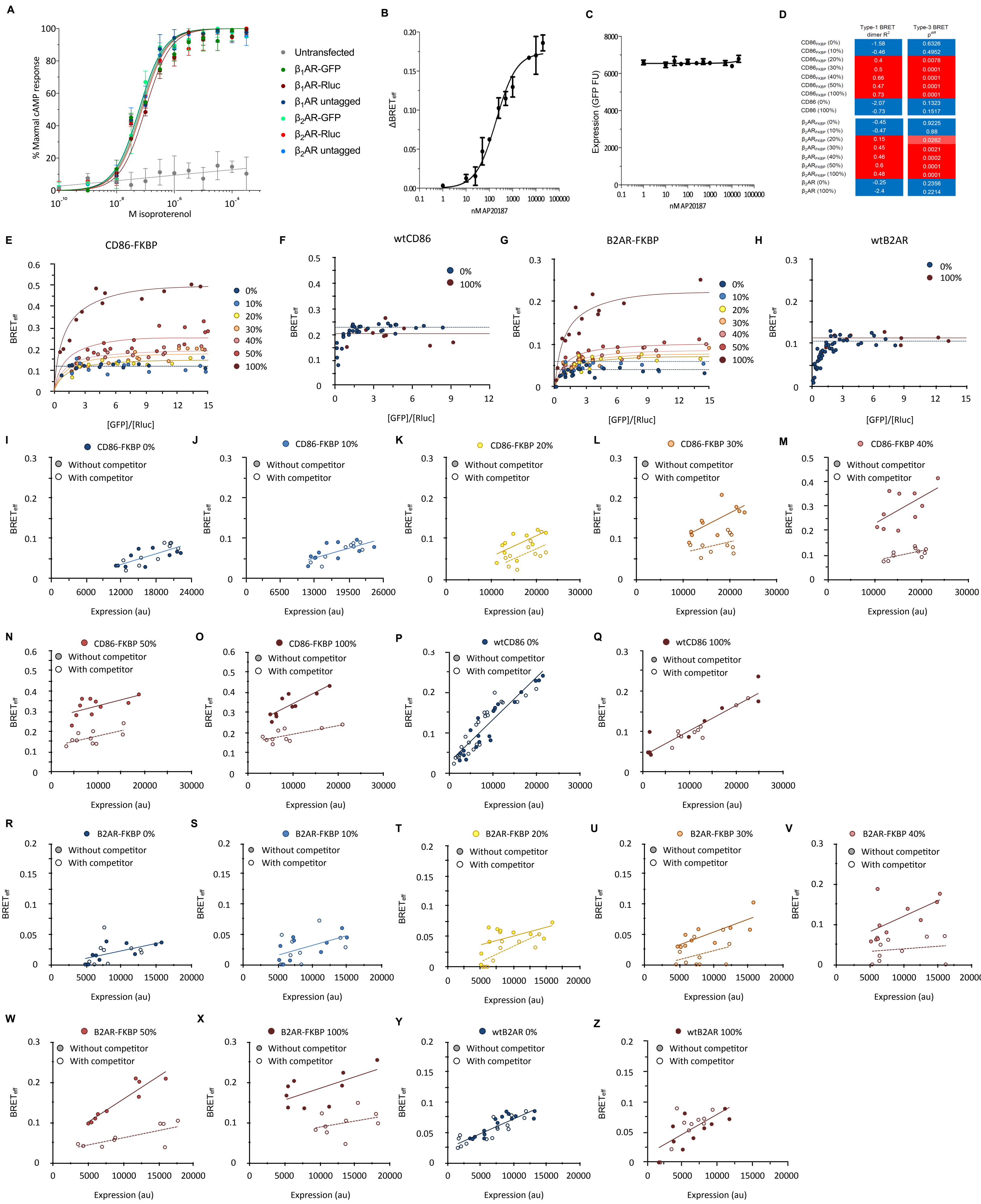
A
% Maxmal cAMP response
M isoproterenol
Untransfected
β1AR-GFP
β1AR-Rluc
β1AR untagged
β2AR-GFP
β2AR-Rluc
β2AR untagged
B
ΔBRETeff
nM AP20187
C
Expression (GFP FU)
nM AP20187
D
Type-1 BRET dimer R2
Type-3 BRET pdiff
CD86FKBP (0%) -1.58 0.6326
CD86FKBP (10%) -0.46 0.4952
CD86FKBP (20%) 0.4 0.0078
CD86FKBP (30%) 0.5 0.0001
CD86FKBP (40%) 0.66 0.0001
CD86FKBP (50%) 0.47 0.0001
CD86FKBP (100%) 0.73 0.0001
CD86 (0%) -2.07 0.1323
CD86 (100%) -0.73 0.1517
β2ARFKBP (0%) -0.45 0.9225
β2ARFKBP (10%) -0.47 0.88
β2ARFKBP (20%) 0.15 0.0262
β2ARFKBP (30%) 0.45 0.0021
β2ARFKBP (40%) 0.46 0.0002
β2ARFKBP (50%) 0.6 0.0001
β2ARFKBP (100%) 0.48 0.0001
β2AR (0%) -0.25 0.2356
β2AR (100%) -2.4 0.2214
E CD86-FKBP
F wtCD86
G B2AR-FKBP
H wtB2AR
BRETeff
[GFP]/[Rluc]
0%
10%
20%
30%
40%
50%
100%
I CD86-FKBP 0%
J CD86-FKBP 10%
K CD86-FKBP 20%
L CD86-FKBP 30%
M CD86-FKBP 40%
N CD86-FKBP 50%
O CD86-FKBP 100%
P wtCD86 0%
Q wtCD86 100%
R B2AR-FKBP 0%
S B2AR-FKBP 10%
T B2AR-FKBP 20%
U B2AR-FKBP 30%
V B2AR-FKBP 40%
W B2AR-FKBP 50%
X B2AR-FKBP 100%
Y wtB2AR 0%
Z wtB2AR 100%
Without competitor
With competitor
Expression (au)

**Figure S2 The Type-1 and Type-3 BRET Assays Readily Detect Dimers Corresponding to Only 20% of the Tagged Receptor Population, Related to Figure 1.**

(A) cAMP responses of untransfected CHO-K1 cells and CHO-K1 cells transiently transfected with untagged, GFP-, and Rluc-tagged versions of human $\beta_1$AR and $\beta_2$AR, as measured using the GloSensor cAMP assay. Data are expressed as % maximal response in each dataset, apart from the untransfected control, which are normalized to data for untagged $\beta_2$AR. Error bars indicate mean ± SE.

(B) Change in $BRET_{eff}$ between $CD86_{FKBP}GFP^2$ and $CD86_{FKBP}$Rluc in the presence of increasing concentrations of AP20187. AP20187 concentrations required to achieve various levels of dimerization were derived from these data. Error bars indicate mean ± SE.

(C) Total $CD86_{FKBP}GFP^2$ fluorescence at increasing AP20187 concentrations. Fluorescence is constant across concentrations, indicating AP20187 does not induce internalization and degradation of $CD86_{FKBP}$. The type-1 and type-3 BRET assays can detect dimerization of $CD86_{FKBP}$ and $\beta_2AR_{FKBP}$ at AP20187 concentrations sufficient to induce 20% dimerization. Error bars indicate mean ± SE.

(D) Color-coded summary of type-1 and -3 BRET assay outcomes for inducible dimers and wild-type controls at concentrations of AP20187 sufficient to induce various degrees of dimerization. Color coding is the same as in Figure 1E, ranging from monomeric (blue) to dimeric (red).

(E) Type-1 BRET data for $CD86_{FKBP}$ collected at various levels of induced dimerization. $CD86_{FKBP}$ demonstrates detectably dimeric behavior in the type-1 assay at AP20187 concentrations sufficient to induce 20% or more dimerization.

(F) Type-1 BRET data for wtCD86 collected without (0% dimerization) and with 5 μM AP20187 (100% dimerization). wtCD86 exhibits monomeric behavior even at an AP20187 concentration sufficient to induce 100% dimerization of FKBP-tagged equivalents.

(G) Type-1 BRET data for $\beta_2AR_{FKBP}$ collected at various levels of induced dimerization. $\beta_2AR_{FKBP}$ demonstrates detectably dimeric behavior in the type-1 assay at AP20187 concentrations sufficient to induce 20% or more dimerization.

(H) Type-1 BRET data for wt$\beta_2$AR collected without (0% dimerization) and with 5 μM AP20187 (100% dimerization). wt$\beta_2$AR exhibits monomeric behavior even at an AP20187 concentration sufficient to induce 100% dimerization of FKBP-tagged equivalents.

(I) Type-3 BRET data for $CD86_{FKBP}$ without (0% dimerization) AP20187. Data are consistent with monomeric behavior.

(J) Type-3 BRET data for $CD86_{FKBP}$ in the presence of 35 nM (10% dimerization) AP20187. Data are consistent with monomeric behavior.

(K) Type-3 BRET data for $CD86_{FKBP}$ in the presence of 85 nM (20% dimerization) AP20187. Data are consistent with dimeric behavior.

(L) Type-3 BRET data for $CD86_{FKBP}$ in the presence of 145 nM (30% dimerization) AP20187. Data are consistent with dimeric behavior.

(M) Type-3 BRET data for $CD86_{FKBP}$ in the presence of 225 nM (40% dimerization) AP20187. Data are consistent with dimeric behavior.

(N) Type-3 BRET data for $CD86_{FKBP}$ in the presence of 335 nM (50% dimerization) AP20187. Data are consistent with dimeric behavior.

(O) Type-3 BRET data for $CD86_{FKBP}$ in the presence of 5 μM (100% dimerization) AP20187. Data are consistent with dimeric behavior.

(P) Type-3 BRET data for wtCD86 without (0% dimerization) AP20187. Data are consistent with monomeric behavior.

(Q) Type-3 BRET data for wtCD86 in the presence of 5 μM (100% dimerization) AP20187. Data are consistent with monomeric behavior.

(R) Type-3 BRET data for $\beta_2AR_{FKBP}$ without (0% dimerization) AP20187. Data are consistent with monomeric behavior.

(S) Type-3 BRET data for $\beta_2AR_{FKBP}$ in the presence of 35 nM (10% dimerization) AP20187. Data are consistent with monomeric behavior.

(T) Type-3 BRET data for $\beta_2AR_{FKBP}$ in the presence of 85 nM (20% dimerization) AP20187. Data are consistent with dimeric behavior.

(U) Type-3 BRET data for $\beta_2AR_{FKBP}$ in the presence of 145 nM (30% dimerization) AP20187. Data are consistent with dimeric behavior.

(V) Type-3 BRET data for $\beta_2AR_{FKBP}$ in the presence of 225 nM (40% dimerization) AP20187. Data are consistent with dimeric behavior.

(W) Type-3 BRET data for $\beta_2AR_{FKBP}$ in the presence of 335 nM (50% dimerization) AP20187. Data are consistent with dimeric behavior.

(X) Type-3 BRET data for $\beta_2AR_{FKBP}$ in the presence of 5 μM (100% dimerization) AP20187. Data are consistent with dimeric behavior.

(Y) Type-3 BRET data for wt$\beta_2$AR without (0% dimerization) AP20187. Data are consistent with monomeric behavior.

(Z) Type-3 BRET data for wt$\beta_2$AR in the presence of 5 μM (100% dimerization) AP20187. Data are consistent with monomeric behavior.

See also Supplementary Data ("BRET Experiments").

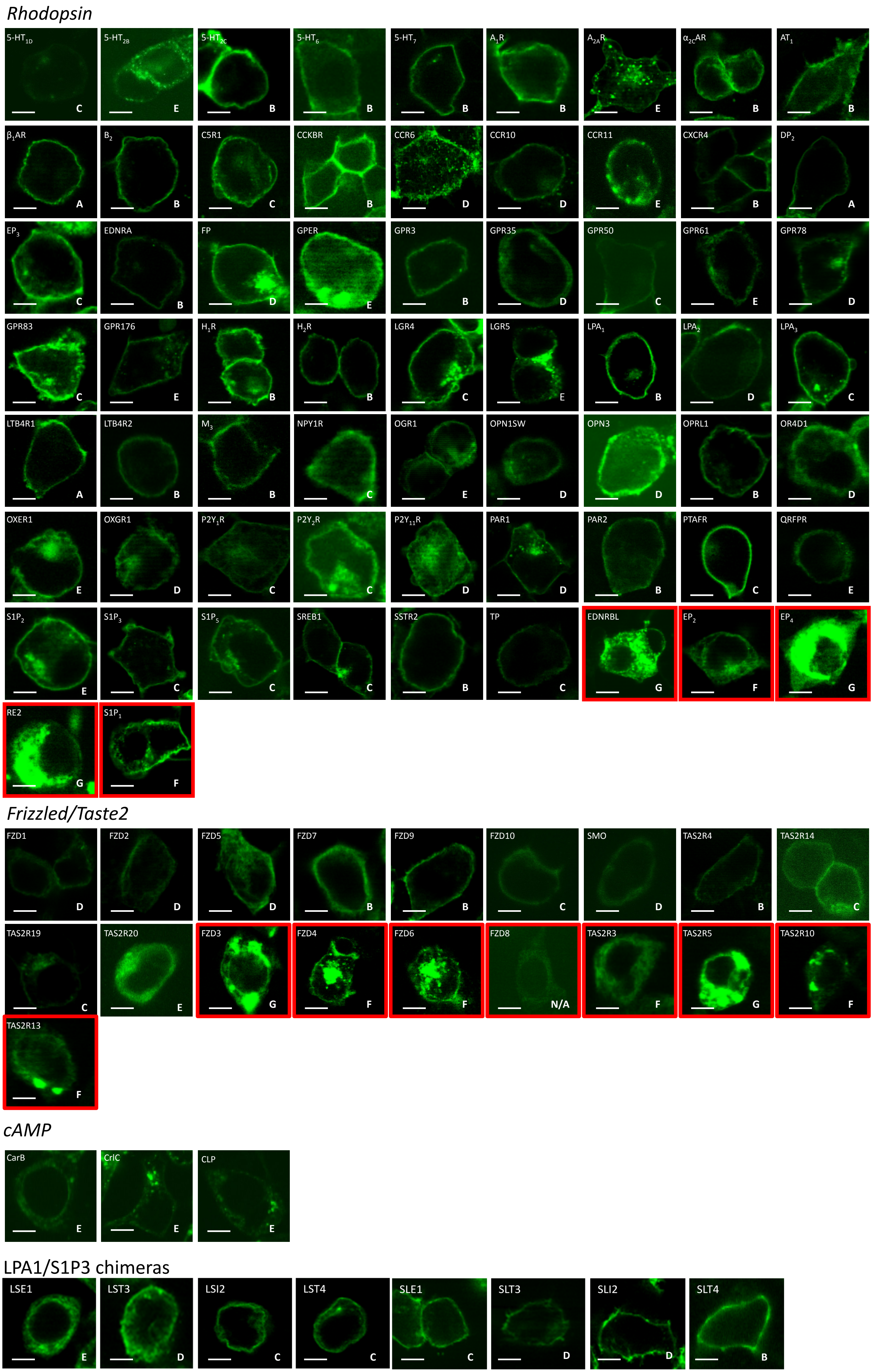

Rhodopsin
5-HT1D C
5-HT2B E
5-HT2C B
5-HT6 B
5-HT7 B
A1R B
A2AR E
α2CAR B
AT1 B
β1AR A
B2 B
C5R1 C
CCKBR B
CCR6 D
CCR10 D
CCR11 E
CXCR4 B
DP2 A
EP3 C
EDNRA B
FP D
GPER E
GPR3 B
GPR35 D
GPR50 C
GPR61 E
GPR78 D
GPR83 C
GPR176 E
H1R B
H2R B
LGR4 C
LGR5 E
LPA1 B
LPA2 D
LPA3 C
LTB4R1 A
LTB4R2 B
M3 B
NPY1R C
OGR1 E
OPN1SW D
OPN3 D
OPRL1 B
OR4D1 D
OXER1 E
OXGR1 D
P2Y1R C
P2Y2R C
P2Y11R D
PAR1 D
PAR2 B
PTAFR C
QRFPR E
S1P2 E
S1P3 C
S1P5 C
SREB1 C
SSTR2 B
TP C
EDNRBL G
EP2 F
EP4 G
RE2 G
S1P1 F
Frizzled/Taste2
FZD1 D
FZD2 D
FZD5 D
FZD7 B
FZD9 B
FZD10 C
SMO D
TAS2R4 B
TAS2R14 C
TAS2R19 C
TAS2R20 E
FZD3 G
FZD4 F
FZD6 F
FZD8 N/A
TAS2R3 F
TAS2R5 G
TAS2R10 F
TAS2R13 F
cAMP
CarB E
CrlC E
CLP E
LPA1/S1P3 chimeras
LSE1 E
LST3 D
LSI2 C
LST4 C
SLE1 C
SLT3 D
SLI2 D
SLT4 B

**Figure S3 Representative Confocal Microscopy Images of GPCR-GFP Constructs Expressed in HEK293T Cells, Related to Figure 1.**

Receptors were placed into categories A-G (Table S1) based on their subcellular localization and degree of observable GFP aggregation. Receptors identified as categories F and G (red borders) were not studied. FZD8 expressed too weakly for reliable assessment of localization, and was also not studied. Scale bars are 5 µm.

See also Table S1.

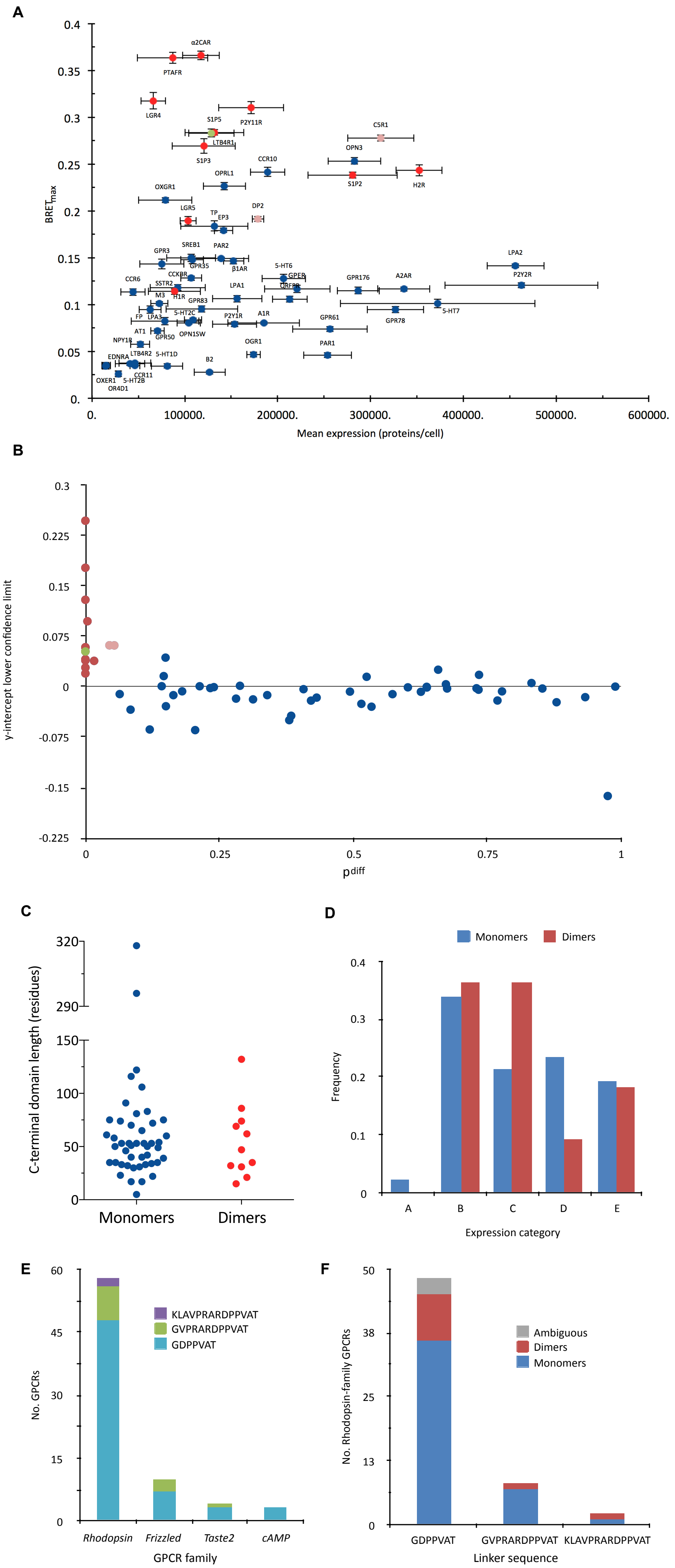

A
0.4
0.35
0.3
0.25
0.2
0.15
0.1
0.05
0.
$BRET_{max}$
α2CAR
PTAFR
LGR4
P2Y11R
S1P5
LTB4R1
S1P3
C5R1
OPN3
CCR10
OPRL1
S1P2
H2R
OXGR1
LGR5
TP
EP3
DP2
GPR3
SREB1
PAR2
GPR35
β1AR
5-HT6
GPER
QRFPR
CCKBR
SSTR2
H1R
CCR6
M3
GPR83
LPA1
GPR176
A2AR
LPA2
P2Y2R
5-HT7
GPR78
FP
LPA3
5-HT2C
P2Y1R
A1R
GPR61
AT1
GPR50
OPN1SW
NPY1R
OGR1
PAR1
EDNRA
LTB4R2
5-HT1D
B2
OXER1
5-HT2B
CCR11
OR4D1
0.
100000.
200000.
300000.
400000.
500000.
600000.
Mean expression (proteins/cell)
B
0.3
0.225
0.15
0.075
0
-0.075
-0.15
-0.225
y-intercept lower confidence limit
0
0.25
0.5
0.75
1
$p^{diff}$
C
320
290
150
100
50
0
C-terminal domain length (residues)
Monomers
Dimers
D
Monomers
Dimers
0.4
0.3
0.2
0.1
0
Frequency
A
B
C
D
E
Expression category
E
60
45
30
15
0
No. GPCRs
KLAVPRARDPPVAT
GVPRARDPPVAT
GDPPVAT
Rhodopsin
Frizzled
Taste2
cAMP
GPCR family
F
50
38
25
13
0
No. Rhodopsin-family GPCRs
Ambiguous
Dimers
Monomers
GDPPVAT
GVPRARDPPVAT
KLAVPRARDPPVAT
Linker sequence

**Figure S4 BRET$_{max}$, *y*-Intercept, C-Terminal Domain Length, and Expression Category for the *Rhodopsin*-Family GPCRs studied, Related to Figure 1.**

(A) Relationship between BRET$_{max}$ and expression level for HEK293T cell-expressed *Rhodopsin*-family GPCRs. BRET$_{max}$ values for monomers are generally lower than those for dimers at similar expression levels. Monomeric receptors are shown in blue; dimers in red. The ambiguous cases C5R1 and DP2 are shown in pink. LTB4R1 is shown in green. Error bars indicate SEM for each parameter. CXCR4 is not shown for clarity. Absolute values are given in the Supplementary Data ("BRET Experiments").

(B) The *y*-intercept values obtained using type-3 BRET in the absence of competitor. Receptors behaving as monomers are shown in blue, dimers in red. C5R1 and DP2 are shown in pink, LTB4R1 in green. All *Rhodopsin*-family dimers identified in this study yielded a *y*-intercept value with a lower 95% confidence limit that is above zero, as did the ambiguous cases C5R1, DP2, and LTB4R1. Most of the monomeric receptors had *y*-intercept values that are not significantly non-zero, although a small number have lower 95% confidence limits greater than zero. Absolute values are given in the Supplementary Data ("BRET Experiments").

(C) C-terminal domain lengths of receptors in the monomer and dimer populations. The lack of significant difference between the two populations suggests that the observed monomers are genuine and are not the mis-assignment of dimers with large C-terminal domains that preclude efficient energy transfer.

(D) Expression profiles of monomers and dimers. The two profiles are highly similar, which indicates that the dimers are not artefacts of intracellular retention. C5R1, DP2, and LTB4R1 are not included. Expression categories correspond to those described in Table S1.

(E) Number of GPCRs in each studied family using the specified linker sequences between the receptor C terminus and the N terminus of GFP/Rluc. Linkers are provided for each receptor in the Supplementary Data ("R Primers" and "Non-R Primers").

(F) Number of *Rhodopsin*-family GPCRs studied using each linker sequence exhibiting each stoichiometric state.

See also Supplementary Data ("R Primers" and "Non-R Primers"), Table S1.

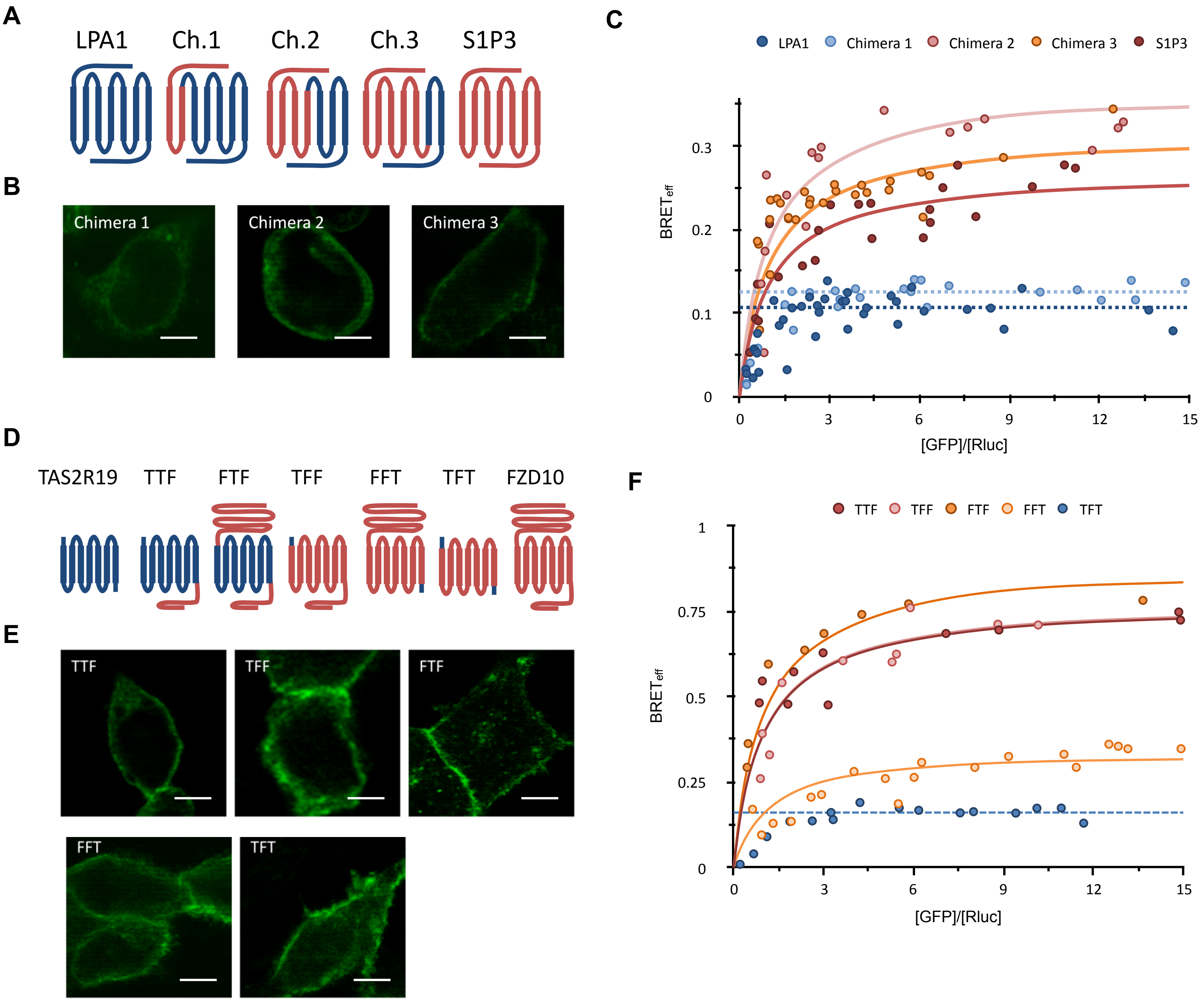
A
LPA1
Ch.1
Ch.2
Ch.3
S1P3
B
Chimera 1
Chimera 2
Chimera 3
C
LPA1
Chimera 1
Chimera 2
Chimera 3
S1P3
BRETeff
[GFP]/[Rluc]
D
TAS2R19
TTF
FTF
TFF
FFT
TFT
FZD10
E
TTF
TFF
FTF
FFT
TFT
F
TTF
TFF
FTF
FFT
TFT
BRETeff
[GFP]/[Rluc]

**Figure S5 Analysis of Chimeric Receptors Reveals Different Mechanisms of Dimerization between *Rhodopsin*- and *Frizzled*-Family Receptors, Related to Figures 3 & 4.**

(A) Schematic representations of the LPA1 (blue) and S1P3 (red) composition of each construct.

(B) Representative confocal microscopy images of HEK293T cells expressing the three LPA1/S1P3 chimeras from the pGFP$^2$ vector. All three constructs exhibit increased intracellular retention compared to the parent genes, but not aggregation and so all were suitable for use in BRET. Scale bars are 5 μm.

(C) Type-1 BRET analysis of three LPA1/S1P3 chimeras as well as the parent receptors (for comparison). For chimera 1, $BRET_{eff}$ is independent of [GFP]/[Rluc], indicative of monomeric behavior. Fits of LPA1 and chimera 1 data to a constant model are shown as broken lines. Chimeras 2 and 3 exhibit hyperbolic dependences of $BRET_{eff}$ on [GFP]/[Rluc] that fit well to a dimer model (solid lines). This indicates that S1P3 dimerization is dependent on motifs between EL1 and TM4.

(D) Schematic representations of chimeras of TAS2R19 (blue) and FZD10 (red). Chimeras were given a three-letter designation based on their composition, in which F and T denote FZD10 and TAS2R19 components, and the first, second, and third letters indicate the origin of the N-terminal domain, TM region, and C-terminal domain, respectively. Of the six possible combinations, only FTT failed to express sufficiently for BRET analysis.

(E) Representative confocal microscopy images of HEK293T cells expressing the 5 successfully expressed TAS2R19/FZD10 chimeras from the pGFP$^2$ vector. Aggregation was not apparent in any case and so all were suitable for use in BRET. Scale bars are 5 μm.

(F) Type-1 BRET analysis of TAS2R19/FZD10 chimeras indicates a role in dimerization of both the FZD10 N- and C-terminal domains. All chimeras containing either the FZD10 N- or C-terminal domains (TTF, TFF, FTF, and FFT) exhibited $BRET_{eff}$ dependence on [GFP]/[Rluc] in the manner predicted for a dimer. Replacement of the N- and C-terminal domains of FZD10 with those of TAS2R19 (chimera TFT) resulted in $BRET_{eff}$ being independent of [GFP]/[Rluc], indicating monomeric behavior. This suggests that the FZD10 TM region does not possess any inherent dimerization ability, in contrast to S1P3.

See also Supplementary Data ("BRET Experiments").

**Table S1. Qualitative Categories of GPCR Expression Based on Cellular Localization and Degree of Receptor Aggregation.**

| Category | Definition |
|---|---|
| A | Protein entirely in plasma membrane; almost no visible protein in internal membranes; no aggregation. |
| B | Protein almost entirely in plasma membrane; small amounts in internal membranes; no aggregation. |
| C | Majority of protein in plasma membrane; moderate amounts in internal membranes; no aggregation. |
| D | Some protein in plasma membrane; large amounts in internal membranes; no aggregation. |
| E | Some protein in plasma membrane; large amounts in internal membranes; small amounts of aggregation in some cells. |
| F | Some protein in plasma membrane; large amounts in internal membranes; small amounts of aggregation in most cells. |
| G | Little or no protein in plasma membrane or internal membranes; large amounts of aggregation in most cells. |

**Table S2. Transfection Conditions, HaloTag Spot Density, and Coincidence Values for Controls and GPCRs Analyzed Using SMCCCD in CHO K1 Cells.**

| Protein | HALO-tagged Construct (µg) | SNAP-tagged Construct (µg) | Post-transfection incubation (hours) | Cells imaged | No. experimental repeats | Mean HaloTag spots/cell ± SD | Mean % coincidence ± SEM | *p*-value of difference from CD86 |
|---|---|---|---|---|---|---|---|---|
| CD86 | 0.975 | 0.175 | 20 | 10 | 4 | 311±138 | 9.7±1.5 | - |
| CD28 | 0.975 | 0.175 | 48 | 8 | 3 | 288±85 | 28.2±3.7 | 0.0001 |
| LPA1 | 0.975 | 0.08 | 24 | 9 | 3 | 312±194 | 13.8±2.6 | 0.173 |
| S1P3 | 0.975 | 0.08 | 20 | 9 | 3 | 422±150 | 22.9±3.4 | 0.002 |
| $\beta_1$AR | 0.975 | 0.08 | 15 | 8 | 3 | 299±88 | 11.7±1.4 | 0.366 |
| $\alpha_{2C}$AR | 0.975 | 0.08 | 20 | 15 | 4 | 385±114 | 14.8±1.3 | 0.017 |

**Table S3. Published Reports of Homo-Oligomerization of the *Rhodopsin*-Family GPCRs Investigated in this Study.**

Twenty-six of the 60 receptors investigated have previously been reported to be homodimers in studies using a variety of approaches. GPCRs found here to be dimeric using type-1 and -3 BRET assays are underlined. Only reports of homo-oligomerization are included in this table; studies of heteromeric interactions are not shown. In most cases only one study per technique per receptor is cited.

| Receptor | Technique used to support homo-oligomerization |
|---|---|
| 5-$HT_{1D}$ | Co-IP (Salim et al., 2002), Western blotting (Lee et al., 2003) |
| 5-$HT_{2C}$ | Cysteine crosslinking (Mancia et al., 2008), Co-IP (Herrick-Davis et al., 2006), BiFC (Herrick-Davis et al., 2012), FCS (Herrick-Davis et al., 2012), FRET (Herrick-Davis et al., 2005; Herrick-Davis et al., 2006), Radioligand binding (Herrick-Davis et al., 2005; Herrick-Davis et al., 2006) |
| $A_{2A}R$ | BRET (Gandia et al., 2008), BiFC (Gandia et al., 2008; Vidi et al., 2008), FRET (Lukasiewicz et al., 2007) |
| $\underline{\alpha_{2C}AR}$ | BRET (Small et al., 2006), Co-IP (Small et al., 2006) |
| $AT_1$ | BRET (Hansen et al., 2004), Western blotting (AbdAlla et al., 2001; AbdAlla et al., 2004) |
| $\beta_1AR$ | BRET (Mercier et al., 2003), FRAP (Dorsch et al., 2009), X-ray crystallography (Huang et al., 2013), Single-molecule microscopy (Calebiro et al., 2013) |
| $B_2$ | Co-IP (Michineau et al., 2006), Western blotting (Michineau et al., 2006) |
| C5R1 | FRET (Floyd et al., 2003) |
| CCKBR | BRET (Cheng et al., 2003) |
| <u>CXCR4</u> | BiFC (Hammad et al., 2010), BRET (Babcock et al., 2003; Percherancier et al., 2005), Bivalent ligand crosslinking (Tanaka et al., 2010), X-ray crystallography (Wu et al., 2010) |
| EDNRA | Size-exclusion chromatography (Lee et al., 2012), FRET (Evans and Walker, 2008) |
| GPR50 | Co-IP (Levoye et al., 2006) |
| $\underline{H_1R}$ | Co-IP (Carrillo et al., 2003), FRET (Carrillo et al., 2003) |
| $\underline{H_2R}$ | Western blotting (Fukushima et al., 1997) |
| $LPA_1$ | β-galactosidase complementation (Zaslavsky et al., 2006) |
| $LPA_2$ | β-galactosidase complementation (Zaslavsky et al., 2006) |
| $LPA_3$ | β-galactosidase complementation (Zaslavsky et al., 2006) |
| LTB4R1 | Cysteine crosslinking (Baneres and Parello, 2003), Radioligand binding (Baneres and Parello, 2003), Cooperative ligand binding (Damian et al., 2008; Mesnier and Baneres, 2004) |
| $M_3$ | BRET (Nemoto and Toh, 2005) |
| NPY1R | FRET (Dinger et al., 2003) |
| OGR1 | β-galactosidase complementation (Zaslavsky et al., 2006) |
| $P2Y_2R$ | BRET (Suzuki et al., 2013), FRET (Kotevic et al., 2005) |
| $\underline{S1P_2}$ | β-galactosidase complementation (Zaslavsky et al., 2006), Co-IP (Van Brocklyn et al., 2002) |
| $\underline{S1P_3}$ | β-galactosidase complementation (Zaslavsky et al., 2006), Co-IP (Van Brocklyn et al., 2002) |
| SSTR2 | FRET (Grant et al., 2004), Western blotting (Grant et al., 2004) |
| TP | Co-IP (Laroche et al., 2005), ELISA (Laroche et al., 2005) |

## SUPPLEMENTARY EXPERIMENTAL PROCEDURES

### Cloning of chimeric GPCR constructs

Chimeras of the *S1PR3* and *LPAR1* genes were generated using multiple overlapping PCR reactions that in turn amplified the relevant segments of each gene and then extended them to produce the final construct. Domain boundaries for each gene were identified using the TMHMM v2.0 software from the Center for Biological Sequence Analysis, Technical University of Denmark. Oligonucleotide primers were designed to be complementary to the domain boundaries within the chimeras (sequences are given in Supplementary Data: "Non-R Primers"), *e.g.* S3-EL1, L1-TM3 is complementary to the final 15 nucleotides of the segment of *S1PR3* encoding EL1, immediately followed by a sequence complementary to the first 15 nucleotides of the segment of *LPAR1* encoding TM3. Individual fragments of each gene were amplified with the relevant combination of primers (*e.g.* TM3 of *LPAR1* was amplified using the S3-EL1, L1-TM3 F and L1-TM3, S3-IL2 R primers), and then combined by chimeric PCR in various combinations to generate full-length sequences (*e.g.* the *SLT3* chimera was generated by combining the gene fragments encoding *S1PR3* N terminus-EL1, *LPAR1* TM3, and *S1PR3* IL2-C terminus). Final PCR products were then inserted into the $pGFP^2$ vector using restriction digests of the *MluI* and *BamHI* sites, and their sequences confirmed using reversible terminator base sequencing. All constructs were then subcloned into the pRluc vector using the same restriction sites. All S1P3/LPA1 chimeras contained the receptor-GFP/Rluc linker sequence GDPPVAT.

Chimeras of the genes *FZD10* and *TAS2R19* were generated using multiple overlapping PCR reactions in the same manner as the S1P3/LPA1 chimeras. Oligonucleotide primers are given in the Supplementary Data: "Non-R Primers", and are named analogously to the *S1PR3/LPAR1* primers (*e.g.* F10-N, T19-TM is complementary to the first 15 nucleotides of *FZD10* and the first 15 nucleotides encoding the TM region in *TAS2R19*). Individual domain-encoding fragments were amplified individually and then combined in chimeric PCR reactions as described for the *S1PR3/LPAR1* constructs. The final PCR product was inserted into the $pGFP^2$ vector using restriction digests of the *MluI* and *BamHI* sites, and its sequence confirmed. All constructs were then subcloned into the pRluc vector using the same restriction sites, giving the receptor-GFP/Rluc linker sequence GDPPVAT.

### Quantitative flow cytometric analysis

Absolute receptor numbers were quantified using flow cytometric analysis of the GFP-tagged receptor variants. HEK293T cells were transiently transfected with 1 μg $pGFP^2$-GPCR vector per $6 \times 10^5$ cells using GeneJuice® (Novagen) in the same manner as for the type-1 BRET assay, and incubated for an equivalent length of time as the type-1 BRET assay (*i.e.* 24h for the majority of receptors, 48h for six exceptions). Transfected cells were analyzed by flow cytometry for GFP expression and their respective fluorescence converted into absolute protein numbers by reference to calibrated Quantibrite™ (BD Biosciences) beads. Data were collected for a total of $5 \times 10^4$ HEK293T cells for each GPCR-GFP fusion protein in each experiment, and viable single cells were gated-for using forward scatter, side scatter, and pulse width. GFP-positive cells were selected using a two-dimensional FL1 *vs* FL2 gate to prevent artefacts arising from cellular autofluorescence, and the geometric mean of GFP fluorescence determined for the GFP-positive population.

GFP fluorescence was converted into absolute receptor numbers by reference to a standard curve of GFP fluorescence *vs* surface protein expression generated for each experiment using HEK293T cells expressing a human CD2-GFP fusion protein labeled with phycoerythrin (PE)-conjugated mouse anti-human CD2 antibody (eBioscience 12-0029). Labeling was performed at an antibody concentration of 100 μg/ml to ensure saturating, monovalent binding to CD2. PE-GFP compensation was performed using unlabeled cells expressing CD2-GFP as a GFP-only control, and cells expressing CD2-Rluc labeled with PE-anti-CD2 as a PE-only control. PE fluorescence on the labeled CD2-GFP cells was then converted to antigen-binding events by reference to calibrated Quantibrite™ (BD Biosciences) PE beads as per the manufacturer's instructions. Absolute protein numbers were determined in this manner for each receptor in three independent replicate experiments.

**BRET assays**

6-well plates were seeded with $6 \times 10^5$ HEK293T cells in 2 ml DMEM (+ 10% FCS, 2 mM $_L$-glutamine) to ensure ~80% confluence after 24h. BRET constructs were co-transfected as BRET pairs consisting of both GFP- and Rluc-tagged proteins using GeneJuice® (Novagen) as per the manufacturer's instructions. DNA was always used at a final concentration of 0.05 μg/μl and total volume of 20 μl per well (*i.e.* 1 μg). In the type-1 BRET assay $pGFP^2$:pRluc ratios ranging from 1:2 to 66:1 were used as this gave the most useful spread of GFP:Rluc protein values. This was achieved by varying the volume of $pGFP^2$ and pRluc solutions in the final 20 μl volume; for example, a 3:1 $pGFP^2$:pRluc ratio would constitute 15 μl $pGFP^2$ (*i.e.* 0.75 μg) and 5 μl pRluc (*i.e.* 0.25 μg). In the type-3 assay, a 2:1 ratio of pU:($pGFP^2$+pRluc) was used to ensure an excess of competitor over labeled proteins, and a 12:1 $pGFP^2$:pRluc ratio was used to ensure measurable levels of BRET. In the majority of cases this was achieved using a transfection strategy of 1 μg pU, 0.462 μg $pGFP^2$, and 0.038 μg pRluc per well of $6 \times 10^5$ cells, however in cases of low receptor expression these amounts were increased to 2 μg pU, 0.924 μg $pGFP^2$, and 0.076 μg pRluc per well. Such an increase in DNA was required for 5-$HT_{2B}$, $AT_1$, $B_2$, CCR11, EDNRA, GPER, NPY1R, OR4D1, OXER1, and PAR1. 1 μg of expression vector for a soluble, fused form of Rluc-GFP (sGFP-Luc; PerkinElmer) was always used to transfect one well as the positive BRET control, and a negative control of mock-transfected cells (*i.e.* no DNA) was also always included.

Transfected HEK293T cells were collected from wells 24h after initial transfection and resuspended in PBS to a density of ~$1.5 \times 10^6$ cells/ml. $BRET_{eff}$ ratios were obtained by adding DeepBlueC (PerkinElmer) to a final concentration of 10 μM in 100 μl cell suspension in a 96-well OptiPlate (PerkinElmer) and collecting light emission in the BRET-A (410 ± 40 nm) and BRET-B (515 ± 15 nm) wavelengths. Collection for each wavelength was performed 3 times integrated over 1 second on a Fusion® Microplate Analyzer (PerkinElmer), thus giving a BRET-A and BRET-B value for each transfection. $BRET_{eff}$ values were calculated as BRET-B/BRET-A after background subtraction and correction for luciferase expression. $BRET_{eff}$ is normalized as a function of the $BRET_{eff}$ measured for the sGFP-Luc positive control, which was assigned a constant value of 1.

GFP expression was determined by exciting 100 μl of cells (in a new well) at 425 ± 25 nm and measuring emission at 515 ± 15 nm three times over 1 second, thereby giving the total fluorescence units (RFU). The same cells were then incubated for 2 min with 10 μM coelenterazine-*h* (final concentration) before measuring total emission 3 times over 1 second to give the total luminescence units (RLU). Acceptor/donor ratio was calculated using the

RLU/RFU value obtained from cell expressing the sGFP-Luc positive control, since the acceptor:donor ratio for this construct is one.

**Analysis of BRET data**

Analysis of all BRET data was performed using the Prism5 (GraphPad) software.

**(i) Type-1 assays**

Type-1 assay data were fitted to models of both dimeric (Equation 1) and monomeric (constant) behavior using the nonlinear least-squares regression function. The lower and upper range limits of [GFP]/[Rluc] values included in the analysis were 2 and 15, respectively, since 2 is the value at which $BRET_{eff}$ becomes independent of acceptor:donor ratio as confirmed with numerous controls (James et al., 2006), while 15 is the point at which the dimer model curve has flatted sufficiently to make it indistinguishable from a flat line within the typical error of the experiment. Restricting analysis within these thresholds therefore allows the most sensitive discrimination between monomer and dimer models. $BRET_{eff}$ data for [GFP]/[Rluc] values between 0 and 2 were included in plots for completeness, but were not used in either curve fitting or statistical analysis. The coefficient of determination ($R^2$) for the monomer fit is always zero as calculated $BRET_{eff}$ is a constant value. An $R^2$ value of less than zero for the dimer model indicates that it has a worse goodness-of-fit to the data than the monomer model, whereas an $R^2$ greater than zero indicates a better goodness-of-fit. Examples of monomer and dimer statistical outcomes are given in Figure S1.

$$\frac{\mathrm{BRET_{eff}}}{\mathrm{BRET_{max}}} = 1 - \frac{1}{(1+f)^{n-1}} \qquad \text{(Equation 1)}$$

Where:
$f$ = acceptor:donor ratio
$n$=stoichiometry
$BRET_{max}$ = maximal $BRET_{eff}$ achievable in each experiment

The relative expression level for all BRET assays was calculated as the combined expression of the GFP- and Rluc-tagged proteins expressed as a function of Rluc emission. This was achieved by converting GFP fluorescence units (FLU) into arbitrary Rluc luminescence (RLU) units using the GFP:Rluc ratio (*f*), whereupon total relative expression was calculated as the sum of GFP and Rluc luminescence units (Equation 2). In effect, this calculates the total RLU that would be expected if all GFP molecules in the sample were replaced with Rluc.

$$\mathrm{Expression}_{\mathrm{total}}^{\mathrm{RLU}} = \mathrm{GFP}^{\mathrm{RLU}} + \mathrm{Rluc}^{\mathrm{RLU}} \qquad \text{(Equation 2)}$$

$$\mathrm{GFP}^{\mathrm{RLU}} = f\,\mathrm{Rluc}^{\mathrm{RLU}}$$

Where:
$f$ = acceptor:donor ratio

Values for $f$ were determined individually for each sample by reference to the FLU and RLU values of the GFP-Rluc positive control, which has an inherent GFP:Rluc of 1 (Equation 3).

$$\frac{[\mathrm{GFP}]}{[\mathrm{Rluc}]} = \frac{\mathrm{RLU/FLU}}{\mathrm{RLU^{positive}/FLU^{positive}}} \qquad \text{(Equation 3)}$$

Total expression *vs* [GFP]/[Rluc] was plotted and fitted to a linear least-squares regression, then assessed for deviation from a non-zero slope using a Fisher F test ($p$<0.05 indicates a significant deviation from zero). The $p$ values are given in the Supplementary Data: "BRET Experiments", along with mean percentage slope as explained in Figure S1C.

**(ii) Type-3 assays**

All type-3 assay data were fitted using the linear least-squares regression function for total expression (from Equation 2) *vs* $BRET_{eff}$, generating separate fits for the data collected in the presence and absence of competitor. Goodness-of-fit was confirmed using the $R^2$ statistic and found to be high in all cases. $p^{diff}$ values were determined as the probability that the two datasets were from populations with identical $t$ distributions. The larger the $p^{diff}$ value, the lower the probability of difference between datasets, and hence dimers were defined as those receptors yielding a significant difference between the two conditions (Figure S1D).

## cAMP assay

Gs-coupled signaling by tagged and untagged $\beta_1$AR and $\beta_2$AR was assessed using the GloSensor cAMP Assay (Promega) as per the manufacturer's instructions. This was performed in CHO K1 cells in order to avoid the complication of natively expressed human receptors. CHO K1 cells were plated at $1 \times 10^5$ cells/well in 96-well plates. 24 h post plating, cells were transfected with 100 ng/well each of the expression vector for the receptor of interest and the pGloSensor-22F cAMP plasmid using GeneJuice (Novagen), as per the manufacturer's instructions. pGloSensor-22F encodes a variant of firefly luciferase containing a cAMP-binding moiety, binding of which to cAMP induces a conformational change leading to a 100 fold increase in light emission. 24 h after transfection, cells were equilibrated for 2 h with GloSensor cAMP reagent as per the manufacturer's instructions. Cells were incubated at room temperature with various concentrations of the partial agonist isoproterenol (Sigma Aldrich) for 5 min before light output was measured using a Fusion Microplate Analyzer (PerkinElmer).

All cAMP assay data were corrected for background luciferase emission by subtraction of values from cells in the absence of agonist. Changes in light emission were normalized to a percentage maximal response for each read. Mean percentage response and SEM values were calculated for each agonist concentration and then fitted to a nonlinear regression normalized dose-response stimulation model: response ($x$) = $100/(1 + 10^{\log(EC50-x)})$.

## SUPPLEMENTAL REFERENCES


AbdAlla, S., Lother, H., el Massiery, A., and Quitterer, U. (2001). Increased AT(1) receptor heterodimers in preeclampsia mediate enhanced angiotensin II responsiveness. Nat Med *7*, 1003-1009.

AbdAlla, S., Lother, H., Langer, A., el Faramawy, Y., and Quitterer, U. (2004). Factor XIIIA transglutaminase crosslinks AT(1) receptor dimers of monocytes at the onset of atherosclerosis. Cell *119*, 343-354.

Babcock, G.J., Farzan, M., and Sodroski, J. (2003). Ligand-independent dimerization of CXCR4, a principal HIV-1 coreceptor. J Biol Chem *278*, 3378-3385.

Baneres, J.L., and Parello, J. (2003). Structure-based analysis of GPCR function: Evidence for a novel pentameric assembly between the dimeric leukotriene B-4 receptor BLT1 and the G-protein. J Mol Biol *329*, 815-829.

Calebiro, D., Rieken, F., Wagner, J., Sungkaworn, T., Zabel, U., Borzi, A., Cocucci, E., Zuern, A., and Lohse, M.J. (2013). Single-molecule analysis of fluorescently labeled G-protein-coupled receptors reveals complexes with distinct dynamics and organization. Proc Natl Acad Sci U S A *110*, 743-748.

Carrillo, J.J., Pediani, J., and Milligan, G. (2003). Dimers of class A G protein-coupled receptors function via agonist-mediated trans-activation of associated G proteins. J Biol Chem *278*, 42578-42587.

Cheng, Z.J., Harikumar, K.G., Holicky, E.L., and Miller, L.J. (2003). Heterodimerization of type A and B cholecystokinin receptors enhance signaling and promote cell growth. J Biol Chem *278*, 52972-52979.

Damian, M., Mary, S., Martin, A., Pin, J.-P., and Baneres, J.-L. (2008). G protein activation by the leukotriene B(4) receptor dimer - Evidence for an absence of trans-activation. J Biol Chem *283*, 21084-21092.

Dinger, M.C., Bader, J.E., Kobor, A.D., Kretzschmar, A.K., and Beck-Sickinger, A.G. (2003). Homodimerization of neuropeptide y receptors investigated by fluorescence resonance energy transfer in living cells. J Biol Chem *278*, 10562-10571.

Dorsch, S., Klotz, K.N., Engelhardt, S., Lohse, M.J., and Bunemann, M. (2009). Analysis of receptor oligomerization by FRAP microscopy. Nat Methods *6*, 225-230.

Evans, N.J., and Walker, J.W. (2008). Endothelin receptor dimers evaluated by FRET, ligand binding, and calcium mobilization. Biophys J *95*, 483-492.

Floyd, D.H., Geva, A., Bruinsma, S.P., Overton, M.C., Blumer, K.J., and Baranski, T.J. (2003). C5a receptor oligomerization - II. Fluorescence resonance energy transfer studies of a human G protein-coupled receptor expressed in yeast. J Biol Chem *278*, 35354-35361.

Fukushima, Y., Asano, T., Saitoh, T., Anai, M., Funaki, M., Ogihara, T., Katagiri, H., Matsuhashi, N., Yazaki, Y., and Sugano, K. (1997). Oligomer formation of histamine H2 receptors expressed in Sf9 and COS7 cells. FEBS Lett *409*, 283-286.

Gandia, J., Galino, J., Amaral, O.B., Soriano, A., Lluis, C., Franco, R., and Ciruela, F. (2008). Detection of higher-order G protein-coupled receptor oligomers by a combined BRET-BiFC technique. FEBS Lett *582*, 2979-2984.

Grant, M., Collier, B., and Kumar, U. (2004). Agonist-dependent dissociation of human somatostatin receptor 2 dimers - A role in receptor trafficking. J Biol Chem *279*, 36179-36183.

Hammad, M.M., Kuang, Y.Q., Yan, R., Allen, H., and Dupre, D.J. (2010). Na+/H+ Exchanger Regulatory Factor-1 Is Involved in Chemokine Receptor Homodimer CCR5 Internalization and Signal Transduction but Does Not Affect CXCR4 Homodimer or CXCR4-CCR5 Heterodimer. J Biol Chem *285*, 34653-34664.

Hansen, J.L., Theilade, J., Haunso, S., and Sheikh, S.P. (2004). Oligomerization of wild type and nonfunctional mutant angiotensin II type I receptors inhibits G alpha(q) protein signaling but not ERK activation. J Biol Chem *279*, 24108-24115.

Herrick-Davis, K., Grinde, E., Harrigan, T.J., and Mazurkiewicz, J.E. (2005). Inhibition of serotonin 5-hydroxytryptamine2C receptor function through heterodimerization - Receptor dimers bind two molecules of ligand and one G-protein. J Biol Chem *280*, 40144-40151.

Herrick-Davis, K., Grinde, E., Lindsley, T., Cowan, A., and Mazurkiewicz, J.E. (2012). Oligomer Size of the Serotonin 5-Hydroxytryptamine 2C (5-HT2C) Receptor Revealed by Fluorescence Correlation Spectroscopy with Photon Counting Histogram Analysis. J Biol Chem *287*, 23604-23614.

Herrick-Davis, K., Weaver, B.A., Grinde, E., and Mazurkiewicz, J.E. (2006). Serotonin 5-HT2C receptor homodimer biogenesis in the endoplasmic reticulum - Real-time visualization with confocal fluorescence resonance energy transfer. J Biol Chem *281*, 27109-27116.

Huang, J., Chen, S., Zhang, J.J., and Huang, X.-Y. (2013). Crystal structure of oligomeric beta(1)-adrenergic G protein-coupled receptors in ligand-free basal state. Nature Structural & Molecular Biology *20*, 419-425.

James, J.R., Oliveira, M.I., Carmo, A.M., Iaboni, A., and Davis, S.J. (2006). A rigorous experimental framework for detecting protein oligomerization using bioluminescence resonance energy transfer. Nat Methods *3*, 1001-1006.

Kotevic, I., Kirschner, K.M., Porzig, H., and Baltensperger, K. (2005). Constitutive interaction of the P2Y(2) receptor with the hematopoietic cell-specific G protein G(alpha 16) and evidence for receptor oligomers. Cell Signal *17*, 869-880.

Laroche, G., Lepine, M.C., Theriault, C., Giguere, P., Giguere, V., Gallant, M.A., de Brum-Fernandes, A., and Parent, J.L. (2005). Oligomerization of the alpha and beta isoforms of the thromboxane A(2) receptor: Relevance to receptor signaling and endocytosis. Cell Signal *17*, 1373-1383.

Lee, K., Jung, Y., Lee, J.Y., Lee, W.K., Lim, D., and Yu, Y.G. (2012). Purification and characterization of recombinant human endothelin receptor type A. Protein Expr Purif *84*, 14-18.

Lee, S.P., O'Dowd, B.F., Rajaram, R.D., Nguyen, T., and George, S.R. (2003). D2 dopamine receptor homodimerization is mediated by multiple sites of interaction, including an intermolecular interaction involving transmembrane domain 4. Biochemistry *42*, 11023-11031.

Levoye, A., Dam, J., Ayoub, M.A., Guilllaume, J.L., Couturier, C., Delagrange, P., and Jockers, R. (2006). The orphan GPR50 receptor specifically inhibits MT1 melatonin receptor function through heterodimerization. Embo J *25*, 3012-3023.

Lukasiewicz, S., Blasiak, E., Faron-Gorecka, A., Polit, A., Tworzydlo, M., Gorecki, A., Wasylewski, J., and Dziedzicka-Wasylewska, M. (2007). Fluorescence studies of homooligomerization of adenosine A(2A) and serotonin 5-HT1A receptors reveal the specificity of receptor interactions in the plasma membrane. Pharmacol Rep *59*, 379-392.

Mancia, F., Assur, Z., Herman, A.G., Siegel, R., and Hendrickson, W.A. (2008). Ligand sensitivity in dimeric associations of the serotonin 5HT2c receptor. EMBO Rep *9*, 363-369.

Mercier, J.F., Salahpour, A., Angers, P., Breit, A., and Bouvier, M. (2003). Quantitative assessment of beta(1)- and beta(2)-adrenergic re-receptor homo- and heterodimerization by bioluminescence resonance energy transfer J Biol Chem *278*, 18704-18704.

Mesnier, D., and Baneres, J.L. (2004). Cooperative conformational changes in a G-protein-coupled receptor dimer, the leukotriene B-4 receptor BLT1. J Biol Chem *279*, 49664-49670.

Michineau, S., Alhenc-Gelas, F., and Rajerison, R.M. (2006). Human bradykinin B2 receptor sialylation and N-glycosylation participate with disulfide bonding in surface receptor dimerization. Biochemistry *45*, 2699-2707.

Nemoto, W., and Toh, H. (2005). Prediction of interfaces for oligomerizations of G-protein coupled receptors. Proteins *58*, 644-660.

Percherancier, Y., Berchiche, Y.A., Slight, I., Volkmer-Engert, R., Tamamura, H., Fujii, N., Bouvier, M., and Heveker, N. (2005). Bioluminescence resonance energy transfer reveals ligand-induced conformational changes in CXCR4 homo- and heterodimers. J Biol Chem *280*, 9895-9903.

Salim, K., Fenton, T., Bacha, J., Urien-Rodriguez, H., Bonnert, T., Skynner, H.A., and Watts, E. (2002). Oligomerization of G-protein-coupled receptors shown by selective co-immunoprecipitation. J Biol Chem *277*, 15482-15485.

Small, K.M., Schwarb, M.R., Glinka, C., Theiss, C.T., Brown, K.M., Seman, C.A., and Liggett, S.B. (2006). Alpha(2A)- and alpha(2C)-adrenergic receptors form homo- and heterodimers: The heterodimeric state impairs agonist-promoted GRK phosphorylation and beta-arrestin recruitment. Biochemistry *45*, 4760-4767.

Suzuki, T., Namba, K., Mizuno, N., and Nakata, H. (2013). Hetero-oligomerization and specificity changes of G protein-coupled purinergic receptors: novel insight into diversification of signal transduction. Methods in enzymology *521*, 239-257.

Tanaka, T., Nomura, W., Narumi, T., Masuda, A., and Tamamura, H. (2010). Bivalent Ligands of CXCR4 with Rigid Linkers for Elucidation of the Dimerization State in Cells. J Am Chem Soc *132*, 15899-15901.

Van Brocklyn, J.R., Behbahani, B., and Lee, N.H. (2002). Homodimerization and heterodimerization of SlP/EDG sphingosine-1-phosphate receptors. Biochim Biophys Acta Mol Cell Biol Lipids *1582*, 89-93.

Vidi, P.A., Chen, J.J., Irudayaraj, J.M.K., and Watts, V.J. (2008). Adenosine A(2A) receptors assemble into higher-order oligomers at the plasma membrane. FEBS Lett *582*, 3985-3990.

Wu, B.L., Chien, E.Y.T., Mol, C.D., Fenalti, G., Liu, W., Katritch, V., Abagyan, R., Brooun, A., Wells, P., Bi, F.C., *et al.* (2010). Structures of the CXCR4 Chemokine GPCR with Small-Molecule and Cyclic Peptide Antagonists. Science *330*, 1066-1071.

Zaslavsky, A., Singh, L.S., Tan, H.Y., Ding, H.W., Liang, Z.C., and Xu, Y. (2006). Homo- and hetero-dimerization of LPA/S1P receptors, OGR1 and GPR4. Biochim Biophys Acta Mol Cell Biol Lipids *1761*, 1200-1212.
